# Robust chromatin state annotation

**DOI:** 10.1101/gr.278343.123

**Published:** 2024-03

**Authors:** Mehdi Foroozandeh Shahraki, Marjan Farahbod, Maxwell W. Libbrecht

**Affiliations:** School of Computing Science, Simon Fraser University, Burnaby, British Columbia V51 1S6, Canada

## Abstract

With the goal of mapping genomic activity, international projects have recently measured epigenetic activity in hundreds of cell and tissue types. Chromatin state annotations produced by segmentation and genome annotation (SAGA) methods have emerged as the predominant way to summarize these epigenomic data sets in order to annotate the genome. These chromatin state annotations are essential for many genomic tasks, including identifying active regulatory elements and interpreting disease-associated genetic variation. However, despite the widespread applications of SAGA methods, no principled approach exists to evaluate the statistical significance of chromatin state assignments. Here, we propose the first method for assigning calibrated confidence scores to chromatin state annotations. Toward this goal, we performed a comprehensive evaluation of the reproducibility of the two most widely used existing SAGA methods, ChromHMM and Segway. We found that their predictions are frequently irreproducible. For example, when applying the same SAGA method on two sets of experimental replicates, 27%–69% of predicted enhancers fail to replicate. This suggests that a substantial fraction of predicted elements in existing chromatin state annotations cannot be relied upon. To remedy this problem, we introduce SAGAconf, a method for assigning a measure of confidence (*r*-value) to chromatin state annotations. SAGAconf works with any SAGA method and assigns an *r*-value to each genomic bin of a chromatin state annotation that represents the probability that the label of this bin will be reproduced in a replicated experiment. Thus, SAGAconf allows a researcher to select only the reliable predictions from a chromatin annotation for use in downstream analyses.

Annotating regulatory elements in the genome is fundamental to answering key questions, including those on the molecular basis of disease, evolution, cellular differentiation, and development. To this end, international mapping projects have recently measured epigenetic activity in hundreds of cell and tissue types using genome-wide assays such as ChIP-seq (The ENCODE Project Consortium 2012; Roadmap Epigenomics Consortium et al. 2015).

Chromatin state annotations produced by segmentation and genome annotation (SAGA) methods have emerged as the predominant way to summarize epigenomic data sets in order to annotate the genome. SAGA methods include Segway (Hoffman et al. 2012), ChromHMM (Ernst and Kellis 2012), and others (Day et al. 2007; Biesinger et al. 2013; Zhang et al. 2016; Zhang and Hardison 2017; for review, see Libbrecht et al. 2021). They take a collection of epigenomic assay data sets from a given cell type or tissue as input and partition the genome into segments with similar patterns in the input data sets. The output is an annotation that assigns a label to each genomic position. These algorithms use probabilistic graphical models such as hidden Markov models. They are unsupervised in the sense that the model identifies patterns in the data, and the researcher later maps each pattern to putative biological functions such as enhancer, promoter, or transcribed gene. Thus, SAGA algorithms are used to distill complex data into an interpretable summary of genomic activity. SAGA algorithms have been broadly applied. Large-scale epigenome mapping projects such as ENCODE (The ENCODE Project Consortium 2012) and Roadmap (Roadmap Epigenomics Consortium et al. 2015) produced them as their primary output, and researchers have now produced reference SAGA annotations for hundreds of cell types (Libbrecht et al. 2019; Zhang and Mahony 2019; Boix et al. 2021; Vu and Ernst 2022)

Previous efforts to evaluate the reliability of SAGA chromatin state annotations have found mixed results. On one hand, SAGA annotations recapitulate known genome biology, and the annotations accurately represent many genomic phenomena, including transcription (Zhang and Hardison 2017; Libbrecht et al. 2019, 2021). However, results are often dissimilar between SAGA methods and hyperparameter settings of a given method (Hoffman et al. 2013; Zhang and Hardison 2017; Libbrecht et al. 2019, 2021). This suggests that some aspects of SAGA annotations do not reflect true biology.

Thus, there is a great need for a way to produce robust chromatin state annotations. Currently, there is no principled way to evaluate the statistical significance of SAGA label assignments. SAGA annotations are not the result of a statistical test, so they do not carry a *P*-value. SAGA methods use probabilistic graphical models that output posterior probabilities; however, in practice, these posterior probabilities are vastly overconfident, resulting in most positions receiving a posterior probability *>*99% (Chan et al. 2018; Libbrecht et al. 2021).

Here, we propose the first method for assigning reliable confidence scores to SAGA annotations. We base our approach on evaluating the reproducibility of annotations across replicates (Impagliazzo et al. 2022). We are motivated by methods for ChIP-seq peak calling, for which researchers generally use irreproducible discovery rate (IDR) analysis to assign confidence scores to peaks (The ENCODE Project Consortium 2011; Li et al. 2011; Landt et al. 2012; Bailey et al. 2013). In IDR analysis, researchers score putative peaks according to their reproducibility in two or more experimental replicates, with the expectation that peak findings are expected to consistently rank highly in both experiments. Note that, although several methods have been developed for the related task of comparing sets of SAGA annotations to one another, to our knowledge no existing method can assign confidence estimates to a single chromatin state annotation. In particular, ChromDiff (Yen and Kellis 2015), SCIDDO (Ebert and Schulz 2021), and EpiCompare (He and Wang 2017) perform the task of group-wise comparative analysis, in which they take as input two sets of annotations and identify differential labels between the two sets. Related methods EpiAlign (Ge et al. 2019) and Chromswitch (Jessa and Kleinman 2018) similarly perform group-wise comparative analysis but are primarily designed to compare chromatin state patterns within a query region. CSREP (Vu et al. 2023) performs the task of group summarization, taking annotations for a group of samples as input and probabilistically estimating the state at each genomic position to derive a representative chromatin state map for the group. Because all of these existing studies analyze patterns across annotations of many cell types, they cannot estimate the confidence in the annotation of a single target cell type.

Toward the goal of understanding robustness of SAGA annotations, here we perform a comprehensive evaluation of the reproducibility of SAGA methods. As described below, we show that SAGA annotations show a large degree of disagreement, even when run with the same method on replicated data sets. Much of this disagreement can be attributed to inconsequential factors such as the granularity of label definitions and mismatch in the boundaries of annotated elements. Yet, much disagreement remains, suggesting that a substantial fraction of element annotations produced by SAGA cannot be relied upon as they are not reproduced by a replicate analysis.

To remedy this problem, we introduce SAGAconf, a method for assigning a measure of confidence to SAGA annotations. SAGAconf takes as input two sets of annotations from replicated experiments and compares them, operating on the premise that ideally, replicated data should yield identical annotations. SAGAconf computes a confidence score for the label of per genomic position, called the *r*-value. This *r*-value represents the probability that the annotation at a specific location will be reproduced in a replicated study. By applying a threshold on the *r*-value (usually 0.9 or 0.95), researchers obtain a confident subset of the genome annotation for downstream analysis, thereby enhancing the reliability and robustness of their research.

## Results

### Comprehensive evaluation of chromatin state reproducibility

We performed a comprehensive evaluation of reproducibility of SAGA annotations (Methods). We collected replicated pairs of epigenomic data sets in five cell types. In each replicate pair, we applied a SAGA pipeline to produce two replicate annotations, which we term the “base” and “verification” annotation, respectively. For the purposes of exposition, we focus our analysis on a running example: ChromHMM run on GM12878 (under setting 1 described below: different data, different models).

**Figure 1.**
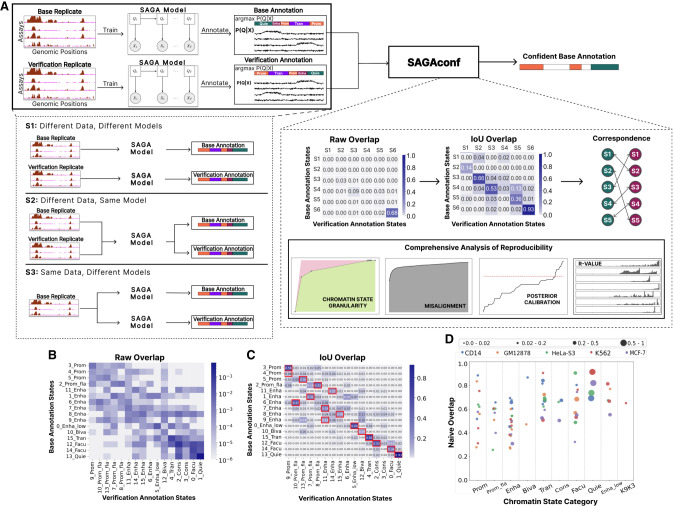
Schematic workflow. (*A*) We obtained histone modification assays from biological replicates via ENCODE DCC (The ENCODE Project Consortium 2012) and used these data sets to train SAGA models (Segway or ChromHMM) to generate chromatin state annotations. The SAGA model outputs a matrix representing the posterior probability *P*(*Q*|*X*) values of assigning each chromatin state to each genomic position and a vector of state labels assigned to the position with the highest posterior probability, argmax*P*(*Q*|*X*) (Methods). One set of replicated data is chosen as the base and the other as the verification. SAGA training and genome annotation are performed according to three settings of variability: S1 (different data, different models), in which two separate SAGA models are trained independently using data from each biological replicate; S2 (different data, same model), in which data from both replicates are concatenated to train a single SAGA model that provides separate annotations for each replicate; and S3 (same data, different models), in which the same data set (base replicate) is used to train two different SAGA models with different parameter initializations. Both the base and verification annotations, generated by any variability setting, are inputs to SAGAconf. The SAGAconf evaluation pipeline begins by forming a pairwise overlap frequency distribution matrix between the two annotations and calculating the intersection over union (IoU) overlap to determine the correspondence between state pairs across the annotations. SAGAconf performs reproducibility analysis and outputs a subset of the base annotation that it identifies as confident (Methods). (*B*) The raw overlap frequency distribution from our running example annotation (S1; ChromHMM, GM12878). Rows and columns correspond to states in base and verification annotations, respectively. Color indicates frequency of overlap (log scale). (*C*) Same as *B*, but color indicates the IoU of overlap is derived from a raw overlap matrix (linear scale). For each chromatin state of the base annotation, its corresponding state in the verification annotation is defined as the one with the maximum IoU (marked with red square). (*D*) Fraction of overlap (Naive overlap) of various chromatin states categories identified in the ChromHMM annotations according to S1 for all five cell types. Each dot represents a chromatin state, with color denoting cell type and size proportional to genome coverage.

A SAGA model assigns an integer ID (1..*k*) to each genomic bin. These IDs represent different chromatin states, also known as “states” (Fig. 1A). The relationship of a pair of annotations can be characterized by a *k* × *k* matrix representing the frequency of overlap of each base state with each verification state (Fig. 1B). Because SAGA methods are unsupervised, the output integer-state IDs do not directly correspond across two SAGA models. Thus, we must first detect corresponding states across annotations to enable comparison (Fig. 1C). To establish a correspondence between chromatin states while accounting for varying genomic coverages, we calculate intersection over union (IoU) of overlap, also known as Jaccard similarity (Fig. 1C), and for each base state, we define the corresponding verification state to be the one with the highest IoU (Fig. 1A,C).

Irreproducibility between annotations may be caused either by differences in input data replicates or by differences in model training. To delineate among these sources of irreproducibility, we assess pairs of experiments in three different settings of variability (Methods) (Fig. 1A). In setting 1 (different data, different models), we trained two separate SAGA models using data from separate biological replicates, simulating the case in which independent researchers each perform a SAGA analysis. In setting 2 (different data, same model), we train a single SAGA model and use it to annotate each replicate (known as “concatenated” annotation). In this setting, the shared model contains one set of state definitions used for both annotations, making the matching step (calling corresponding states) effectively irrelevant. This approach removes elements of variability in training and state matching, isolating the irreproducibility that stems solely from the input data rather than from the model training or random state initialization. In setting 3 (same data, different models), the same data set from one of the biological replicates is used to train two different SAGA models but with a different random parameter initialization in each model. This setting isolates irreproducibility owing to model training.

Because SAGA methods are unsupervised, each integer-state label must be assigned a putative biological interpretation such as “promoter” or “enhancer.” To avoid bias stemming from manual interpretation, we used a previously described automatic process to assign a vocabulary of human-readable chromatin state categories (Supplemental Material, Sec. 1.3; Libbrecht et al. 2019; Farahbod et al. 2023). Note that interpretations are for exposition only, and all analyses were performed at the level of the states, not interpreted chromatin state categories. Because it is automated, the interpretation process may be imperfect; for example, in our running example, base state 6_Enha(B) overlaps with verification state 10_Prom_fla(V), and the mismatch in chromatin state category likely results from an error in the interpreter, not the annotations themselves.

### Pairwise overlap does not fully capture the reproducibility profile of SAGA annotations

We found substantial differences between annotations of the two replicates (Fig. 1B–D). Overall, in our running example, only ∼80% of genomic bins are annotated with the corresponding label in the verification annotation (Fig. 3H). Overlap is poorest for punctate chromatin states such as promoter, enhancer, and bivalent (∼50% overlap) (Fig. 1D). Overlap for broad chromatin states such as transcribed, facultative, and constitutive heterochromatin is higher (∼70% overlap) (Fig. 1D) and is best for quiescent (∼80% overlap) (Fig. 1D). These results are generally consistent across the five cell types we tested (Fig. 3H). Overlap is significantly lower for Segway, which achieves just a 30%–40% average overlap (Fig. 3H). These results are concerning for the application of SAGA annotations, as reproducibility is far lower than the 95% standard used for most genomic predictions. Results are similar in other cell types and settings (Supplemental Figs. 1–8). In setting 2 (different data, same model), we observe a slight improvement in the naive overlap, over setting 1 (different model, different data), for ChromHMM annotations but not for Segway (Fig. 3H). However, in setting 3, in which we used the same data with different models, we observed a significant improvement in the average overlap, especially for Segway annotations (Fig. 3H). This suggests that the quality of data plays a more significant role in the poor naive overlap observed in settings 1 and 2 than in the model training. However, as we describe below, measuring reproducibility according to the naive overlap with the corresponding states may be too conservative, as doing so counts two types of variation that may not be important to practitioners.

### SAGA models can learn multiple similar states that are not confidently distinguishable from one another

**Figure 2.**
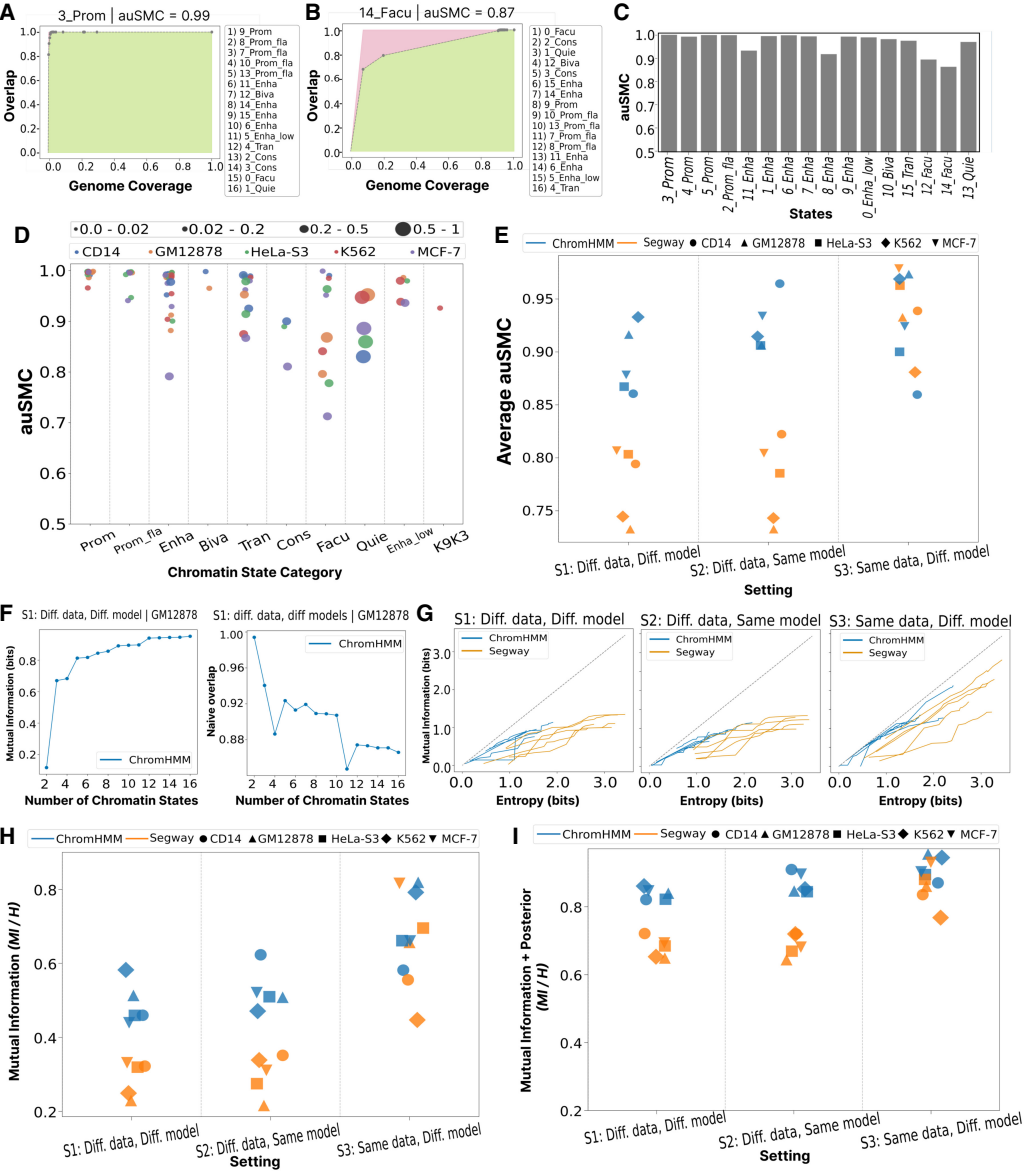
Evaluation of reproducibility as a function of granularity of chromatin state. (*A*) For a given state in the base annotation *B*_*i*_, we ordered all states in the verification annotation *V* according to their IoU overlap. The *leftmost* dot indicates the most-overlapped verification state, *V*_*j*_; the horizontal axis indicates *V*_*j*_’s genomic coverage; and the vertical axis indicates the fraction of *B*_*i*_ overlapping with *V*_*j*_. The second point corresponds to the union of *V*_*j*_ with the second-most enriched verification state, and so on for subsequent points. This forms an ROC-like curve, with the green area representing the area under the curve (AUC) for the observed overlap versus genomic coverage of each state, and the red area indicating deviation from perfect reproducibility in which the first verification state covers all positions of target state *B*_*i*_. The area under the state-merging curve (auSMC) ratio is a numerical representation of reproducibility as a function of chromatin state granularity, which is calculated by dividing the observed AUC by the AUC pertaining to the perfect reproducibility. In other words, a larger red area corresponds to lower auSMC. Results are shown for the 3_Prom(B) chromatin state in the base annotation obtained from our running example (S1; ChromHMM, GM12878). Lists of chromatin state names on the *right* of *A* represent states in the verification annotation sorted according to their IoU overlap with the target chromatin state in the base annotation. (*B*) Same as *A*, but for facultative heterochromatin 14_Facu(B). (*C*) auSMC ratio of chromatin states in the running example (S1; ChromHMM, GM12878). (*D*) auSMC ratio of various chromatin states categories identified in the ChromHMM annotation according to S1 (different data, different models) for five cell types. Each dot represents a chromatin state, with color denoting cell type and size proportional to genome coverage. (*E*) Average auSMC ratio (weighted by the genome coverage) across two SAGA models, five cell types, and three settings. Color denotes the SAGA model, and shapes represent cell types. (*F*) Mutual information (*left*) and naive overlap (*right*) as a function of the number of chromatin states, for our running example (S1; ChromHMM, GM12878). The horizontal axis indicates the number of states; the default 16-label model is on the far *right*, and each dot to the *left* represents an annotation after merging two labels in the annotation to its *right*. Mutual information indicates the number of bits of information about the verification state that is gained by observing the base state (Methods). (*G*) Mutual information between base and verification replicates after merging labels, as a function of the entropy of the base annotation. In a perfectly reproducible case, the amount of mutual information would be equal to the entropy. Color denotes SAGA model (Segway or ChromHMM). (*H*) Mutual information between base and verification annotations across two SAGA models, five cell types, and three settings as a fraction of the base annotation entropy. (*I*) Same as *H*, but evaluating the mutual information when observing both the label and posterior probability (Methods).

Each base state generally overlaps with multiple verification states, yet this overlap usually occurs among a small number of related states (Fig. 1C). For example, in our running example, base state 7_Enha(B) frequently overlaps verification states 6_Enha(V), 11_Enha(V), and 15_Enha(V). A similar issue occurs across related chromatin state types: base state 10_Biva(B) overlaps a mixture of verification states 12_Biva(V) and 13_Prom_fla(V). This suggests that irreproducibility may stem from excessive granularity of states. That is, dividing the genome among 16 labels as performed by existing SAGA pipelines (Methods) requires a resolution in chromatin states that is too fine to achieve robust results. There is a trade-off between reproducibility and the number of states; a two-state annotation is likely to have near-perfect reproducibility but carries little information (Fig. 2F,G).

Thus, we evaluated whether each base annotation state can be recovered using multiple verification annotation states. To accomplish this, for each base state, we ordered verification states according to their IoU overlap and then iteratively merged the top two states in terms of IoU overlap to create a “superstate” in the verification annotation that eventually covers the entire genome. This process produces a receiver-operating characteristic-like (ROC-like) curve showing the fraction of overlap versus genomic coverage of each state. The area under this state-merging curve (auSMC) is a measure of a state's reproducibility when taking into account such merges, calculated according to the ratio of the observed area under the curve (Fig. 2A–C, shaded green) to the area under the curve pertaining to the perfect reproducibility (shaded red).

We found that, indeed, base chromatin states can often be recovered using a combination of verification states. For example, although only 80% of base state 3_Prom(B) is overlapped by its corresponding verification state 9_Prom(V), a further 10% overlaps related label 8_Prom_fla(V), resulting in auSMC > 0.99 (Fig. 2A). Both SAGA models are almost always able to identify enhancer chromatin states with auSMC > 0.8 (Supplemental Figs. 9, 10, 13). However, promoter chromatin states identified by ChromHMM generally show strong auSMC (Fig. 2D), whereas for Segway, they often show a poor auSMC, even in S3 (same data, different models) (Supplemental Figs. 9, 10, 13). However, some base states are not reproducible even when accounting for multiple verification states. For example, although 68% of base state 14_Facu(B) is overlapped by its corresponding verification state 0_Facu(V), one must merge both other repressive states (2_Cons(V) and 1_Quie(V)), covering most of the rest of the genome, in order for the merged state to cover >90% of 14_Facu(B), resulting in aucSMC = 0.87. Facultative heterochromatin has the largest variation in terms of auSMC, likely owing to differing thresholds dividing the quiescent and facultative heterochromatin states (Supplemental Figs. 11, 12). As with naive overlap, settings 1 and 2 (different data, different models and different data, same model, respectively) have similar performance, but setting 3 (same data, different model) performs much better, especially for Segway, indicating that most differences are caused by differences in the input data (Fig. 2E).

Information theory provides a more general way of evaluating reproducibility while accounting for the lack of one-to-one correspondence (Methods). The entropy of an annotation (measured in bits) represents the amount of information it conveys, when accounting for the number of labels and their frequency in the genome. An annotation with 16 labels that are evenly distributed in the genome has 4 bits of entropy. The mutual information (also measured in bits) indicates the amount of information about the base annotation one garners from the verification annotation (Methods). We found that, for our running example, the base annotation conveys 0.95 bit of mutual information about the verification annotation, out of two total bits of entropy (Fig. 2H). In general, for both the ChromHMM and Segway annotations, the base annotation usually conveys ∼1 bit of information about the verification annotation (Figs. 2G,H).

These results suggest that applying SAGA methods using too many labels provides too much granularity in the definition of states, such that the models learn multiple similar states that are not confidently distinguishable from one another. This suggests that reducing the number of states may give more robust annotations. It is common for practitioners to merge similar states as a postprocessing step of SAGA annotations (Libbrecht et al. 2015, 2019). Running SAGA with all possible numbers of states would be computationally infeasible, and analysis likely would suffer from noise owing to model training. Therefore, we developed an automatic state-merging strategy simulating annotations with fewer states by merging states in the base and verification replicates, respectively (Methods). We iteratively merged pairs of state in each annotation until we were left with two states in each annotation, in which at each iteration we merged the pairs, resulting in the smallest loss of mutual information (Methods) (Fig. 2F–H). As merging low-coverage labels has a smaller impact than merging high-coverage labels, throughout our analysis below we use the bits of entropy to estimate the complexity of an annotation instead of the raw number of states (Fig. 2G). We found that SAGA annotations with fewer labels have greatly improved reproducibility. In our running example, moving from a 16-state to a 10-state annotation improves naive overlap from 87% to 91% while only reducing the entropy of the annotation from 1.56 to 1.37 bits (Fig. 2F).

### Spatial misalignment of corresponding chromatin states leads to irreproducibility

**Figure 3.**
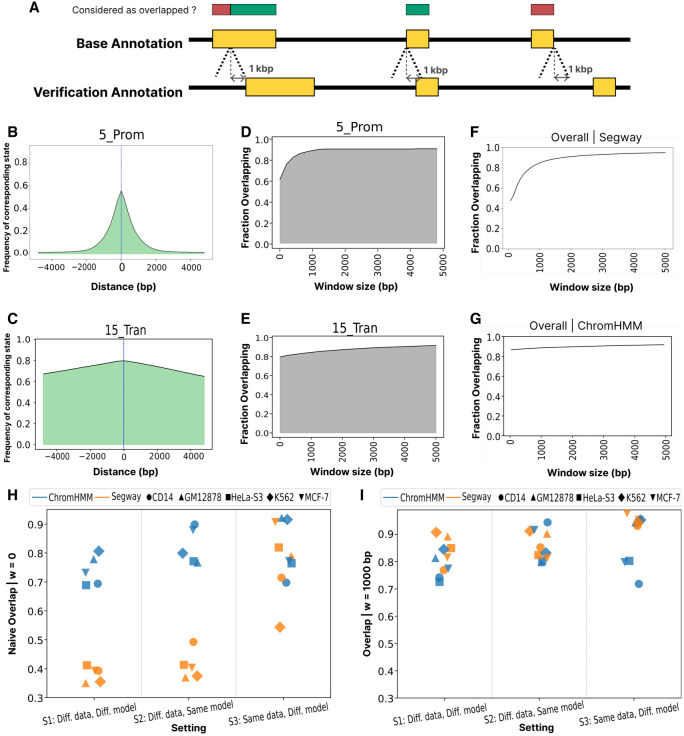
Spatial misalignment of corresponding chromatin states. (*A*) Schematic depicting the types of state misalignment and the influence of the tolerance window *w*. The horizontal axis depicts a genomic window. Yellow rectangles indicate a base state and its corresponding verification state. Red/green rectangles indicate whether the given position in the base annotation would be counted as overlapping the verification replicate. (*B*) Given that a position *g* is annotated as base state 5_Prom(B), the probability that a nearby position is annotated as the corresponding verification state 13_Prom_fla(V) as a function of distance from *g*. (*C*) Same as *B*, but for 15_Trans(B). (*D*) Given that a position *g* is annotated with base state 5_Prom(B), the probability (vertical axis) that the corresponding label occurs within a window *g* ± *w* as a function of *w* (horizontal axis). (*E*) Same as *D*, but for 15_Trans. (*F*,*G*) The overall overlap of the base annotation from Segway and ChromHMM, respectively, as a function of *w* (GM12878, setting 1). (*H*) Naive overlap across two SAGA models, five cell types, and three settings. Color denotes the SAGA model, and shape represents cell type. (*I*) Same as *H*, but allowing for a spatial tolerance window of *w* = 1000.

We found that a substantial fraction of mismatch between replicates results from spatial misalignment of segment boundaries. This may occur when two segments with corresponding chromatin states from base and verification annotations partially overlap but their borders do not fully align, or when two nonoverlapping segments with corresponding states occur in close proximity (Fig. 3A). Such imprecision in segment boundaries may be unimportant to practitioners because it may not meaningfully hinder downstream applications, such as in identifying putative regulatory elements or localizing disease-associated variation.

Therefore, to evaluate the reproducibility of annotations, we investigated the overlap between analogous chromatin states while considering a window of size *w* = 1000 bp upstream of and downstream from any given genomic positions. In other word, in this refined definition of overlap, each annotation at position *g* in base annotation is considered overlapped if its corresponding state is observed within the window of *g* ± *w* in the verification annotation.

We found that a large fraction of irreproducibility in SAGA annotations can be attributed to spatial misalignment (Fig. 3). Corresponding states frequently occur upstream of and downstream from a target position (Fig. 3B,C). When measuring overlap while considering a window around each position, we observe that for the majority of chromatin state types, a window of size *w* = 1000 bp significantly increases the overlap by capturing most of the misalignments, especially for chromatin states with short segments. For instance, in our running example, ∼60% regions labeled as 5_Prom(B) exactly overlap (*w* = 0) their corresponding state in verification annotation (Fig. 3D). However, by slightly increasing the window size, the fraction of overlapping regions increases to ∼90%. This implies that for ∼30% of positions that are labeled as 5_Prom(B) in base annotation, we can find a corresponding state in verification annotation within close proximity (1 kbp). However, states such as 15_Tran(B) are less affected by spatial misalignment (Fig. 3C,E; Supplemental Figs. 17–20).

Segway annotations are particularly susceptible to misalignment. Allowing for misalignment up to *w* = 1000 increases overlap in Segway annotations from 47% to 85% (Fig. 3F). In contrast, we do not observe a similar pattern for ChromHMM (Fig. 3G). This pattern occurs because Segway uses signal values rather than binarized data and thus is sensitive to variation in the scale of input signals across replicates. This can be attributed to hypersegmentation in Segway annotations owing to its sensitivity to variation in input signals (Hoffman et al. 2012; Chan et al. 2018). Segway also tends to hypersegment the genome into small segments that are inherently more prone to misalignment than are longer segments.

Notably, without accounting for misalignments, in settings S1 (different data, different models) and S2 (different data, same model), we can observe a distinct gap between ChromHMM and Segway in terms of overall overlap (Fig. 3H). However, S3 (same data, different models) essentially removes this gap and results in overall overlap values that are roughly in the same range for both ChromHMM and Segway. In S3 (same data, different models), we do not observe severe misalignment issues for Segway, which confirms Segway's sensitivity to noise in the input data. Moreover, ChromHMM's overall overlap is significantly less dependent on window size, suggesting less susceptibility to misalignment owing to data binarization and removal of fine details in the input data. After accounting for misalignment, Segway annotations significantly improve in terms of reproducibility and slightly outperform ChromHMM annotations (Fig. 3I).

### Posterior probability indicates robustness of annotations

SAGA methods generate posterior probabilities that indicate the model's confidence about the state assignment at every genomic position. We hypothesized that these posterior probabilities indicate reliability, such that confidently annotated positions are robust. However, these probability values are often vastly too confident; most positions receive >0.99 posterior probability (Libbrecht et al. 2021).

We found that although rates of reproducibility are much lower than model posterior probabilities, there is a strong increasing relationship between posterior probability and reproducibility. Specifically, for each chromatin state in the base annotation, we evaluated the frequency with which it overlaps its corresponding state in verification annotation as a function of the model's posterior probability, using an isotonic regression (Methods). For Segway, doing this analysis required modifying the underlying model to artificially weaken model predictions to avoid posteriors being rounded up to exactly 1.0 or down to zero owing to floating point under/overflow (Supplemental Sec. 1).

**Figure 4.**
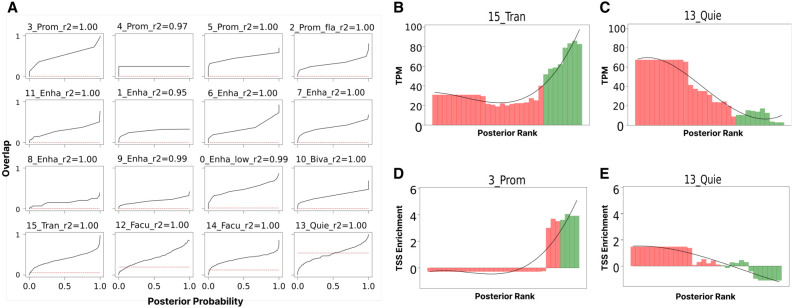
Posterior probability is associated with reproducibility. (*A*) The frequency of overlap with a corresponding state as a function of posterior probability for all states in the base annotation in the running example (ChromHMM, GM12878, S1). In each subpanel of *A*, the horizontal axis corresponds to the posterior probability, and the vertical axis corresponds to the observed overlap. The red dotted horizontal line in each subpanel represents the genome coverage of the corresponding state in the verification state, indicating the overlap expected from random overlap. The *R*^2^ score represents how well the isotonic regression curve fits the data. (*B*) The mean expression level (TPM) as a function of the posterior of 15_Tran(B). The horizontal axis represents rank of posterior probability, and the vertical axis shows TPM. (*C*) Same as *B*, but for 13_Quie(B). Similarly, *D* shows the enrichment around TSS regions as a function of the posterior of a 3_Prom(B). The horizontal axis shows the rank of posterior probability, and the vertical axis shows enrichment around TSS. (*E*) Same as *D*, but for 13_Quie(B); confirming that the posterior contains biologically relevant information about transcription start sites. In *B*–*E*, green bars represent genomic loci for which the maximum-a-posterior is the target chromatin state in the base annotation, and red bars correspond to the loci where the maximum-a-posterior is another chromatin state.

The results showed a positive trend between posterior probability and reproducibility, indicating that higher posterior probabilities are associated with higher levels of reproducibility (Fig. 4A; Supplemental Figs. 21, 22). This means that despite being overconfident, posterior probability of SAGA methods contain information about their reproducibility.

Posterior probabilities also contain biologically relevant information about gene expression and transcription start sites (TSSs), further confirming their usefulness. We found that there is a strong correlation between posterior probability of the transcribed state within a gene body and expression of that gene (Fig. 4B); that is, as the posterior of transcribed chromatin state increases, the average expression as measured by RNA-seq increases as well. Conversely, the posterior of the quiescent state is negatively correlated with transcription (Fig. 4C; Supplemental Figs. 23–26). Notably, even for positions that did not ultimately receive a label of quiescent, a small but nonzero posterior probability of quiescent at the gene body is associated with low expression.

Similarly to the result on gene bodies, posterior of promoter states is positively correlated with occurrence of annotated TSSs, and the converse is true of quiescent states (Fig. 4D,E; Supplemental Figs. 27, 28).

We found that the posterior of the state assignment to the base annotation conveys a great deal of information about what state is assigned to the verification annotation (Methods). As mentioned above, knowing only the identity of the state assigned to the base annotation removes 51% of the uncertainty in the verification state, as measured by the mutual information/entropy ratio (Fig. 2H). However, knowing both the state and its posterior probability increases the mutual information to 85% of base annotation's entropy, suggesting that the posterior probability strongly indicates whether the annotation of a given genomic bin is reliable. This increase is even more dramatic for Segway, increasing the percentage of the base annotation's entropy explained by mutual information from 20% − 35% to 64% − 72% (Fig. 2I).

### SAGAconf yields robust annotations

As described above, even when allowing for variability in granularity of state definitions and in spatial positioning, the reproducibility of SAGA annotations falls below the 90%–95% threshold sought in most applications. As described in the previous section, the posterior probability output by the SAGA probabilistic model indicates the reliability of the annotation of each genomic position.

To produce reliable chromatin state annotations, we propose SAGAconf. SAGAconf infers the probability that the annotation to a given genomic position will be reproduced in a replicate experiment (within a spatial tolerance of *w* = 1000 bp), according to the SAGA model's output state and posterior probability, which we term the *r*-value (Methods). SAGAconf outputs the annotation to a subset of the genome passing a user-defined *r*-value threshold (usually 0.9 or 0.95). Thus, SAGAconf is analogous to the IDR pipeline used for ChIP-seq peak calling and related tasks (Li et al. 2011). SAGAconf is independent of SAGA methodology, as it takes as input only a SAGA annotation and its posterior probability matrix.

To avoid ambiguity, we use different notation for two types of fractions. First, we use percentages (e.g., “85%”) to denote the observed genome-wide overlap of any two states, that is, the fraction of positions that have the corresponding state in the verification annotation. Second, we use decimals (e.g., “0.85”) to denote the fraction of confidently annotated regions that are derived from setting a threshold on the predicted overlap, that is, the *r*-value.

**Figure 5.**
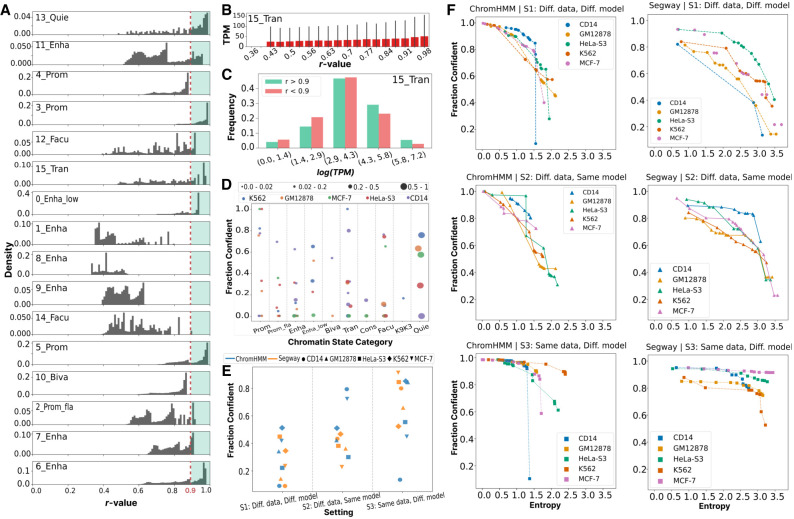
SAGAconf identifies confident state annotations. SAGAconf integrates sources of reproducibility information such as granularity, alignment, and posterior to derive an *r*-value for each genomic position. The *r*-value estimates the probability of reproducing the annotation at a specific genomic position. (*A*) Density histogram of *r*-values for each chromatin state in the running example (S1, ChromHMM, GM12878). A threshold of α = 0.9 is applied to label positions with *r* ≥ α as reproducible, or they are irreproducible otherwise. The horizontal axis represents the *r*-value; the vertical axis represents density; the red dotted line represents the threshold; and the green-shaded area show confident annotations. (*B*) Bar plot representation of expression level (TPM) as a function of *r*-value for transcribed chromatin states in the running example. Each bar signifies the mean TPM for each *r*-value bin, with error bars indicating the standard deviation. (*C*) Histogram comparing the frequencies of log(TPM) values for confident (*r* ≥ 0.9) and nonconfident (*r* < 0.9) annotations of the transcribed chromatin state in the running example. The *x*-axis represents ranges of log(TPM) with each pair of bars corresponding to confident (green) and nonconfident (red) annotations, respectively. (*D*) Fraction of chromatin states called confident by SAGAconf for ChromHMM annotations according to S1 (different data, different models) in five cell types. Each dot represents a chromatin state, with color denoting cell type and size proportional to genome coverage. (*E*) Fraction of genome called confident across two SAGA models, five cell types, and three settings. Color denotes SAGA model, and shapes represent cell types. (*F*) We measured the fraction of the genome called as confident by SAGAconf during the process of postclustering; that is, we measured the fraction of confident positions in the genome as a function of the base annotation's entropy as we merged similar chromatin states in the base annotation. The horizontal axis represents the base annotation entropy; the vertical axis represents the fraction of genome identified as confident; subpanels correspond to different SAGA models and settings; and colors represent cell types.

We found that *r*-values are distributed differently across chromatin states (Fig. 5A). In our running example, nearly all positions labeled with 3_Prom(B) achieve *r* > 0.9, meaning that this state is reproduced independent of the model's posterior. Conversely, all positions with state 4_Prom(B) have *r* < 0.9, indicating that this state can never be confidently annotated. However, most states show a range of *r*-values, indicating that the reliability depends on the posterior; for example, the rate of 0.52 of state 0_Enha_low(B) indicates a sufficiently high posterior to pass the *r* > 0.9 threshold. Thus, applying SAGAconf is critical to knowing whether the annotation to any given locus can be relied upon.

We found that confident labels are more likely to be truly active. For example, the *r*-value with which a gene is annotated as transcribed is strongly correlated with that gene's expression (Fig. 5B,C). Also, most highly expressed genes are confidently marked as transcribed (*r* > 0.9).

Overall, 0.10 − 0.50 of positions are annotated confidently according to SAGAconf (Fig. 5E). Most enhancer states from ChromHMM are irreproducible (Fig. 5D), whereas Segway identifies more-reproducible enhancers (Supplemental Figs. 29–37). After applying SAGAconf, Segway and ChromHMM both show roughly similar reproducibility in terms of fraction of the genome that is identified as confident (Fig. 5E).

Broad inactive chromatin states, such as quiescent states, are generally more reproducible compared with more punctate active elements like promoters and enhancers. This is partly because, unlike quiescent-like states that are associated with a depletion of all histone marks, active regulatory elements are identified by a combination of multiple signals and are therefore more sensitive to noise. Moreover, unlike naive overlap, which tends to decrease around segment boundaries for most chromatin states, the *r*-value is less affected by segment length and position relative to the segment boundaries. This issue is addressed by including a tolerance window for misalignment (Supplemental Figs. 35, 36). Applying SAGAconf restricted only to previously identified elements (The ENCODE Project Consortium et al. 2020; Meuleman et al. 2020) yields slightly more optimistic confidence estimates but has the disadvantage of ignoring a large fraction of the genome (Supplemental Fig. 37).

SAGA models with fewer states can confidently annotate a much larger fraction of the genome. In our running example, the default 16-label annotation has about 2 bits of entropy and confidently annotates 0.43 of the genome, but merging down to an annotation with 1.7 bits (10 labels) yields 0.55 of them being confidently annotated. This difference is more extreme for Segway. Although the default 16-label annotation can confidently annotate only a small fraction (<0.10), a 1.7-bit annotation confidently annotates 0.64 of the genome (Fig. 5F; Supplemental Figs. 14–16).

## Discussion

Chromatin state annotations are essential for various downstream tasks, including identifying regulatory regions and cell type–specific activity patterns, interpreting disease-association studies, studying gene regulation, and analyzing cellular differentiation (The ENCODE Project Consortium 2012; Hoffman et al. 2013; Roadmap Epigenomics Consortium et al. 2015; Libbrecht et al. 2019, 2021). Therefore, it is paramount to ensure their reliability, else all subsequent analysis may be inaccurate. Yet, although statistical guarantees (such as *P*-values, *Q*-values, or IDR) are used ubiquitously in genomics, and in science in general, no such statistical guarantee previously existed for SAGA.

This study tackles a repeatedly encountered problem in the field that is the lack of comprehensive and principled approaches for evaluation of SAGA genome annotations. Conventionally, SAGA genome annotations are evaluated using qualitative and quantitative methods (Libbrecht et al. 2021). Qualitative methods involve examining various statistics of an annotation to determine whether it reflects expected features of genome biology (Buske et al. 2011). However, currently, there are no generally agreed upon statistics that hold for all high-quality annotations. Quantitative methods, on the other hand, involve prediction problems such as predicting RNA-seq expression given only the annotation label at the gene's promoter (Zhang and Hardison 2017; Libbrecht et al. 2019; Daneshpajouh et al. 2022), but such prediction tasks are useful for the purpose of comparing different annotations but do not serve as a realistic evaluation of the annotations themselves. This study introduces SAGAconf as a comprehensive and principled alternative for this task that addresses various shortcomings of previous evaluation approaches.

Here, we provide a comprehensive evaluation of genome annotations in terms of their reproducibility and confidence. Using replicated data, we delineate different sources of irreproducibility stemming from the data and the SAGA models. We found that SAGA chromatin state annotations are frequently irreplicable, meaning that they often disagree when run on two replicated data sets. A substantial fraction of this disagreement remains after accounting for mismatch in chromatin states across models and for spatial misalignment between segments. This finding suggests that there is significant risk to using SAGA annotations without any filtering.

To mitigate this risk, we introduce a framework, SAGAconf, that identifies a confident subset of the genome that is annotated reliably. SAGAconf does so by leveraging the posterior probability of the underlying probabilistic graphical model used by the SAGA method, which we showed to be informative of reproducibility. We showed that SAGAconf correctly distinguishes reliable versus unreliable SAGA annotations within the genome. Thus, downstream applications of SAGA annotations would be improved by applying SAGAconf to filter out genomic positions with unreliable labels. This filtering step is analogous to the use of IDR analysis for ChIP-seq peak calling. The IDR analysis takes two sets of *P*-values, compares them, and uses this comparison for mutual calibration. In this study, we aim to generalize the one-dimensional reproducibility analysis (as in ChIP-seq IDR) to multidimensional data (posterior probability of various states). Essentially, SAGA models output *k* genomic vectors, each representing the posterior probabilities of each genomic position being annotated as one of the *k* states. SAGAconf establishes a one-to-one correspondence between states in base and verification annotations. Following this, the procedure becomes analogous to IDR in the sense that it compares the reproducibility across these two one-dimensional vectors. SAGAconf also allows for defining a customized degree of tolerance to irreproducibilities that are likely inconsequential for downstream analysis. This makes the evaluation more practically suited to the task of chromatin state annotation.

Our study extends the concept of reproducibility from one-dimensional analysis, as seen in ChIP-seq, to multidimensional categorical data, capturing the complexity of chromatin state annotations. Measuring multidimensional reproducibility is challenging because a mismatch between states could, for example, derive from either a mismatch between promoter and enhancer or a mismatch between promoter and quiescent, but the latter is much more consequential than the former. To mitigate this issue, we perform a postclustering state-merging step that automatically merges states that are similar in terms of their overlap patterns. This approach tends to merge similar states (such as related types of regulatory elements) rather that dissimilar states. Moreover, because SAGA annotations are multidimensional and lack a ground truth, the traditional concepts of sensitivity and specificity are not directly applicable. We propose an alternative interpretation of sensitivity in terms of the granularity of chromatin states. This refers to our ability to distinguish between different functional elements. Additionally, by setting a predefined target level of confidence (α = 0.9), we introduce a measure of specificity. Using SAGAconf, one could then maximize sensitivity at this specified threshold, providing a balance between the two.

As an alternative to using overlap to identify corresponding states, one could use similarity of learned emission parameters; doing so yields similar results (Supplemental Fig. 7). Moreover, in the case in which a SAGA model does not output posterior probabilities, a measure such as Euclidean distance between expected and observed signals could be used as a surrogate. Although it is possible to integrate the impact of the epigenetic profile on specific biological processes, such as gene expression, during state matching, we chose not to do so for two reasons: (1) To facilitate the usability of SAGAconf, we designed the pipeline to rely solely on empirical pairwise overlap data, which can be extracted from annotations, and (2) we intended to use the information on gene expression and TSSs as independent biological validations for our method.

The *r*-value represents the probability of an annotation being reproduced in a biologically replicated experiment. In that sense, a uniform threshold α on the *r*-value is analogous to setting a fixed threshold on a *P*-value. A researcher may instead choose to change the threshold to retain a certain fraction of genomic positions, or to apply a different threshold to each state. However, this goes against the convention of using a fixed *P*-value or *Q*-value threshold.

SAGAconf technically allows for the comparison of two annotations derived from different sets of epigenetic features or even separate types of data entirely. Evaluating its utility for this task is an interesting area for future research. Such insights could guide upstream experiments, potentially leading to the selection of a smaller, fixed subset of epigenomic experiments sufficient for high-quality and reproducible annotations.

Several SAGA methods, including IDEAS (Zhang et al. 2016), Segway-GBR (Libbrecht et al. 2015), TreeHMM (Biesinger et al. 2013), and others (Libbrecht et al. 2021), use vertical sharing, in which the model shares position-specific information across samples in the annotation process. Running SAGA with imputed data (Ernst and Kellis 2015; Schreiber et al. 2020, 2023) is another type of vertical sharing. Vertical sharing has the potential to improve reproducibility because sample-specific experimental noise can be averaged away through sharing. Evaluating this improvement is an interesting area for future research.

Moreover, IDEAS shares the definitions of states across cell types, that is, horizontal sharing. We evaluated the effect of this type of sharing through setting 2 (different data, same model), which removes state inequivalencies as a source of variation by using a shared definition of chromatin states. However, significant irreproducibility persists despite shared state definition. The 2D nature of chromatin state calling in IDEAS, which encourages alignment across cell types, is also likely to improve annotations when information from both replicates is taken into account. To assess reproducibility in this case, one would need to perform the same task twice on two pairs of replicates and compare the results. However, data availability limits such analysis.

The reproducibility of chromatin states depends greatly on the quality of data; that is, estimations of confidence are affected by the replicate with inferior data quality. Thus, even if one replicate is good and the other is not, the overall reproducibility is deemed poor owing to the subpar replicate. In addition to obtaining better replicated data, and more-effective preprocessing methods (Landt et al. 2012; Xiang et al. 2020; Bayat and Libbrecht 2021), future endeavors can tackle this problem by designing SAGA models that are more robust to data quality.

Future studies should enhance our understanding by investigating the impact of the upstream decisions on the reproducibility of downstream annotations: for instance, the choice of SAGA methods (e.g., Segway [Hoffman et al. 2012], ChromHMM [Ernst and Kellis 2012], IDEAS [Zhang and Mahony 2019], or others), model hyperparameters, resolution (e.g., by using coarse resolution to annotate domains), and data types (e.g., conservation via ConsHMM) (Arneson et al. 2020). Additionally, factors such as data quality and the availability and selection of epigenomic assays should be considered. These measurements can be potentially valuable for inferring developmental and lineage-specific changes in the epigenome.

Given the rising applications of sequence-to-activity models (Zhou and Troyanskaya 2015; Kelley et al. 2016; 2018; Avsec et al. 2021), one could use SAGAconf to evaluate the reproducibility of their predictions. For instance, researchers could apply SAGA models to distill several predicted activities into chromatin state representations followed by applying SAGAconf to measure their reproducibility. This approach can be particularly useful in measuring the functional effects of in silico mutagenesis (ISM) (Novakovsky et al. 2023). Additionally, SAGAconf could help improve the training data for these models by identifying assays or genomic positions with high reproducibility, leading to training more accurate models.

Currently, SAGAconf treats the base and verification replicates separately. Combining these replicates to obtain a unified representation of reproducibility across experiments is a potential area for future research. A naive approach to combining replicates would be to run SAGAconf twice, with each of the two possible assignments to base and verification, and then output the intersection of the confident regions.

Many epigenomic assays on public databases such as The ENCODE Project Consortium (2012) are not performed on replicated experiments, so a version of SAGAconf that does not require replicated data would be valuable. One naive option would be to use data from a similar cell type as a verification replicate. Such a confidence estimate will be conservative, meaning that a smaller fraction of the genome will be deemed reproducible by SAGAconf. There is a need for future work to develop a model that can generalize statistics from replicated cell types to unreplicated cell types.

## Methods

### Data collection

We retrieved sets of replicated histone modification ChIP-seq data for five cell types from the ENCODE DCC (The ENCODE Project Consortium 2012; http://encodeproject.org/). These cell types are all among ENCODE's tier-1 and tier-2 cell types and have the greatest number of ChIP-seq assays with isogenic replicate data (Supplemental Sec. 1). Isogenic replication, also referred to as biological replication, is a process in which two biosamples are derived from the same human donor or model organism but are treated separately (https://www.encodeproject.org/data-standards/terms/).

Consequently, we collected pairs of replicated histone modification ChIP-seq data for the following cell types (Supplemental Table 2; Supplemental Figs. 38–42):

1. GM12878 with 11 histone modification assays,
2. K562 with 11 histone modification assays,
3. HeLa-S3 with 11 histone modification assays,
4. CD14-positive-monocyte with 11 histone modification assays, and
5. MCF-7 with 13 histone modification assays.

For each cell type, we assembled two data sets each consisting of all of the assays belonging to one replicate. These data sets consist of a feature vector for each genomic position in which each element corresponds to a particular histone modification measurement. With *d* histone modification assays, the data set is a ℝ^*G*×*d*^ matrix, in which *G* denotes genomic positions. For instance, for cell type GM12878, we have two replicated data sets, and in each data set, we have an 11-dimensional feature vector per genomic position. These feature vectors are considered by SAGA models as observed events, *X*_*g*_, and are used as training data. All of the ChIP-seq data are aligned with the hg38 (GRCh38) reference human genome.

### Model training and annotation

Using the collected data, we trained ChromHMM and Segway models with a fixed set of hyperparameters and obtained chromatin state annotations from them. Specifically, for both methods, to specify the number of chromatin states, we used the formula ($10+2\sqrt{\mathrm{number}\;\mathrm{of}\;\mathrm{assays}}$) as suggested by Libbrecht et al. (2019) to scale with the amount of available data (11 histone modification assays for GM12878, K562, HeLa-S3, and CD14-positive-monocyte and 13 assays for MCF-7). For specifying hyperparameter settings of both ChromHMM and Segway, we followed the standard practice as previously performed (Ernst and Kellis 2012; Hoffman et al. 2012, 2013; Libbrecht et al. 2015, 2019). Details of hyperparameter settings that were used for model training and annotation are explained in Supplemental Section 1 (Supplemental Table 1).

As a postprocessing step, to enable comparison between two replicates, we divided the genome into resolution-sized positions and assigned the posterior *P*(*Q*_*g*_ = *q*|*X*) and annotation results to each genomic position. This additional parsing step provides us with a unified format that is readily comparable, independent of data sets, parameters, or even the SAGA algorithm that generated the annotations. Parsed annotation results are matrices of size *G* × *K*, where *K* denotes the number of chromatin states, and *G* corresponds to the number of genomic positions; that is, the whole genome size is divided by ‐‐resolution, and each element in the matrix corresponds to the posterior probability of each state at each position.

### Biological state interpretation

As the raw annotation results of SAGA algorithms are states named with the integer ID of their clusters, we need an additional interpretation step in which cluster IDs are translated to obtain human-readable results into functional biological roles (also known as “mnemonics”). To avoid bias from manual interpretation, we used an automated interpretation procedure introduced by Libbrecht et al. (2019). Specifically, we used a pretrained random forest classifier that, using the enrichment of states around conserved regions and enrichment of different histone modification marks for each state, assigns a predicted biological interpretation to each state. This classifier is trained on Segway annotations and might not be as accurate on ChromHMM annotations. These interpretation terms are solely for the interpretability of results, and every step of the reproducibility evaluation pipeline is independent of the interpretation terms (Farahbod et al. 2023).

### Pairwise overlap of chromatin states

SAGA methods output integer-state IDs for each identified chromatin state. Because they are unsupervised, the state IDs are not consistent across two different models. Therefore, to enable comparison among chromatin states of the two annotations, the first step is to identify states that correspond to related genomic functions across the two annotations by measuring the pairwise overlap between them.

Let *k* be a chromatin state in the base annotation *B*, and let *l* be a chromatin state in verification annotation *V*. Their joint frequency of overlap $\hat{P}(B=k,V=l)$ is defined as

(1) $$\hat{P}(B=k,V=l)=\frac{1}{G}\overset{G}{\underset{g=1}{\sum }}1(q_{g}^{B}=k,q_{g}^{V}=l),$$

where *g* corresponds to genomic positions, and $q_{g}^{B}$ and $q_{g}^{V}$ denote assigned states to the genomic position *g* in base and verification annotations, respectively. Similarly,

(2) $$\hat{P}(B=k)=\frac{1}{G}\overset{G}{\underset{g=1}{\sum }}1(q_{g}^{B}=k)$$

is the marginal frequency (i.e., genomic coverage) of state *k* in the base annotation *B*.

The joint distribution of overlap $\hat{P}(B=k,V=l)$ is greatly influenced by the states’ genome coverage. For example, pairs of quiescent states that cover most of the genome will have a very high probability, whereas highly corresponding promoter pairs might get small overlap probabilities. To identify correspondence of states regardless of their genome coverage, we use IoU, also known as Jaccard's similarity coefficient, which is formulated as follows:

(3) $$\mathrm{IoU}(B=k,\;V=l)=\frac{\hat{P}(B=k,V=l)}{\hat{P}(B=k)+\hat{P}(V=l)-\hat{P}(B=k,V=l)}.$$

### Mutual information between annotations

The mutual information of base and verification annotations *I*(*B*;*V*) can quantify the shared information of two annotations, and it is calculated as follows:

(4) $$I(B;V)=\underset{k\in B}{\sum }\underset{l\in V}{\sum }\hat{P}(B=k,V=l)\log_{2}\left(\frac{\hat{P}(B=k,V=l)}{\hat{P}(B=k)\hat{P}(V=l)}\right).$$

Mutual information can have values ranging from zero for total independence and +∞. However, MI *I*(*B*;*V*) is always upper bounded by both entropies of the base annotation *H*(*B*) and the verification annotation *H*(*V*), which quantify the total amount information that can be contained within these annotations. Entropy of each set of annotations is calculated as follows:

(5) $$H(B)=-\underset{k\in B}{\sum }\hat{P}(B=k)\log_{2}\hat{P}(B=k).$$

Therefore, we normalize the mutual information into values that reflect the fraction of information in the base annotation that is shared with the verification annotation.

The posterior of the base replicate provides information into the replicability of the annotation. We compute the information as follows. We make 10 equally distanced bins based on the posterior values of the base annotation chromatin states. Let *i* be a bin index, where 1 ≤ *i* ≤ 10; the mutual information between the base and verification annotations *I*(*B*;*V*) with respect to posterior probabilities of the base annotation is calculated as follows:

(6) $$I(B;V)=\overset{10}{\underset{i=1}{\sum }}\underset{k\in B}{\sum }\underset{l\in V}{\sum }\hat{P}(B=k_{i},V=l)\log_{2}\left(\frac{\hat{P}(B=k_{i},V=l)}{\hat{P}(B=k_{i})\hat{P}(V=l)}\right).$$

### Overlap and spatial alignment

We hypothesized that because of a variety of noises, corresponding chromatin states might not locate at the exact same position across the two annotations. To investigate the impact of misalignment of corresponding segments, we relaxed the criteria for considering an annotation as overlapped by allowing a window of size *w* upstream of and downstream from any given position *g* to look for corresponding states. Therefore, we consider proximal positions in the overlap calculations:

(7) $$\hat{P}(B=k,V=l)=\frac{1}{G}\overset{G}{\underset{g=1}{\sum }}1(q_{g}^{1}=k,q_{g\pm w}^{2}=l),$$

where $\hat{P}(B=k,V=l)$ is the updated probability of overlap that considers a window of size *w* around segments of the verification annotation.

### Granularity of genomic functions

In a perfect case of reproducibility, one would expect that for each state *k* in base annotation, one state should exist in the verification annotation that exactly covers the same genomic positions. However, in practice, positions with state *k* in *B* are distributed over two or more states in *V* and vice versa.

To understand the effect of genomic functional granularity (i.e., the number of chromatin states) on reproducibility, we measure the overlap while iteratively merging verification states. To that end, for a given target state of base annotation, we sorted all of the states of verification annotation according to their IoU of overlap. Based on this order, we start merging the states in *V* iteratively until all of the genome is covered with one “superstate” in *V*. We evaluate what fraction of the genome needs to be covered by *V* states in order to cover most of the positions of the target state in *B*. We term this the “state-merging curve” (SMC).

The area under the SMC, in cases of perfect reproducibility, is when the first *V* state covers all of the positions of the target state in *B*. We define the ratio between these two curves as the area under the SMC (auSMC), which is a simple numerical representation of the state's reproducibility as a function of its chromatin state granularity.

### Calibration of posterior probability

The posterior probabilities produced by SAGA tend to be overly confident, with the majority of positions receiving a probability *>*99%, which does not accurately reflect the reliability of the annotations. However, we investigated if there is any correlation between reproducibility and posterior probability. By examining the relationship between posterior probability and reproducibility, as measured by pairwise overlap, we can improve confidence estimates by creating a calibration curve. This allows us to transform raw posteriors into more accurate and robust confidence scores that better represent the actual likelihood of reproducibility for a given annotation.

Initially, we establish pairs of corresponding chromatin states. For each state *k* in *B*, the state with the greatest IoU overlap score in *V* is considered as the matching state. To perform calibration, we computed the ratio of overlap as a function of the model's posterior. Specifically, let $q_{1\ldots G}^{\left(B\right)}\in {\left\{1..k\right\}}^{G}$ and $q_{1\ldots G}^{\left(V\right)}\in {\left\{1..l\right\}}^{G}$ represent vectors of base and verification annotations, respectively. In the base annotation, for each state *k*, we first arrange the vector of posterior probabilities of all genomic positions $p_{1\ldots G}^{k}\in R^{G}$ based on the posterior value. Then, we divide the sorted array into b subarrays (bins) of equal size such that *b*_*i*_ ⊂ {1…*G*}. For each bin, we compute the fraction of the target state in the base annotation that overlaps with its corresponding state in the verification annotation. It is expected that for first bins with lower posterior value, the overlap is lower than that of high posterior bins. By performing the binning step, we can quantitatively compare two variables with different natures, namely, the overlap and posterior probability.

We assume that the pairwise agreement and posterior probability of corresponding chromatin states are not negatively correlated. Thus, assuming a monotonic and nondecreasing trend, we fit an isotonic regression model to create a calibration curve (De Leeuw 1977; Chakravarti 1989; De Leeuw et al. 2010). Isotonic regression is a nonnegative piece-wise regression model in which we aim to learn a curve, $\hat{y}$, to solve a problem formulated as follows:

(8) $$\min\overset{N}{\underset{i=1}{\sum }}{\left({\hat{y}}_{i}-y_{i}\right)}^{2},\;\mathrm{subject}\;\mathrm{to}\;{\hat{y}}_{i}\leq {\hat{y}}_{j}\;\mathrm{whenever}\;b_{i}\leq b_{j}.$$

To further explain, the model attempts to fit a curve, $\hat{y}$, to the sequence of “posterior-versus-overlap ratio” bins *b* such that for all *b*_*i*_ ≤ *b*_*j*_, the curve ${\hat{y}}_{i}\leq {\hat{y}}_{j}$ will be produced, resulting in a nonnegative trend. We selected the isotonic regression model for this calibration task because, unlike linear regressions that impose linearity, these models are not limited by any functional form and can fit any form in the observed data as long as they are monotonically increasing. The pool-adjacent-violators algorithm (PAVA) is commonly used to fit the isotonic regression model (De Leeuw 1977; Chakravarti 1989; De Leeuw et al. 2010). We used isotonic regression from Python's Scikit-learn package (Pedregosa et al. 2011).

### Validation against known phenomena

In contrast to supervised learning problems, in which the predictions of models can be validated against a labeled test set, chromatin states lack a gold standard. One common method to validate annotations is to analyze the enrichment of promoter-like states around conserved positions such as TSSs from reference annotations (O'Leary et al. 2016; Ge et al. 2019; Libbrecht et al. 2019; Zhang and Mahony 2019). Another method is to check if the transcribed regions predicted by SAGA models overlap with experimentally validated expressed regions obtained from RNA-seq data (Daneshpajouh et al. 2022).

We used this approach to evaluate whether the posterior probability of annotations can confidently predict known regulatory regions. First, similar to calibration of posterior probability, for each state *k*, we rank the posterior probabilities $p_{1\ldots G}^{k}\in R^{G}$ in ascending order. Then, we split the sorted array into *b* equal size subarrays (bins) such that *b*_*i*_ ⊂ {1…*G*}. For each bin of posterior probability, we calculate the enrichment of regions with the posterior of state *k* in this bin range around TSSs. Enrichment is calculated as $\log\left(\frac{\mathrm{observed}}{\mathrm{expected}}\right)$. In this case, observed is the number of regions with the posterior of state *k* in a given bin range around TSS, and expected is the number of positions with the posterior of state *k* in that bin range across the whole genome. Similarly, for each bin, we investigate the mean RNA-seq expression level (transcripts per million [TPM]) of genomic positions within that posterior range (Wagner et al. 2012).

### Merging chromatin state to produce lower-granularity annotations

Determining the optimal number of chromatin states to specify as a hyperparameter to the SAGA model is not straightforward. However, it is evident that increasing the number of chromatin states (i.e., increasing granularity) leads to decreased reproducibility, as it becomes more difficult for the model to distinguish between different states. In other words, more granularity in the annotations leads to higher entropy, which naturally leads to irreproducibility. This presents a trade-off between the granularity of chromatin states learned from the data and the reproducibility of those annotations.

To investigate this trade-off, we iteratively merge pairs of the most-similar chromatin states in both the base and validation annotations. To do this, we need to define a metric *S* that measures the pairwise similarity of different chromatin states within one annotation based on their overlap behavior with the other annotation. We measure this pairwise similarity as follows:

(9) $$S_{k,k^{\prime }}=1-\frac{\Delta I(B;V)}{\Delta H(B)},$$

where Δ*I*(*B*;*V*) and Δ*H*(*B*) represent the change in mutual information and entropy of the base annotation, respectively, resulting from merging states *k* and *k*′ in the base annotation. The verification annotation goes through the exact same process at each iteration. Because posterior probabilities of states in an annotation are mutually exclusive, posterior probability of a merged state is the sum of posteriors of the two original states.

(10) $$P(k\cup k^{\prime }∥\theta ,X)=P(k∥\theta ,X)+P(k^{\prime }∥\theta ,X).$$

After calculating metric *S* for both the base and validation annotations, at each iteration we merge two pairs with maximum *S* as the most similar pairs for which merging results in the least change in mutual information and the greatest change in entropy. By merging any two chromatin states, both entropy and mutual information decrease; however, merging similar chromatin states (with a similar pattern of overlap) should ideally result in a minimum change in mutual information. This process is repeated until all pairs of chromatin states have been considered for merging. By analyzing how the mutual information and entropy change as pairs of chromatin states are merged, we can determine the optimal number of chromatin states that balances granularity and reproducibility.

### Delineating different sources of variability

To obtain a comprehensive insight into how the variability of models and data can affect reproducibility, we assessed the reproducibility of annotations in several settings and different levels of technical and biological variability. Therefore, we selected pairs of experiments in the three following scenarios to evaluate their reproducibility.

#### Setting 1 (different data, different model)

We trained two separate SAGA models using the collected data from each isogenic replicate. Two trained models were then used to annotate the genome. Finally, the annotations were compared to uncover the reproducibility from various sources, including the data, the model training process, parameter initialization, etc.

#### Setting 2 (different data, same model)

We concatenate data from both isogenic replicates to form a single extended data set. This data set is used to train a single SAGA model, which provides separate annotations for each of the replicates. The model contains one set of state definitions used for both annotations, making the matching step (calling corresponding states) unnecessary. This approach removes elements of variability in training and state matching, highlighting the irreproducibility that stems solely from the input data rather than from the model training or random state initialization. It allows us to uncover the irreproducible elements that are only attributed to the data from replicated experiments.

#### Setting 3 (same data, different model)

We also used the same data set (from base replicate) to separately train two different models while only changing the random seed used for parameter initialization. This setting uncovers the irreproducibility that is to be attributed to the initialization and training of models.

### SAGAconf

Our analysis suggests that there are various aspects to the problem of reproducibility of SAGA annotations, including the granularity of chromatin states, proximity and spatial alignment, and the information embedded within the models’ posterior probability. Therefore, we designed SAGAconf, an integrative approach that combines these sources of information to derive a reproducibility score (*r*-value) at each genomic position, which can then be used to filter out irreproducible state assignments and thus obtain robust chromatin state annotations.

The SAGAconf reproducibility assessment pipeline starts by defining corresponding states across the two annotations. We compute a IoU overlap matrix while considering a window of size *w* upstream of and downstream from each position according to Equations 3 and 7. Then, for each chromatin state in the base annotation, its corresponding state is identified as the state with which it has the maximum IoU among all verification annotation states.

Then, we calibrate posterior values into reproducibility scores. Here, at each bin *b*_*i*_ ⊂ {1…*G*}, we calculate the ratio of genomic position in base annotation within *b*_*i*_ that has the corresponding state in a window of size *w* around that position in verification annotation. Using the isotonic regression obtained from the last step, we get a reproducibility score for each position in the genome. The resulting reproducibility score *r*-value ranges from zero to one, representing the probability of that position or its proximity being labeled with a related genomic function in the other annotation.

Lastly, using a hard threshold *α* on the *r*-value, we assign a Boolean label of “reproduced” for *r* ≥ *α* or “not-reproduced” for *r* < *α* to every position in the genome. Using these Boolean labels of reproducibility, we can robustly identify a confident and reliable subset from genome annotation while removing the irreproducible predictions.

### Software availability

The code used to generate the results presented in this study is available at GitHub (https://github.com/mehdiforoozandeh/SAGAconf) and as Supplemental Code. Extended results and detailed information about the data sets used in this study can be found in the Supplemental Material.

## Supplementary Material

Supplement 1

Supplement 2
