# Supplementary figures and images for: Robust chromatin state annotation
Source: Genome Res. 2024 Mar;34(3):469–83. doi: 10.1101/gr.278343.123 (PMC11067878; doi:10.1101/gr.278343.123)

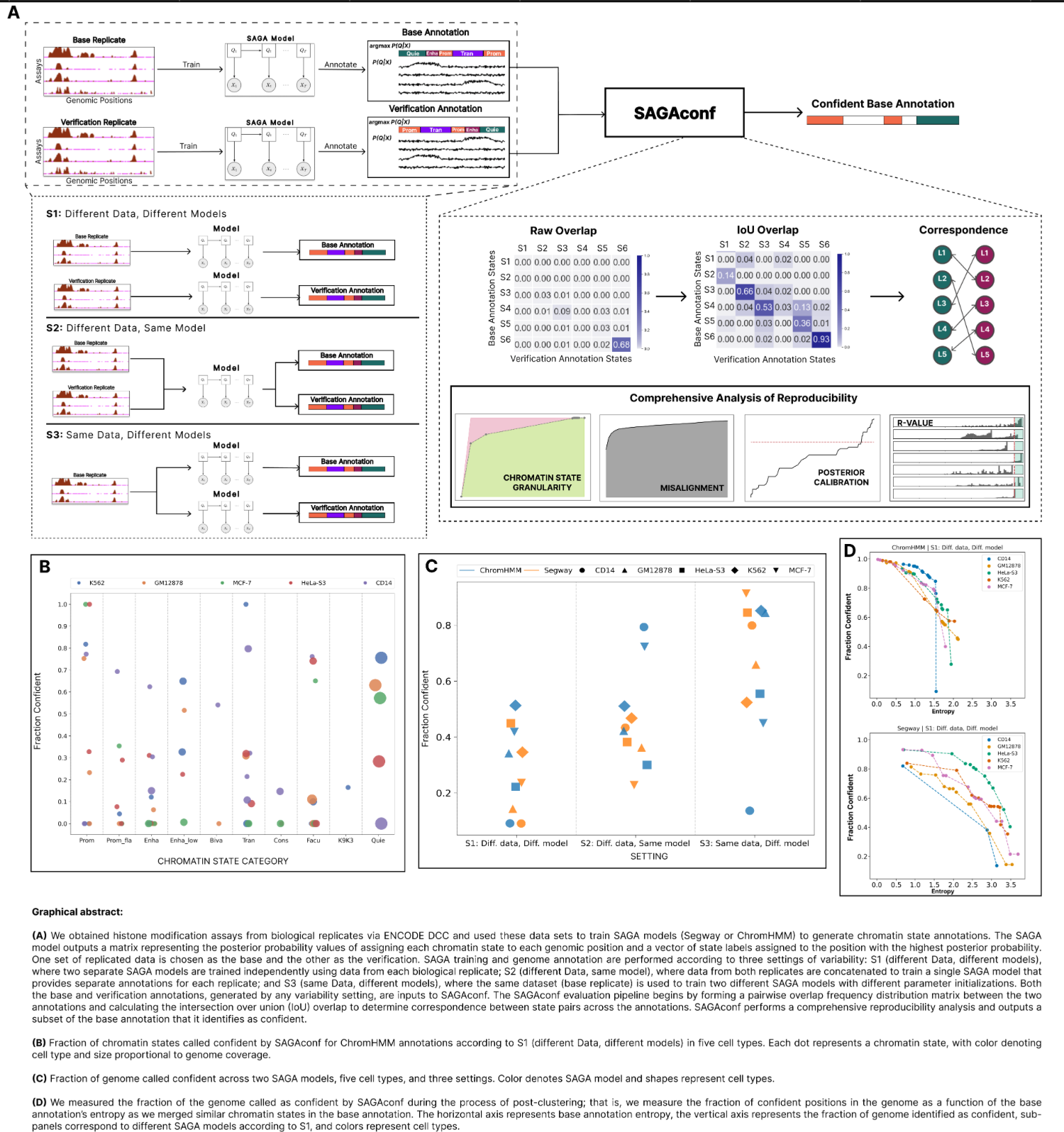

Supplement: Supplement 1 [file Supplemental_Code.zip › SAGAconf-main/Graphical_abstract.png]
