# Supplementary material for: Robust chromatin state annotation
Source: Genome Res. 2024 Mar;34(3):469–83. doi: 10.1101/gr.278343.123 (PMC11067878; doi:10.1101/gr.278343.123)
Supplement: Supplement 2 [file Supplemental_Document.pdf]

Robust chromatin state annotation

Mehdi Foroozandeh Shahraki, Marjan Farahbod, Maxwell Libbrecht *

1 Model training and annotation

1.1 Segway

Segway requires inputs to be loaded on a Genomedata file [Hoffman et al., 2010]. We loaded all the track files belonging to each replicate on a Genomedata file. As training Segway on whole-genome is computationally expensive, we trained the Segway model on mini-batches of 3 percent of the genome [Hoffman et al., 2013, Chan et al., 2018, Hoffman et al., 2012] (Table 1), and we obtained genome annotations and posterior probability values.

The hyper-parameter `--minibatch-fraction` specifies the fraction of the genome that is randomly selected by Segway at each training iteration and used for training [Chan et al., 2018].

With default parameter settings, Segway's posterior probabilities lack numerical precision due to underflow and overflow of floating points such that >90% of genomic positions receive either exactly zero or exactly one posterior value. To mitigate this issue and to enable downstream reproducibility analysis based on granular posterior probability values, we need to tune hyper-parameters. To tackle this issue and to obtain granular probability values, we artificially softened the model's posterior by raising the emission probabilities (`--track-weights`) to the power of $e = 0.01$ ($\frac{1}{\texttt{--resolution}}$) and by doing so, we uniformly decreased the model's confidence. The hyper-parameter `--track-weights` is the exponent of emission probability, and the modified posterior values are calculated as follows:

$$P(Q_{1,..,T} = q_{1,..,T} | X_{1,..,T}) = \prod_{t=1}^{T} P(Q_t = q_t | Q_{t-1}) P(X_t | Q_t = q_t)^e \quad (1)$$

Where, $P(X_t|Q_t = q_t)$ and $P(Q_t = q_t|Q_{t-1})$ are emission and transition probabilities, respectively, and 0.01 which is the exponent of emission probability is specified to the model through the hyper-parameter `--track-weights`. This additional step mitigates the issue of lack of granularity by increasing the number of positions with granular posterior value. Moreover, to specify the number of labels, we used the formula ($10 + 2\sqrt{\text{number of assays}}$) as suggested by [Libbrecht et al., 2019] to scale with the amount of available data. For all of our collected datasets with 10 to 13 histone modification assays, the number of labels is 16. Generally, having longer segments is desired for Segway [Hoffman et al., 2012, Chan et al., 2018] therefore, this method uses hard and soft constraints for controlling the length distribution of segments. The hyper-parameters `--prior-strength` and `--segtransition-weight-scale` are the soft constraints and act as length prior and exponent of transition probability, respectively. For Segway, we mostly followed the hyper-parameter settings of [Libbrecht et al., 2019].

**Hyper-parameter**	**Value**
`--resolution`	100
`--num-labels`	$10 + 2\sqrt{\text{number of assays}}$
`--track-weight`	$\frac{1}{100}$
`--segtransition-weight-scale`	1
`--prior-strength`	1
`--minibatch-fraction`	0.03

Table 1: Hyper-parameters used to train Segway models

1.2 ChromHMM

For ChromHMM we followed standard practice and trained the models with default hyper-parameter setting and on the whole genome. For ChromHMM runs, the `--resolution = 200` and the model is trained on whole genome. As a pre-processing step, ChromHMM binarizes the signals throughout the genome, using a hard threshold on the Poisson distribution probability [Ernst and Kellis, 2012], with each position having an indicator of the presence or absence of a certain epigenetic mark. Lastly, trained ChromHMM models provided genome annotations and their corresponding posterior probabilities.

1.3 Interpretation of Chromatin State Types

The table below provides an interpretation of chromatin state types in terms of their biological roles. These interpretations were generated using an automated method described in [Libbrecht et al., 2019], which predicts the biological interpretation of each label based on its enrichment around conserved regions and its association with different histone modification marks. It is important to note that these interpretation terms are provided for the purpose of making the results more interpretable, and all steps of the reproducibility evaluation pipeline are independent of these terms.

**Chromatin State Type**	**Short Name**	**Description**
Promoter	Prom	Presence of promoter-associated marks H3K4me3 and H3K9ac. Highly enriched at transcription start sites (TSSs).
PromoterFlanking	Prom_fla	Presence of promoter-associated marks H3K4me3 and H3K9ac, but at lower levels than Promoters. Tends to occur upstream or downstream of TSSs.
Enhancer	Enha	Presence of the enhancer-associated marks H3K27ac and H3K4me1.
EnhancerLow	Enha_low	Same as Enhancer, but with lower signal values.
Bivalent	Biva	Presence of both activating (H3K27ac) and repressive (H3K27me3) marks. Thought to mark regulatory elements “poised” for activation.
CTCF		Presence of the transcription factor CTCF, thought to play a role in chromatin conformation. In samples without measured CTCF, this label marks positions with CTCF-associated marks.
Transcribed	Tran	Characterized by the transcription-associated mark H3K36me3. Highly enriched in annotated gene bodies.
K9K36	K9K36	Presence of the marks H3K9me3 and H3K36me3, a pattern associated with zinc finger genes.
FacultativeHet	Facu	Facultative (Polycomb) Heterochromatin, characterized by the histone modification H3K27me3. Thought to carry out cell type-specific repression.
ConstitutiveHet	Cons	Constitutive Heterochromatin, characterized by the histone modification H3K9me3. Marks permanently silent regions such as centromeres and telomeres.
Quiescent	Quie	Lack of any marks

2 Comprehensive evaluation of chromatin state reproducibility

2.1 Setting 1: Different Data, Different Models

2.1.1 ChromHMM

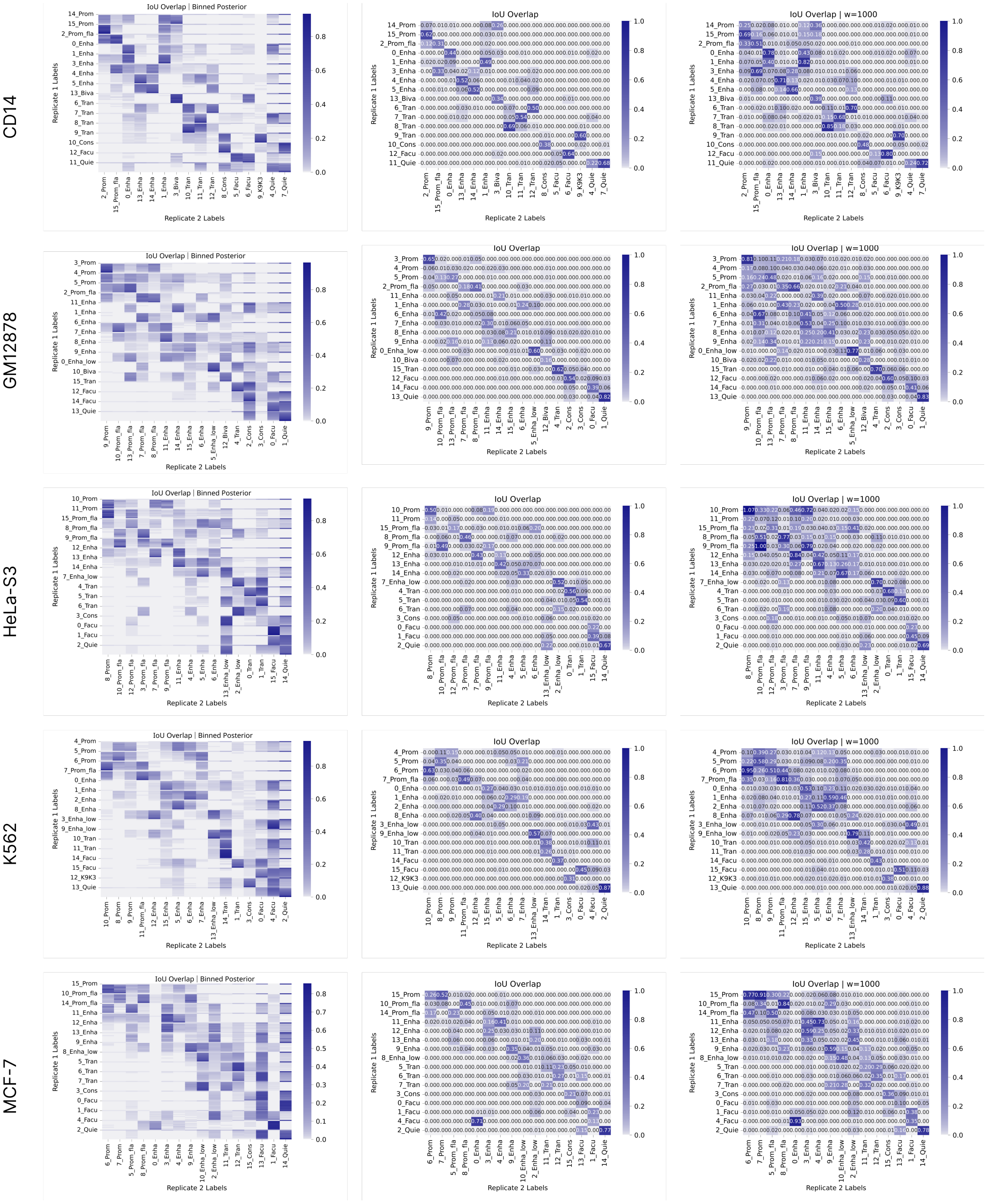

Figure 1: Intersection over union (IoU) overlap of ChromHMM according to setting 1 (different data, different models). Columns from left to right are binned posterior, IoU overlap with maximum-a-posterior state with $w = 0$, IoU overlap with maximum-a-posterior state with $w = 1000$. Color axis is linear and rows correspond to five cell types.

2.1.2 Segway

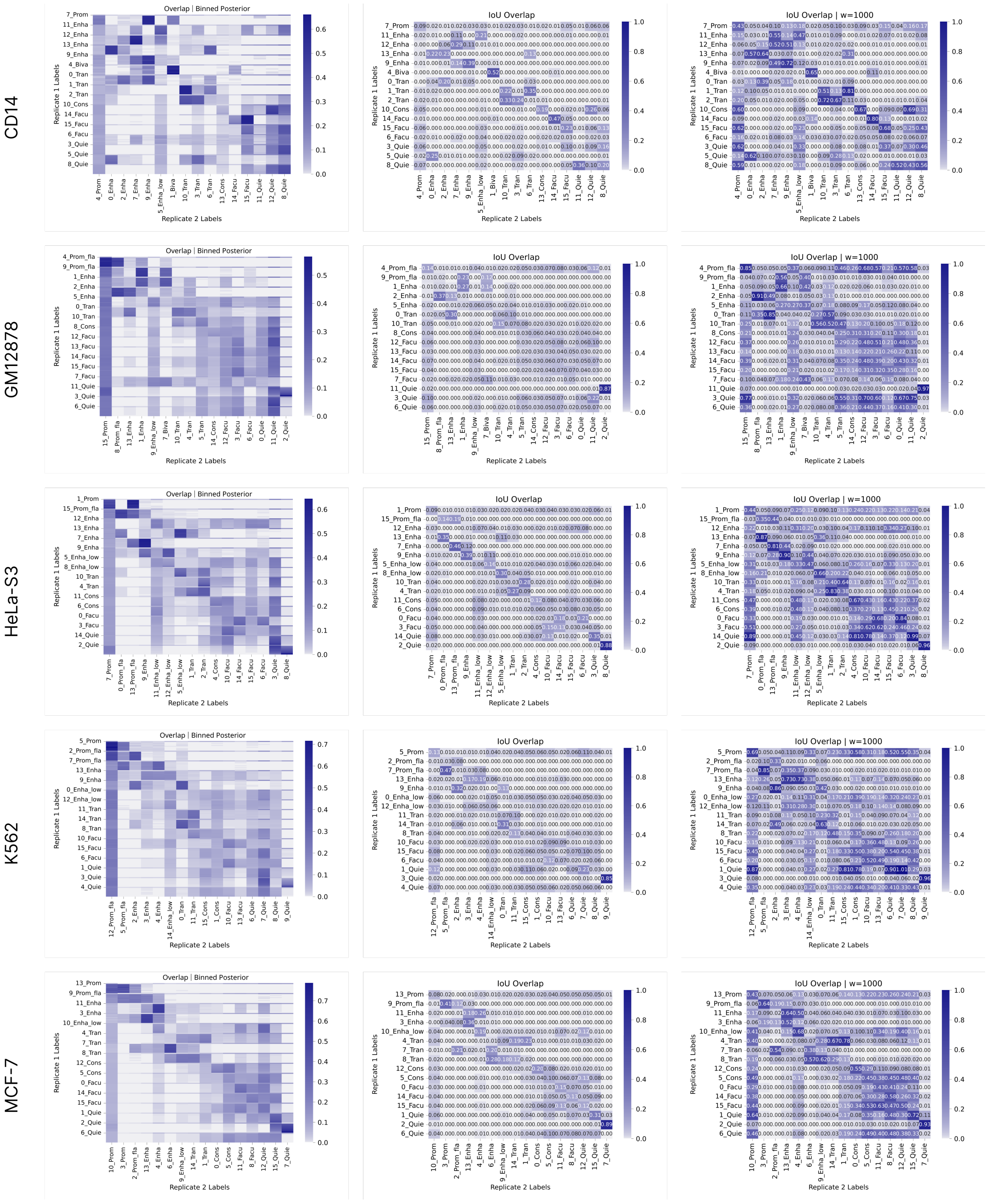

Figure 2: Intersection over union (IoU) overlap of Segway according to setting 1 (different data, different models). Columns from left to right are binned posterior, IoU overlap with maximum-a-posterior state with $w = 0$, IoU overlap with maximum-a-posterior state with $w = 1000$. Color axis is linear and rows correspond to five cell types.

2.2 Setting 2: Different Data, Same Model

2.2.1 ChromHMM

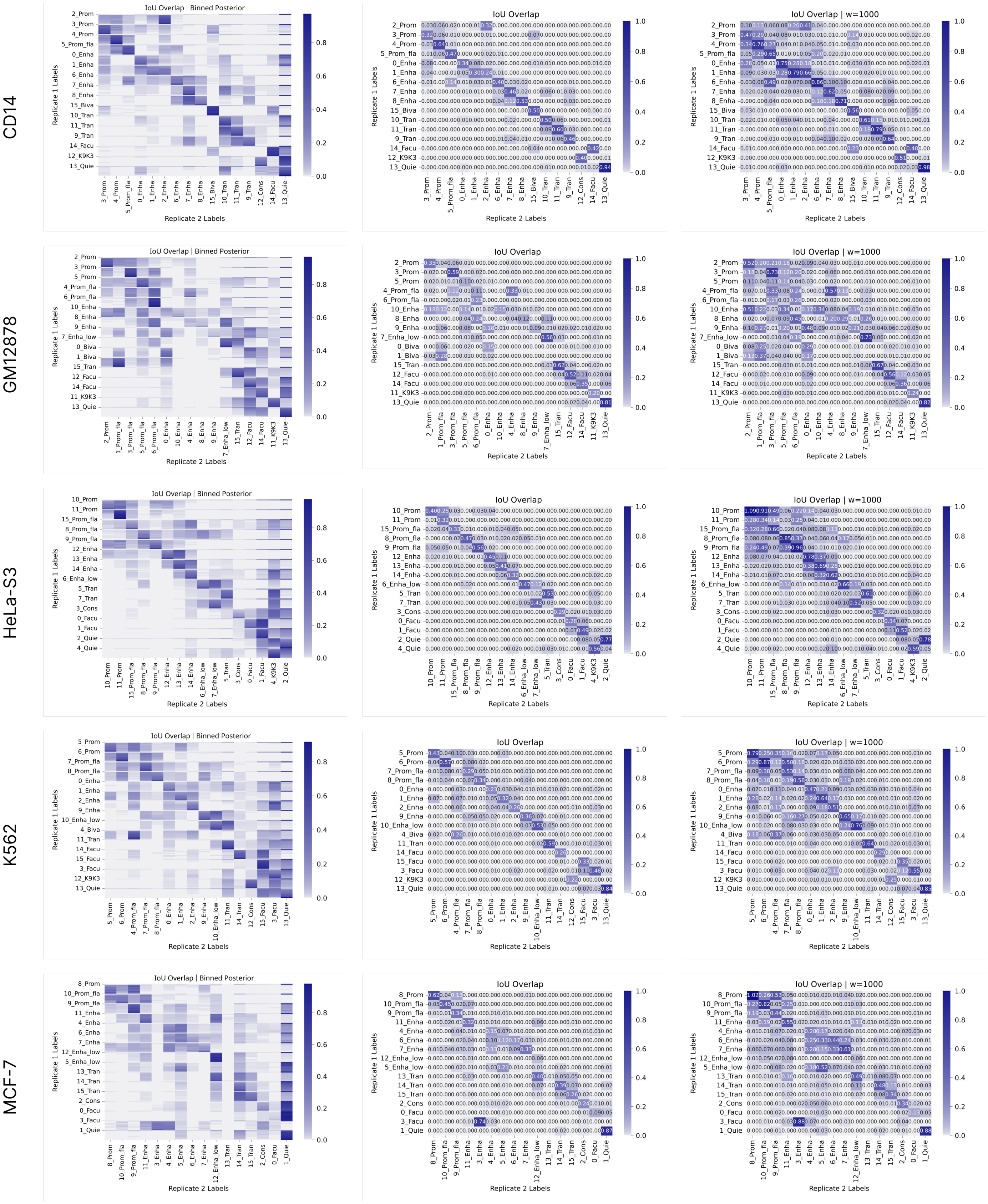

Figure 3: Intersection over union (IoU) overlap of ChromHMM according to (setting 2: different data, same model). Columns from left to right are binned posterior, IoU overlap with maximum-a-posterior state with $w = 0$, IoU overlap with maximum-a-posterior state with $w = 1000$. Color axis is linear and rows correspond to five cell types.

2.2.2 Segway

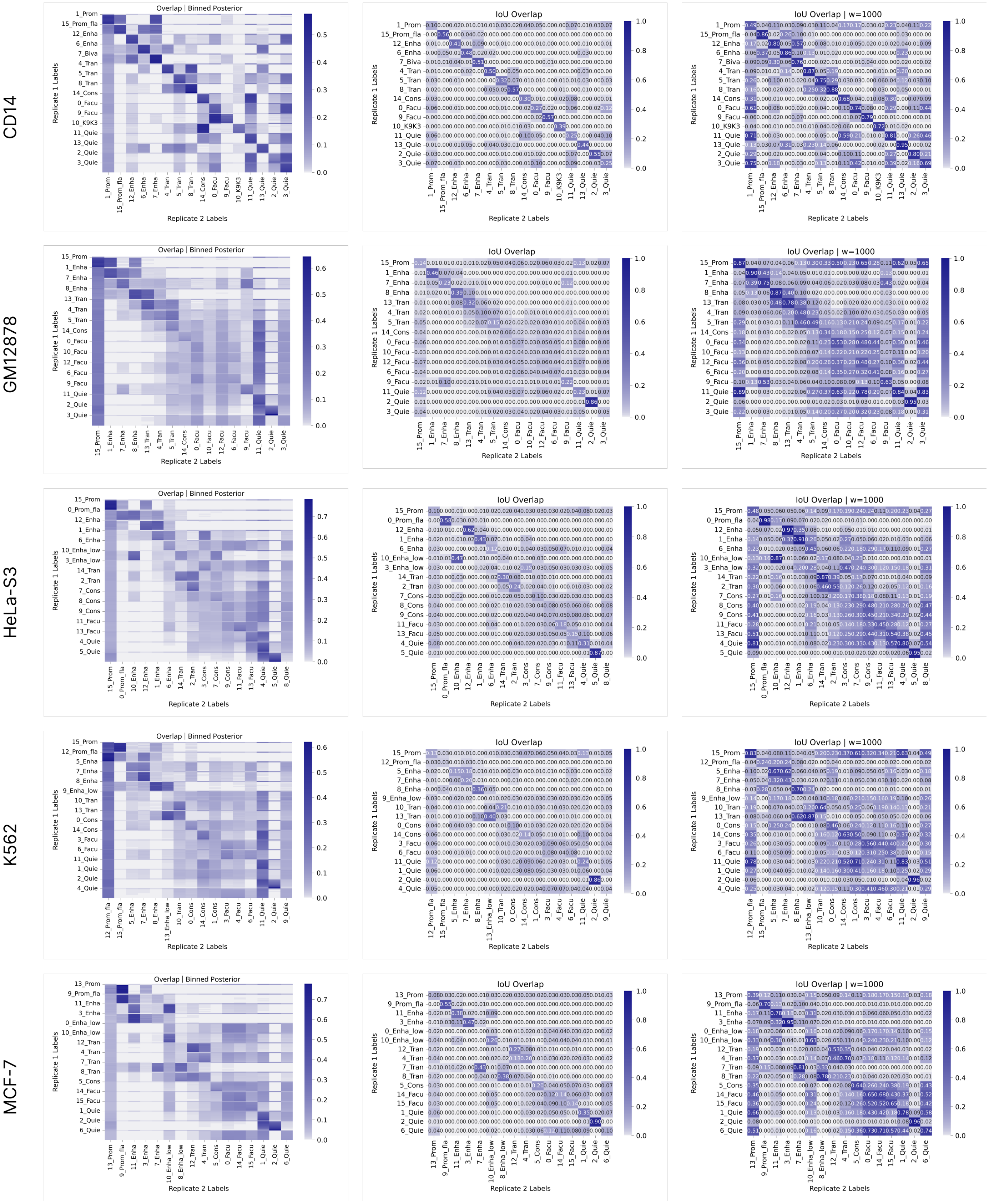

Figure 4: Intersection over union (IoU) overlap of Segway according to (setting 2: different data, same model). Columns from left to right are binned posterior, IoU overlap with maximum-a-posterior state with $w = 0$, IoU overlap with maximum-a-posterior state with $w = 1000$. Color axis is linear and rows correspond to five cell types.

2.3 Setting 3: Same Data, Different Models

2.3.1 ChromHMM

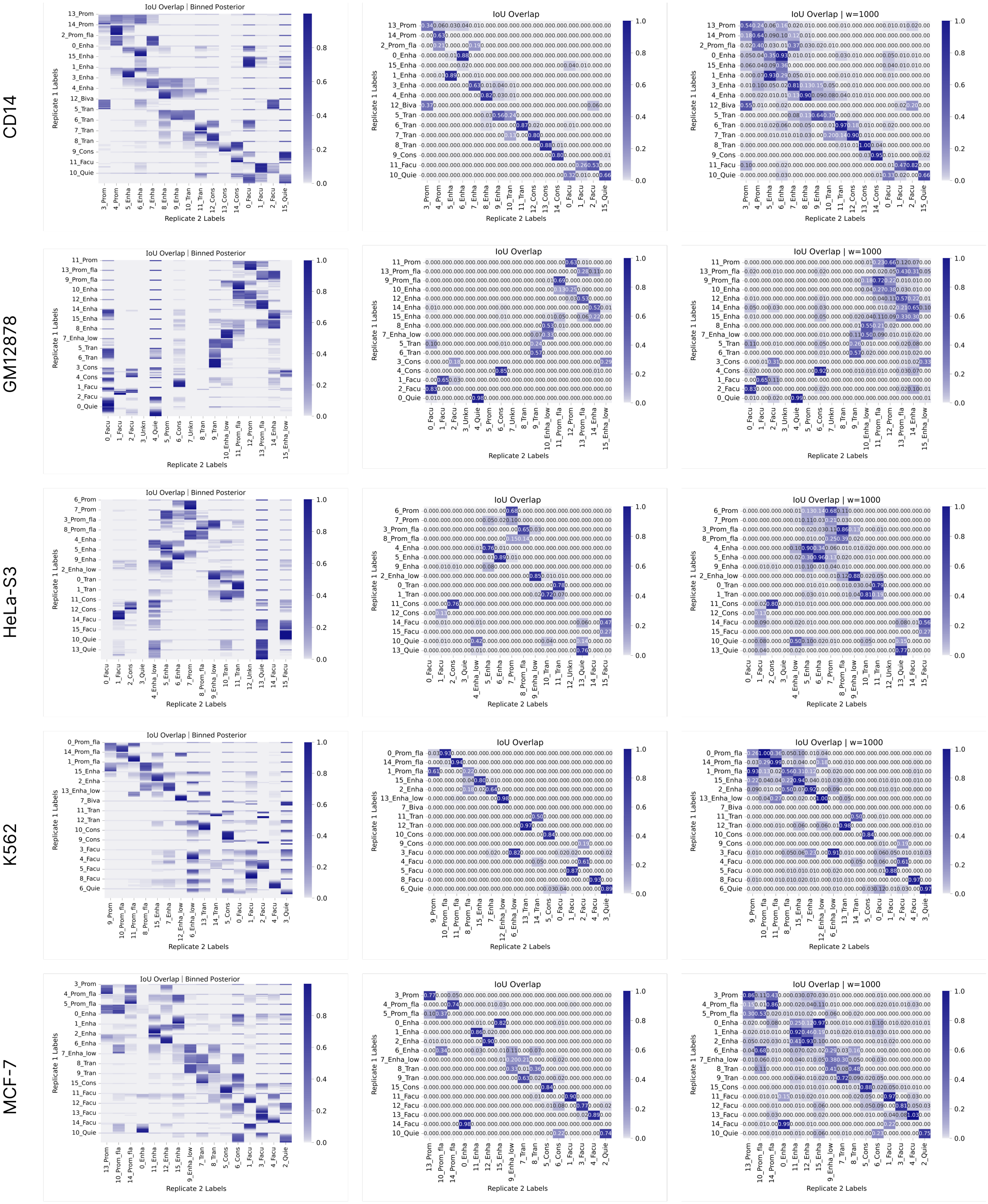

Figure 5: Intersection over union (IoU) overlap of ChromHMM according to (Setting 3: Same Data, Different Models). Columns from left to right are binned posterior, IoU overlap with maximum-a-posterior state with $w = 0$, IoU overlap with maximum-a-posterior state with $w = 1000$. Color axis is linear and rows correspond to five cell types.

2.3.2 Segway

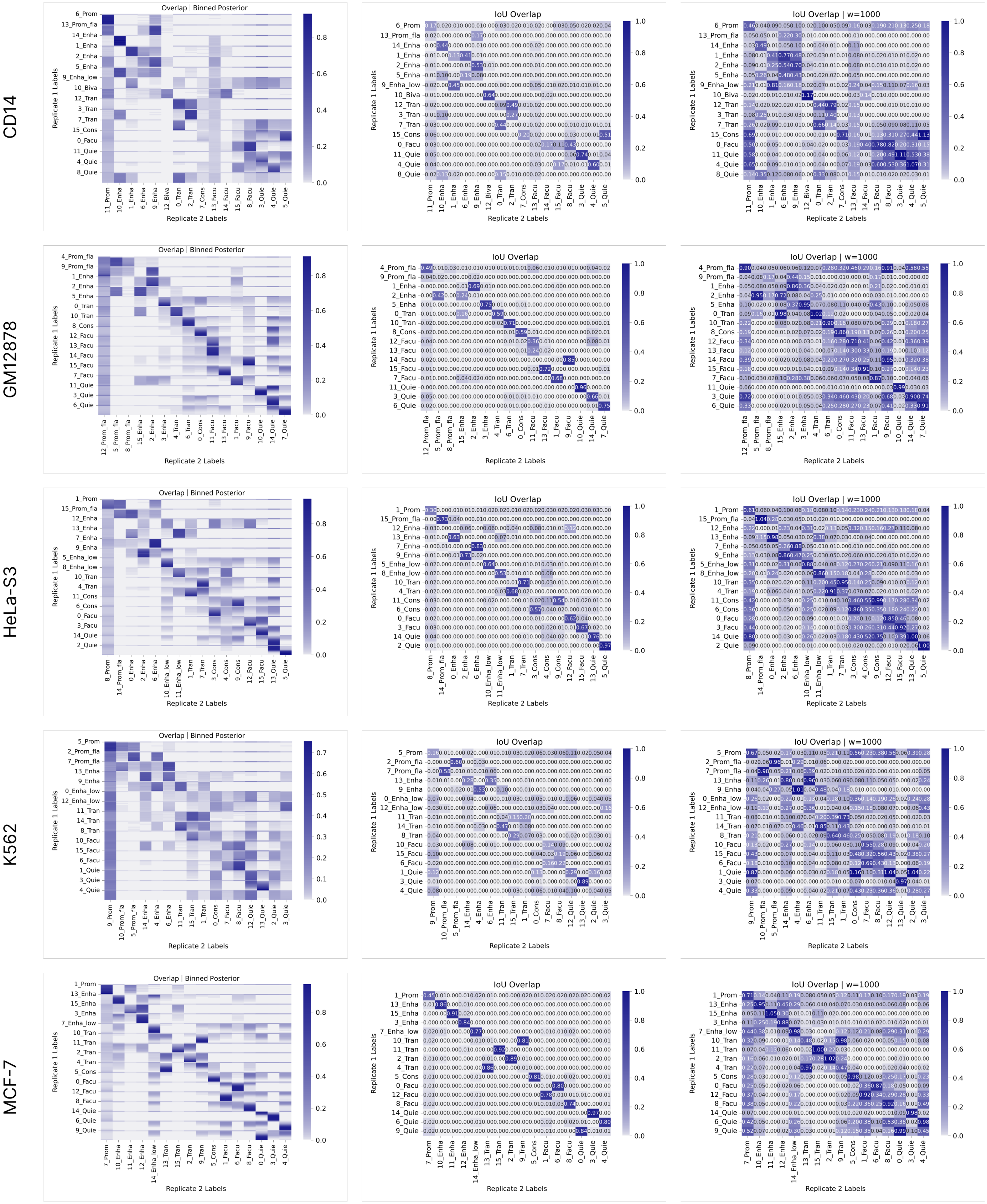

Figure 6: Intersection over union (IoU) overlap of Segway according to (Setting 3: Same Data, Different Models). Columns from left to right are binned posterior, IoU overlap with maximum-a-posterior state with $w = 0$, IoU overlap with maximum-a-posterior state with $w = 1000$. Color axis is linear and rows correspond to five cell types.

2.4 Overlap and emission similarity can identify corresponding states

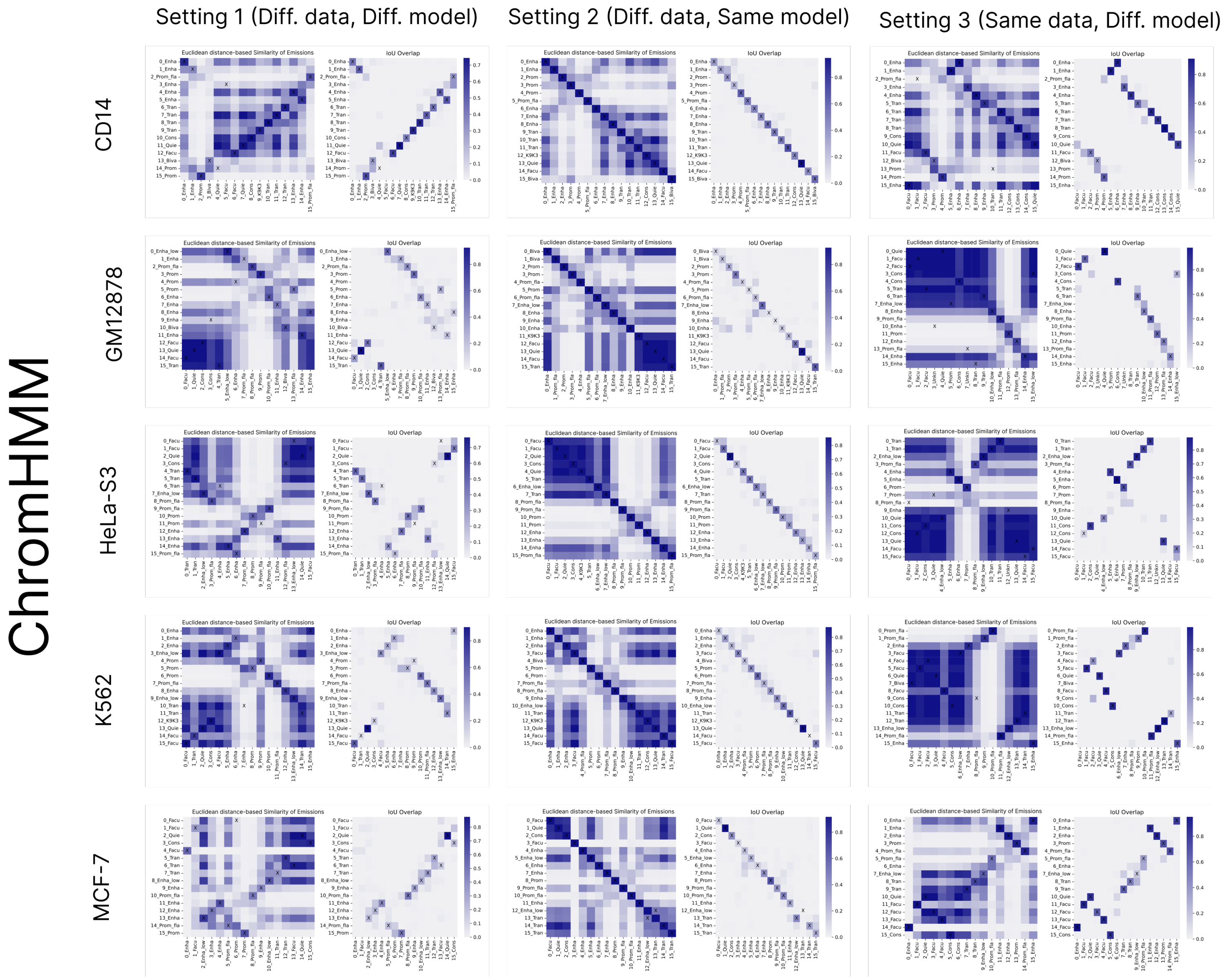

Figure 7: Two heatmaps for chromatin state similarity. The left heatmap illustrates the Euclidean distance-based similarity of emission probabilities of each pair of chromatin states in base and verification annotations. The right heatmap shows the pairwise intersection over union (IoU) of overlap for the same chromatin states. Identified corresponding states are marked with 'X'.

3 Chromatin states overlap differently across replicates

3.1 Naive overlap

3.1.1 ChromHMM

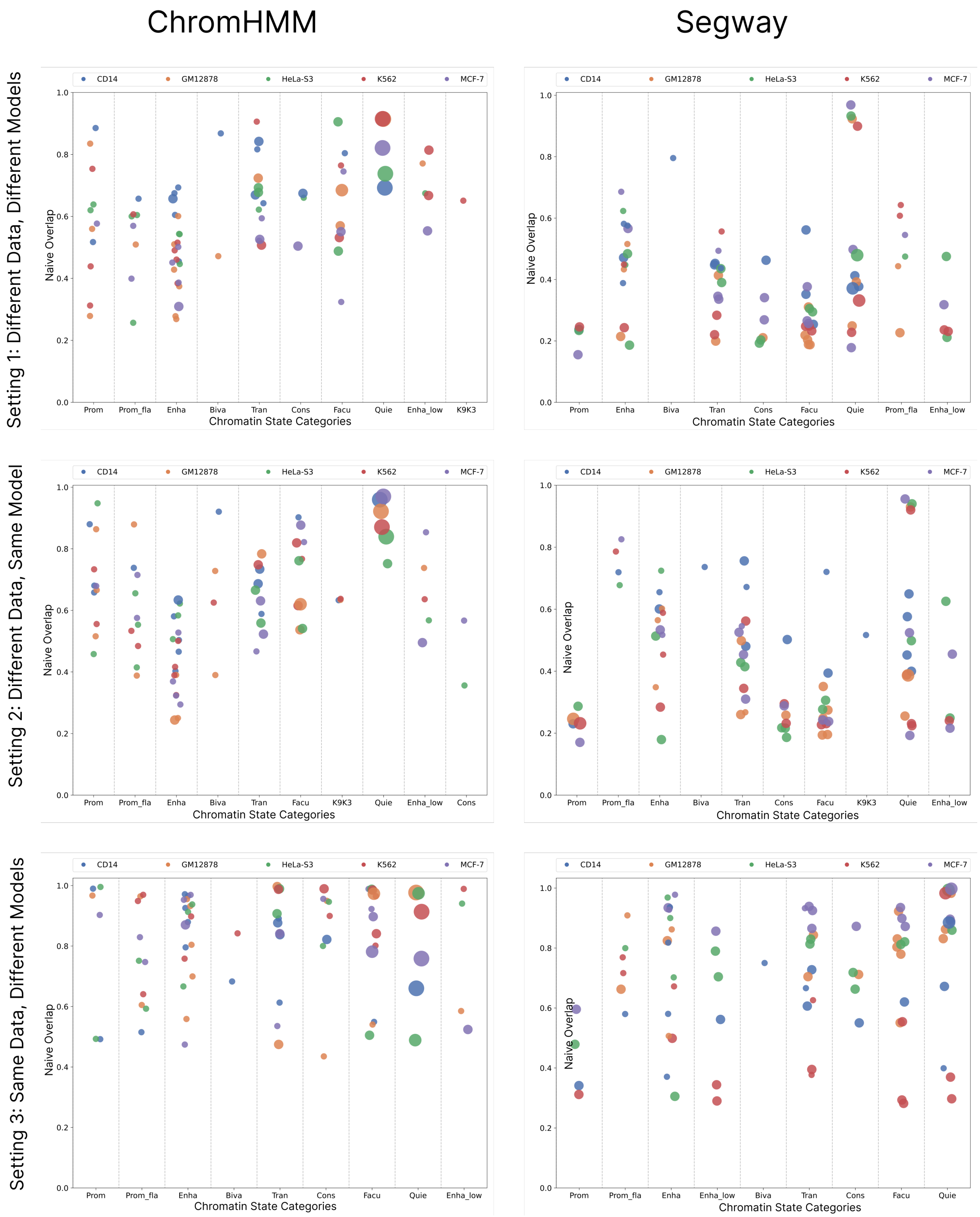

Figure 8: Fraction of overlap (Naive overlap) of various chromatin states categories identified in the ChromHMM and Segway annotation according to three settings for all five cell types. Each dot represents a chromatin state, with color denoting cell type and size proportional to genome coverage

3.2 Granularity of Chromatin States

3.2.1 ChromHMM

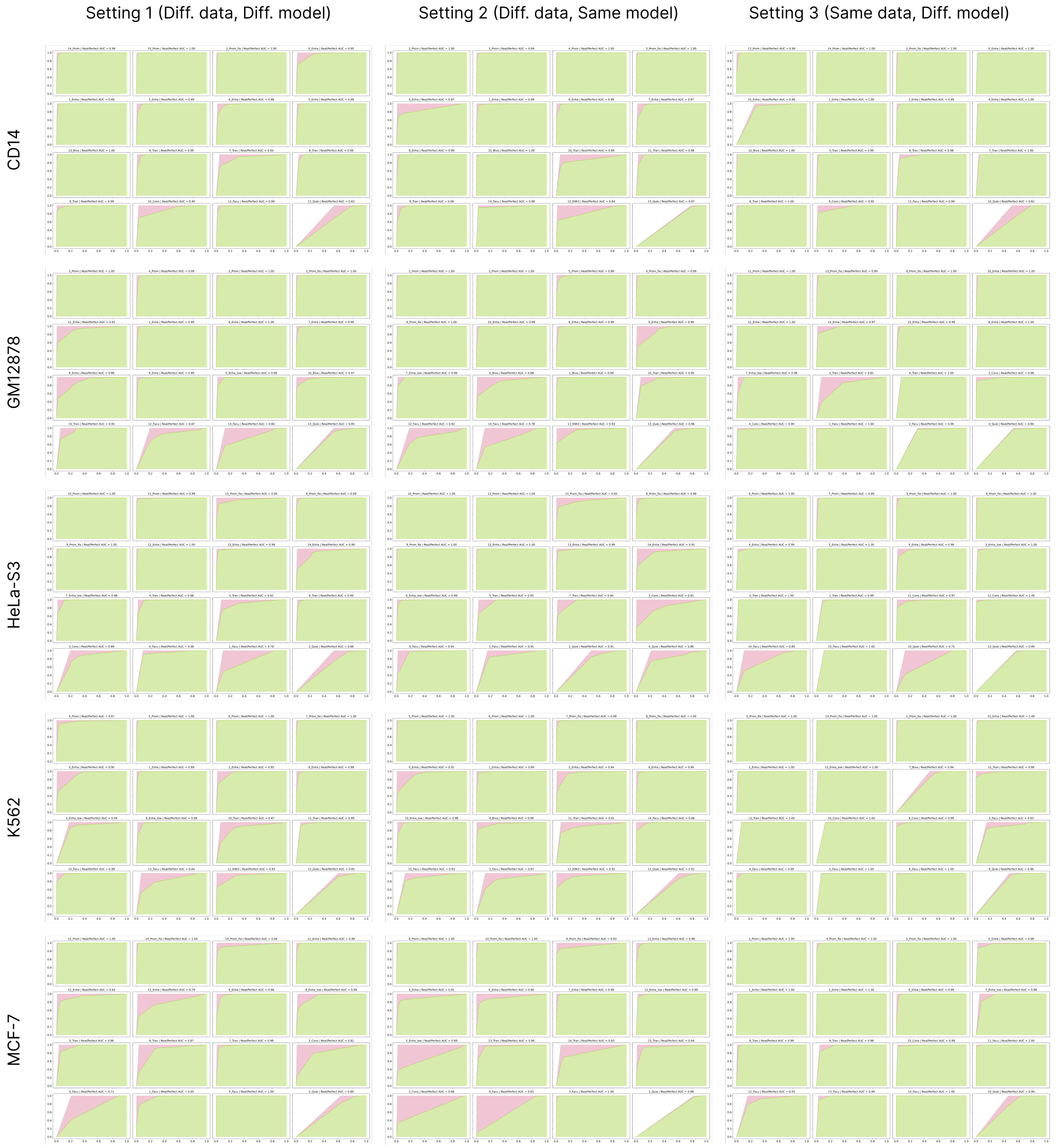

Figure 9: State merging curve for ChromHMM runs. Columns correspond to different settings and rows correspond to five cell types. The area under the state merging curve (auSMC) ratio is a numerical representation of chromatin state reproducibility as a function of chromatin state granularity which is calculated by dividing the observed AUC by the AUC pertaining to the perfect reproducibility case.

3.2.2 Segway

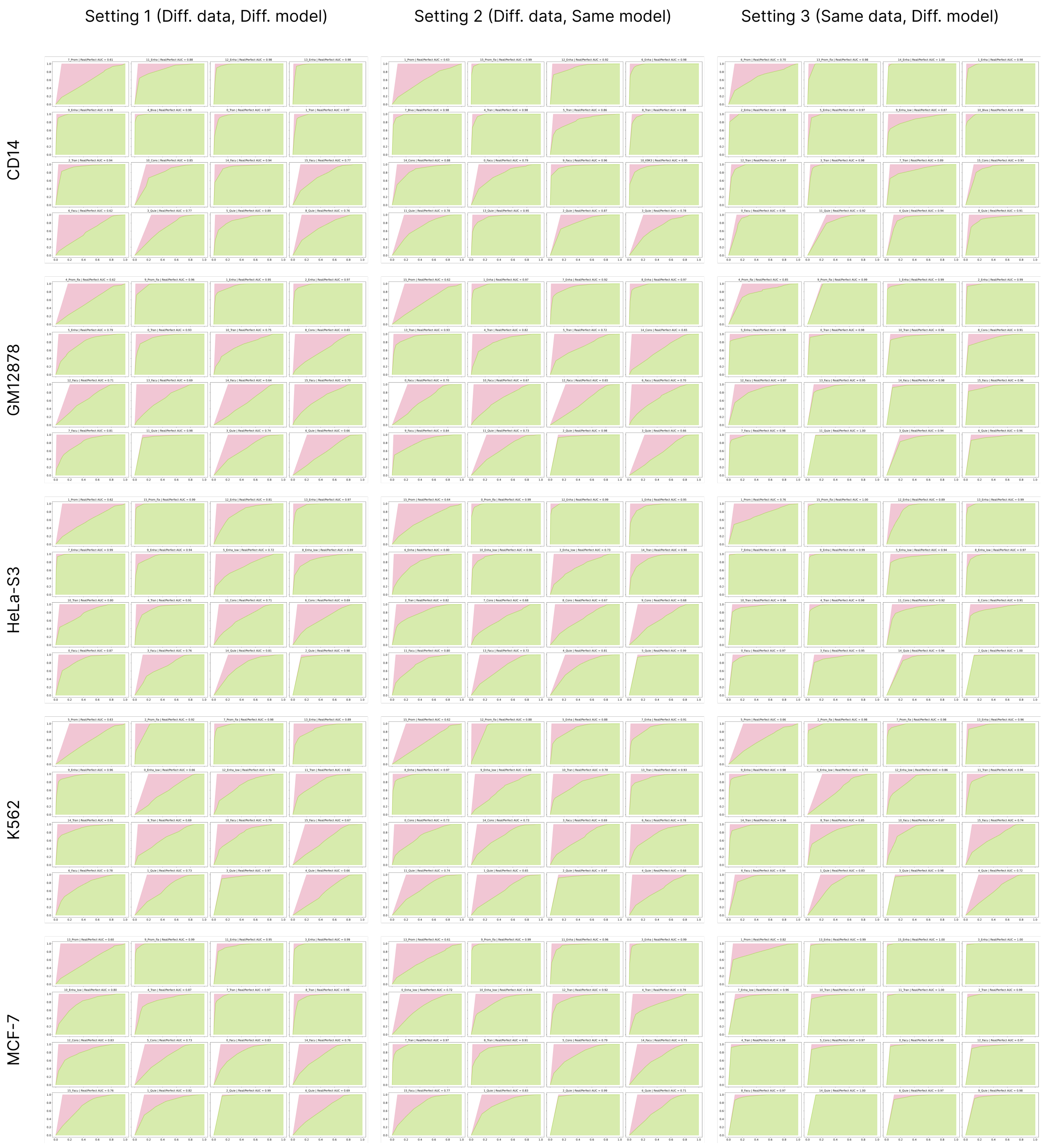

Figure 10: State merging curve for Segway runs. Columns correspond to different settings and rows correspond to five cell types. The area under the state merging curve (auSMC) ratio is a numerical representation of chromatin state reproducibility as a function of chromatin state granularity which is calculated by dividing the observed AUC by the AUC pertaining to the perfect reproducibility case.

3.3 Area under state merging curve (auSMC) for various genomic functions

3.3.1 ChromHMM

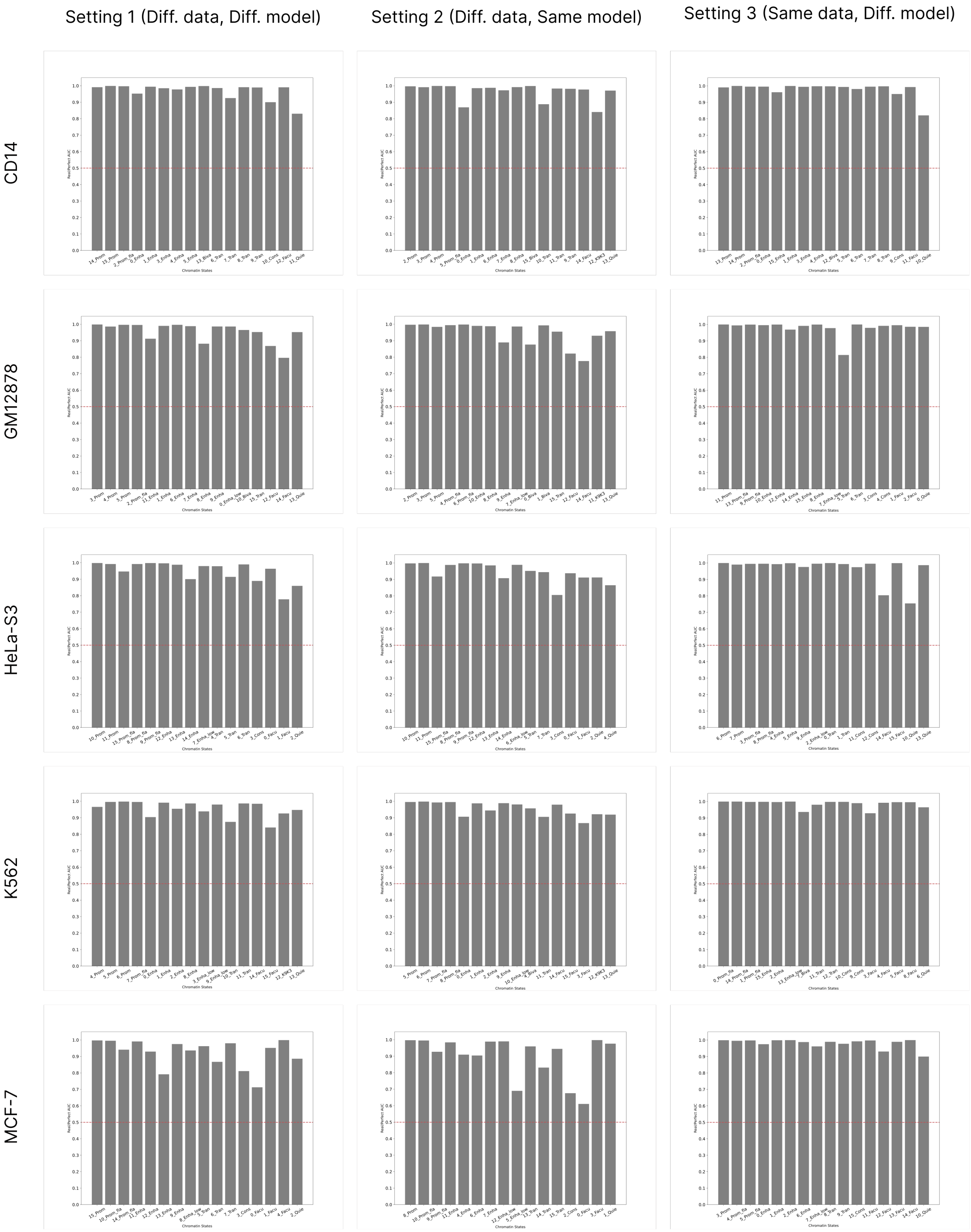

Figure 11: The auSMC ratio of chromatin states in the base ChromHMM annotations. Rows correspond to different cell types and columns correspond to different settings of variability.

3.3.2 Segway

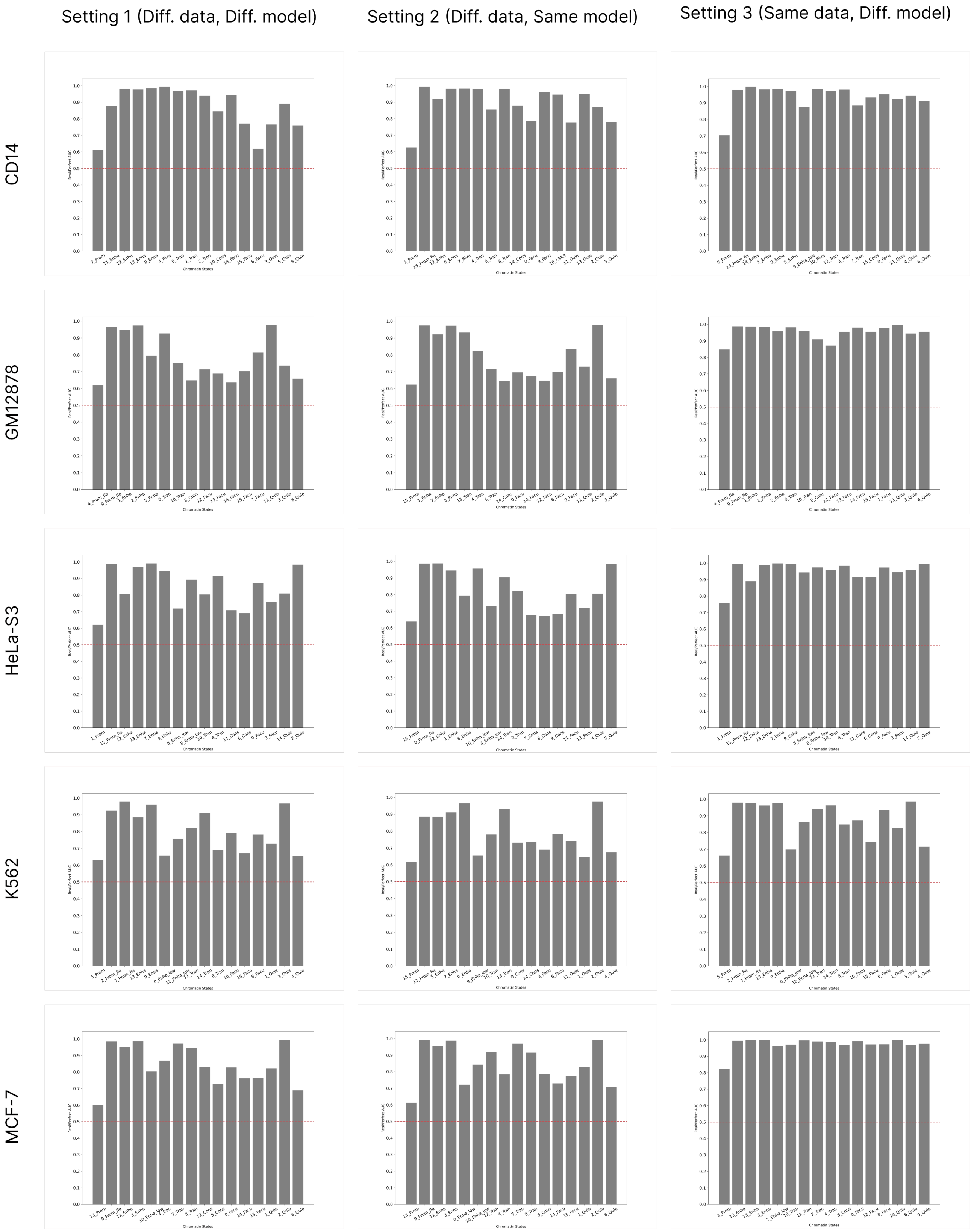

Figure 12: The auSMC ratio of chromatin states in the base Segway annotations. Rows correspond to different cell types and columns correspond to different settings of variability.

3.3.3 Area under state merging curve (auSMC) chromatin state categories

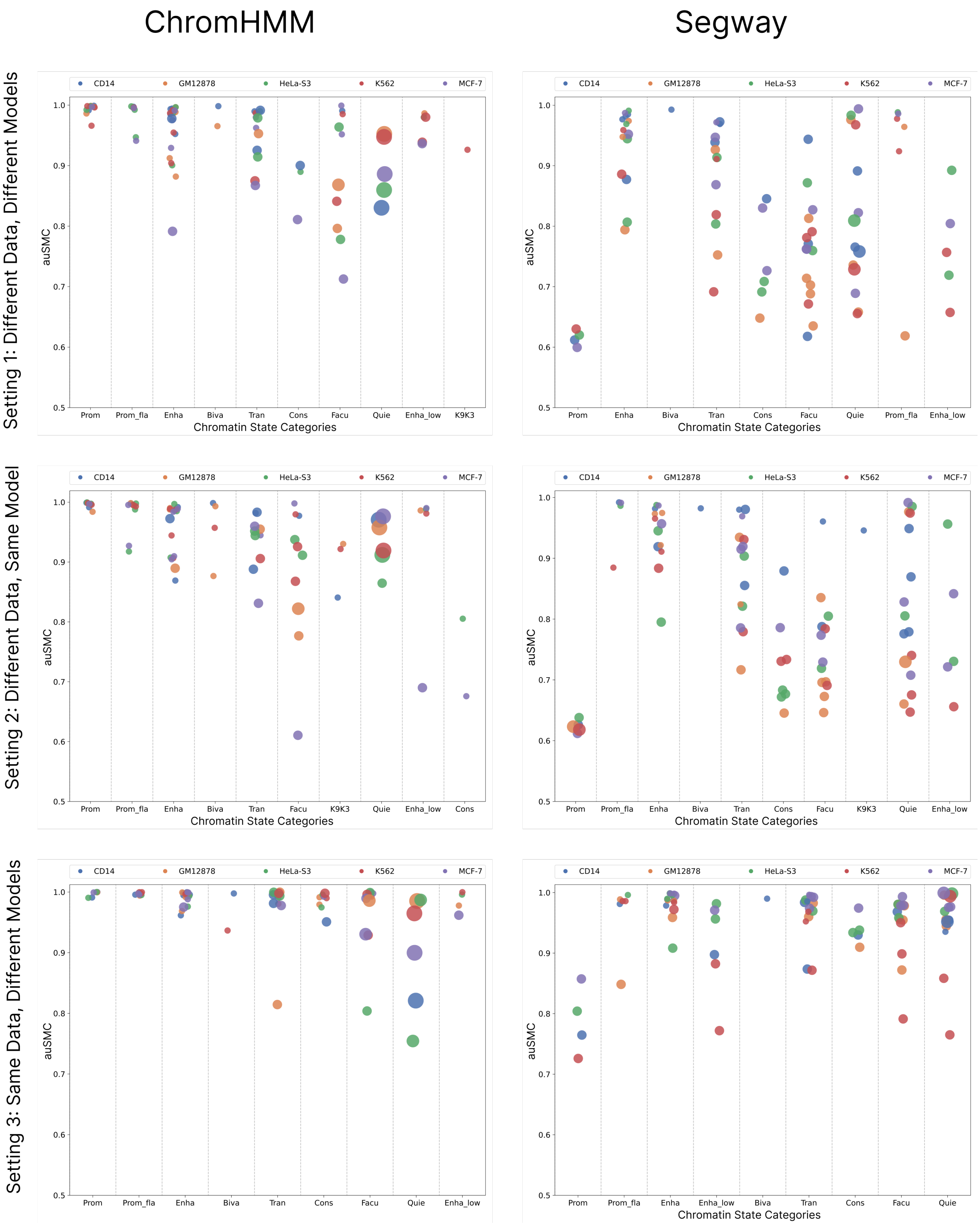

Figure 13: auSMC ratio of various chromatin states categories identified in the ChromHMM (left column) and Segway (right column) annotation according to three settings of variability (rows) for five cell types. Each dot represents a chromatin state, with color denoting cell type and size proportional to genome coverage.

3.4 Post-Clustering

3.4.1 Mutual Information

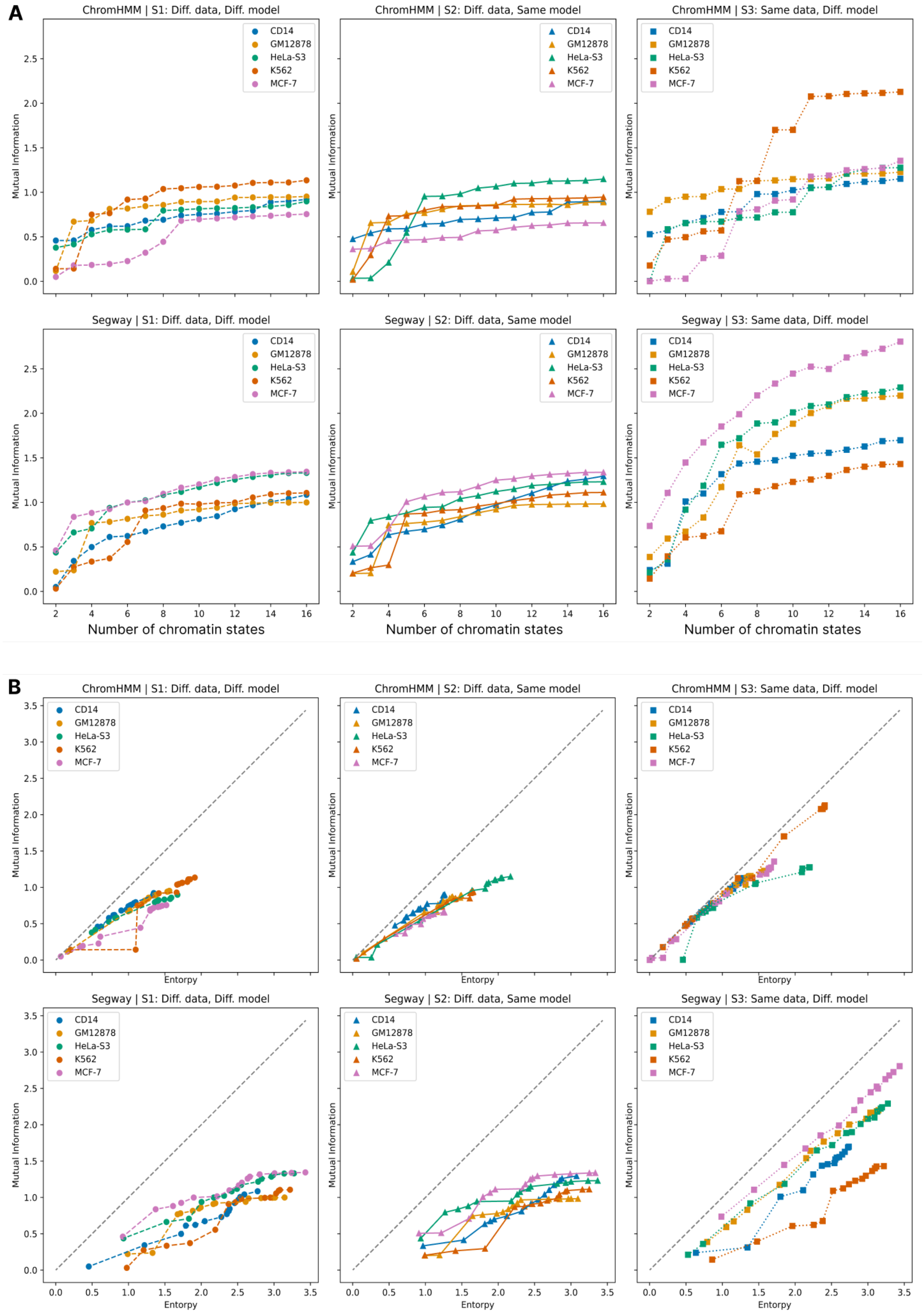

Figure 14: **(A)** Mutual information as a function of number of states of annotations measured during the post-clustering process. **(B)** Mutual information as a function of entropy of annotations measured during the post-clustering process.

3.4.2 Average r-value

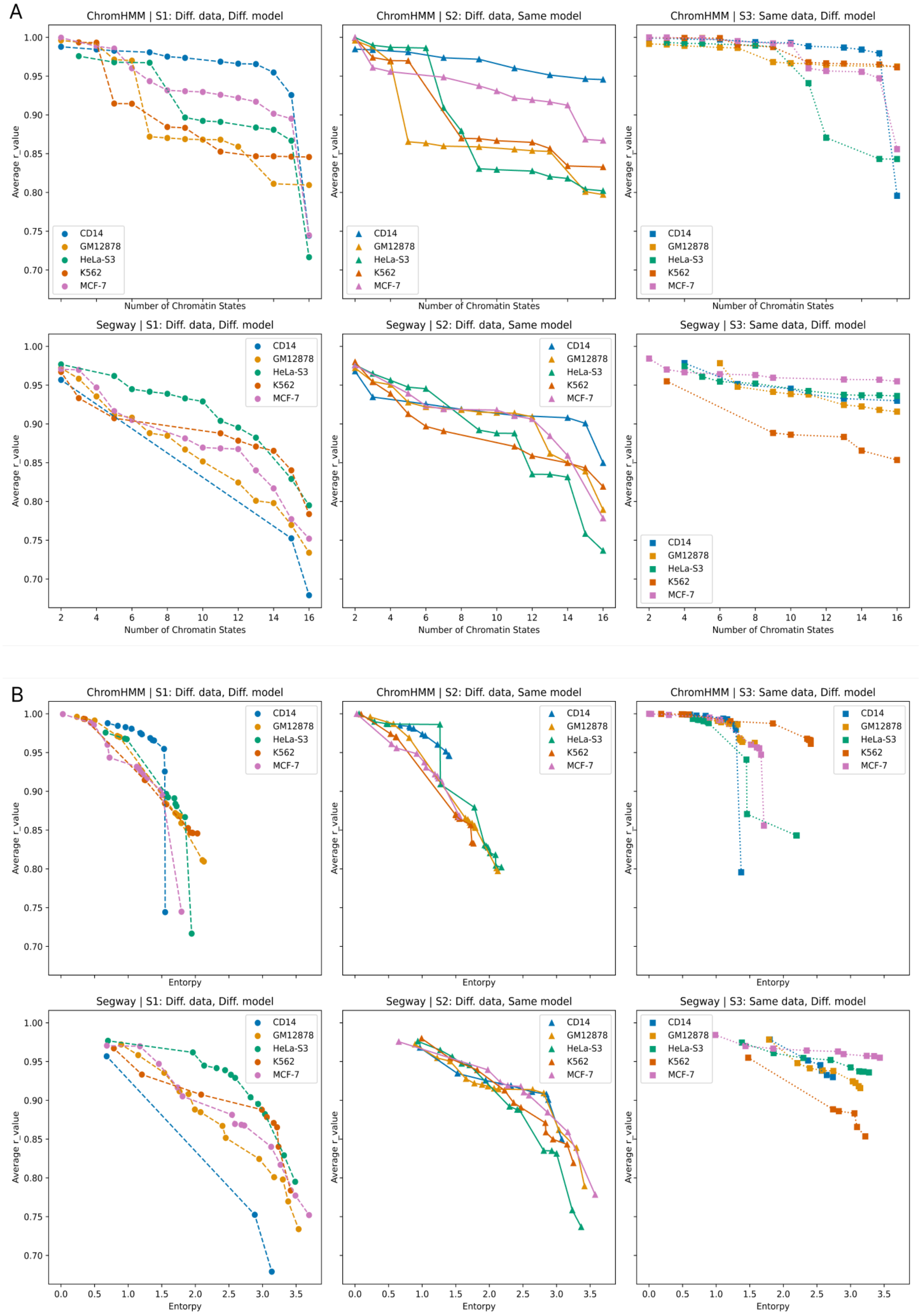

Figure 15: **(A)** Average r-value as a function of number of states of annotations measured during the post-clustering process. **(B)** Average r-value as a function of entropy of annotations measured during the post-clustering process.

3.4.3 Fraction of confident genomic regions

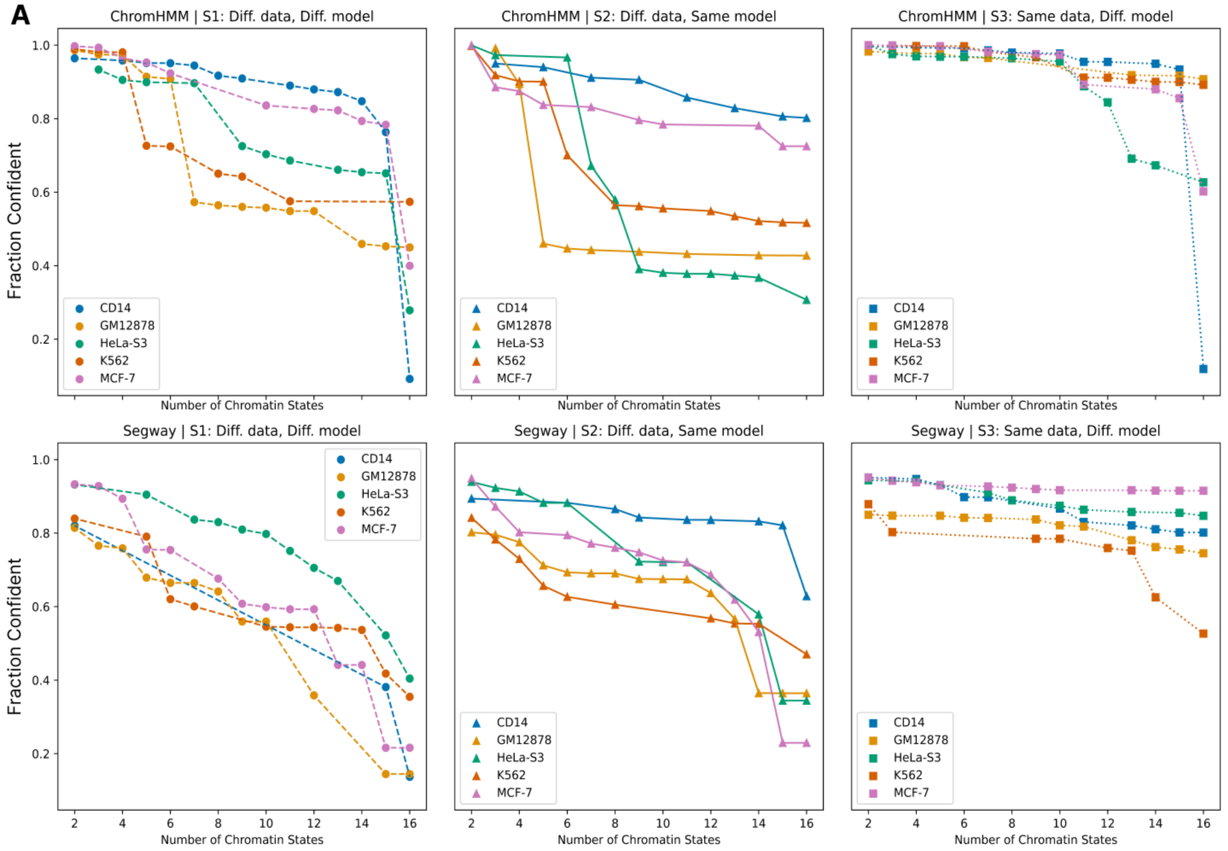

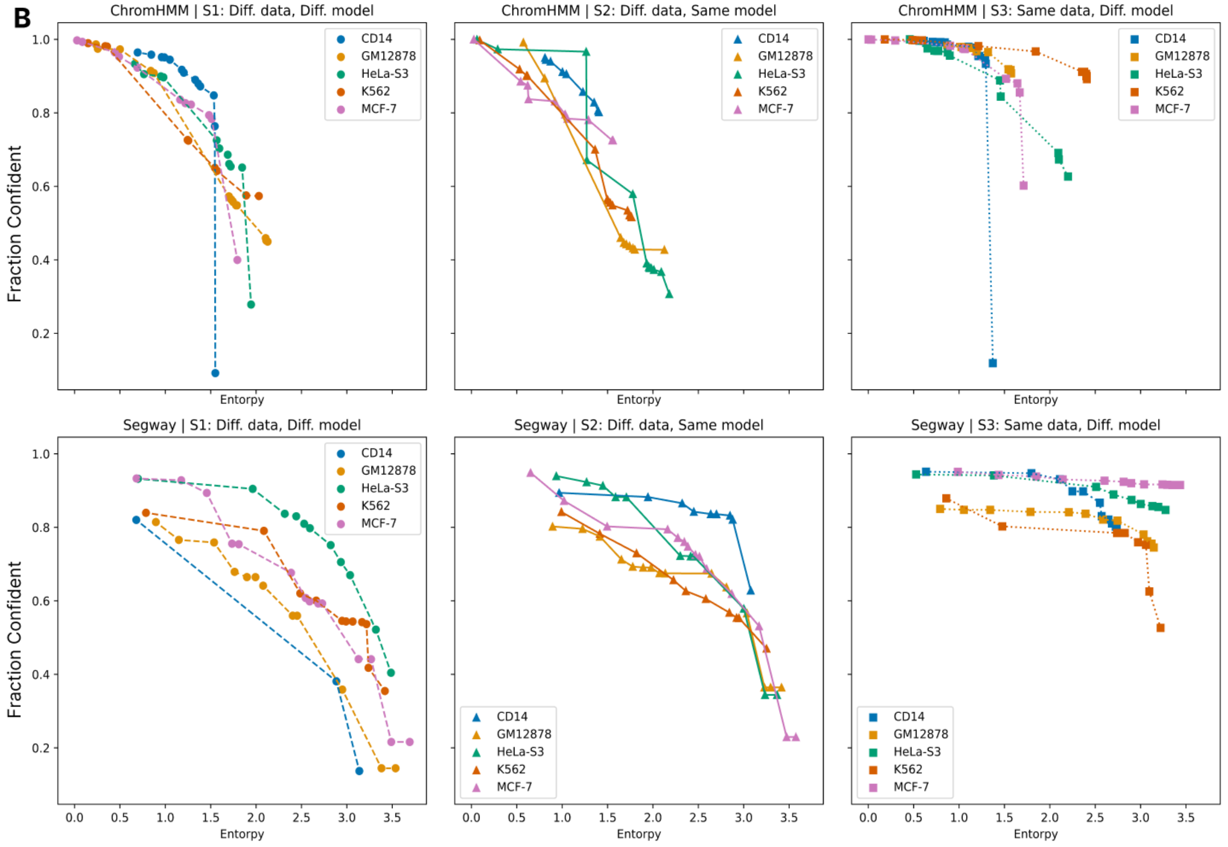

Figure 16: **(A)** Fraction of confident genomic regions as a function of number of states of annotations measured during the post-clustering process. **(B)**Fraction of confident genomic regions as a function of entropy of annotations measured during the post-clustering process.

4 Corresponding chromatin states occur at proximity of each other across replicates

4.1 Overlap as a function of w

4.1.1 ChromHMM

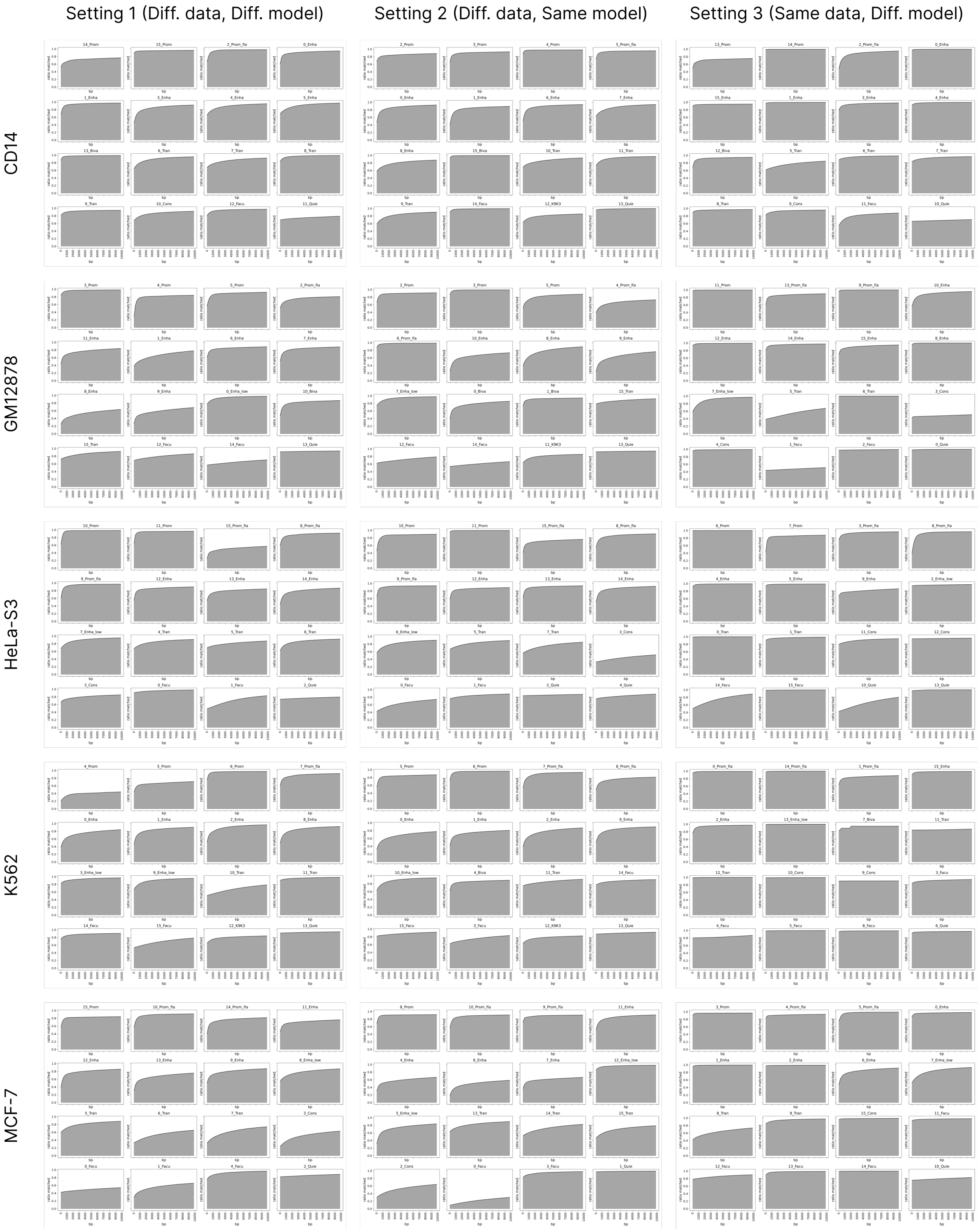

Figure 17: The fraction of each state in base annotation that is overlapped with a corresponding state within a w-sized distance upstream or downstream in ChromHMM. Rows correspond to different cell types and columns correspond to settings of variability.

4.1.2 Segway

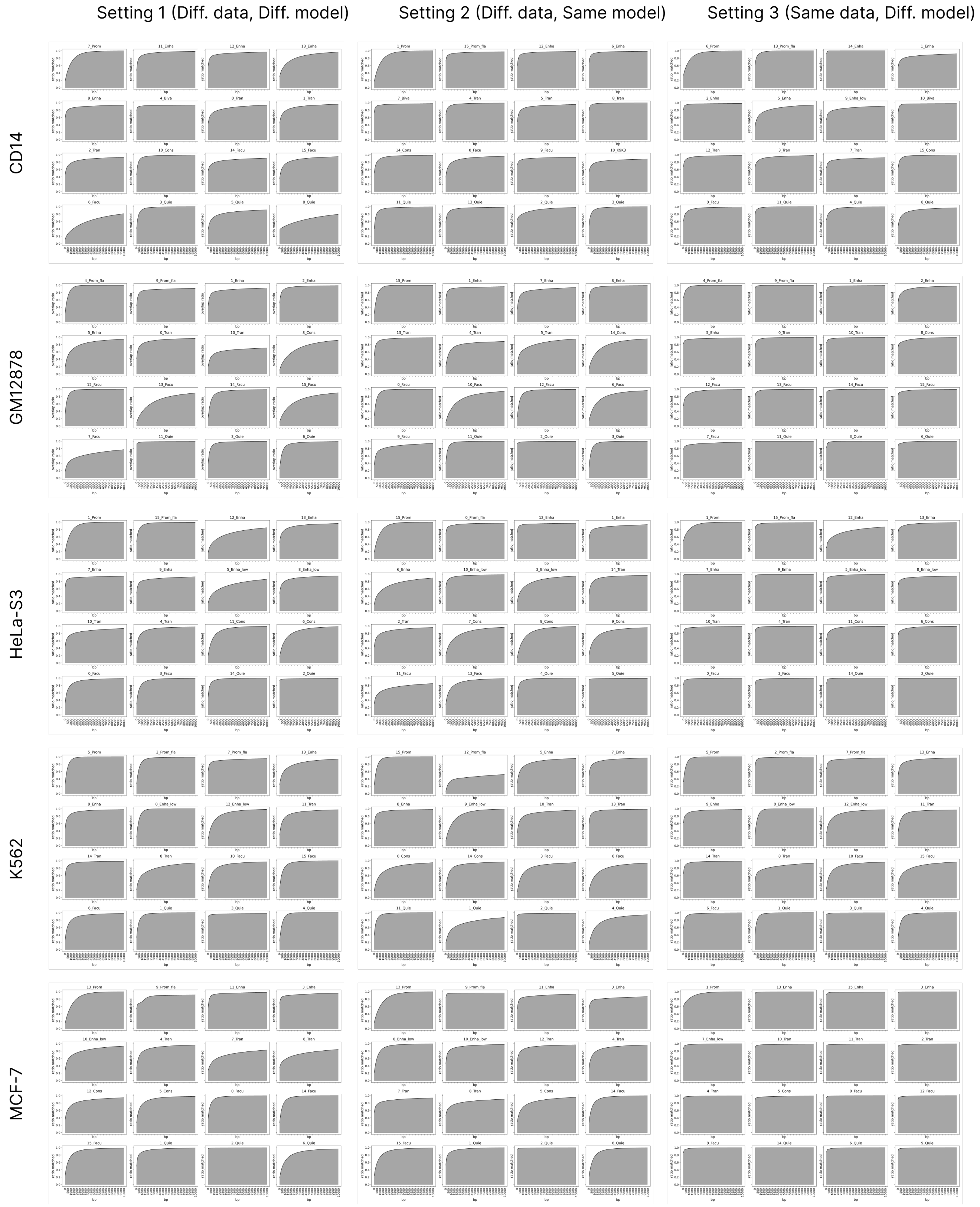

Figure 18: The fraction of each state in base annotation that is overlapped with a corresponding state within a w-sized distance upstream or downstream in Segway. Rows correspond to different cell types and columns correspond to settings of variability.

4.2 Correspondence as a function of w

4.2.1 ChromHMM

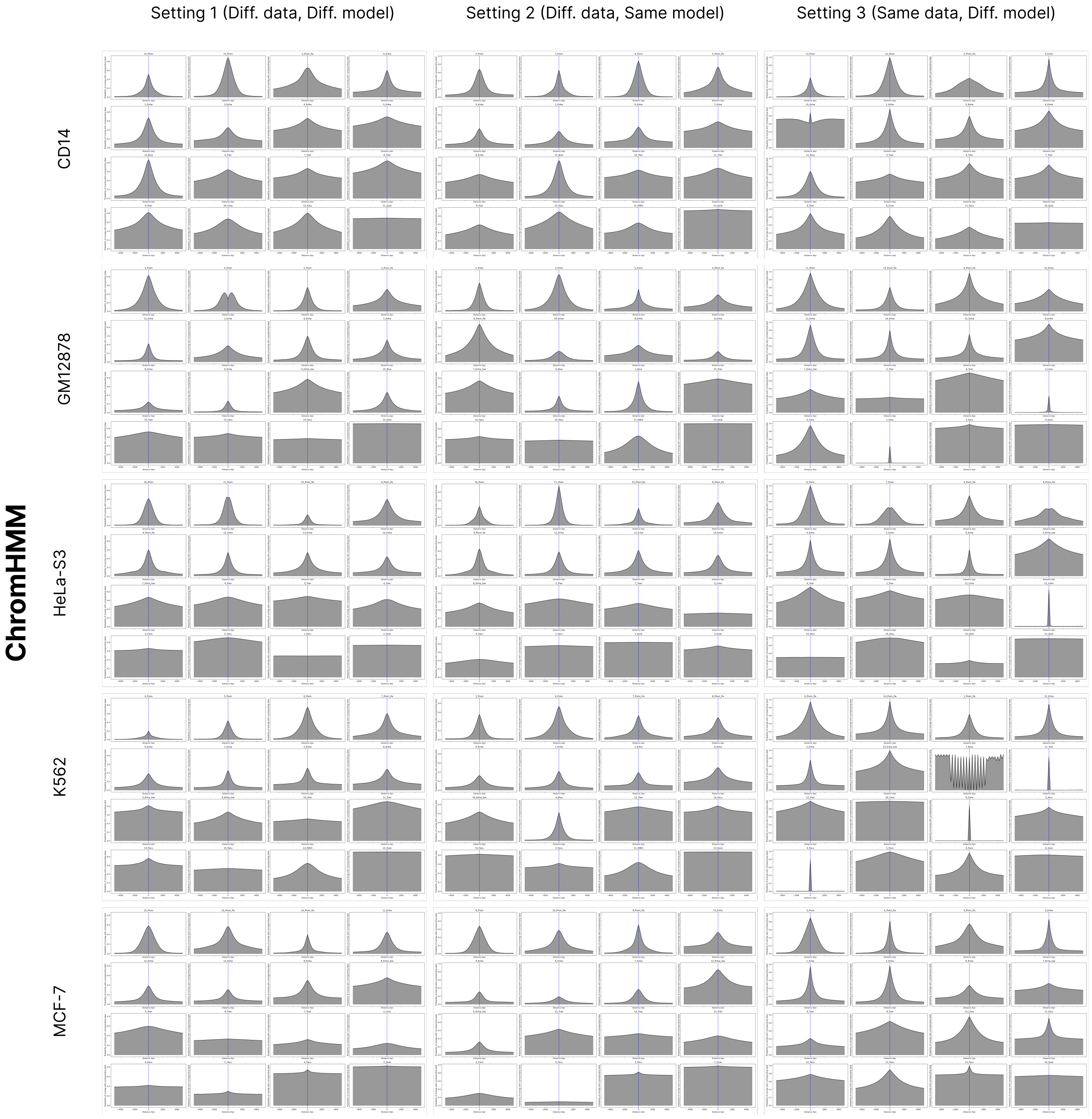

Figure 19: At each position in the genome, the probability of overlap with the corresponding state decreases as a function of distance from the position. Rows correspond to different cell types and columns correspond to settings of variability.

4.2.2 Segway

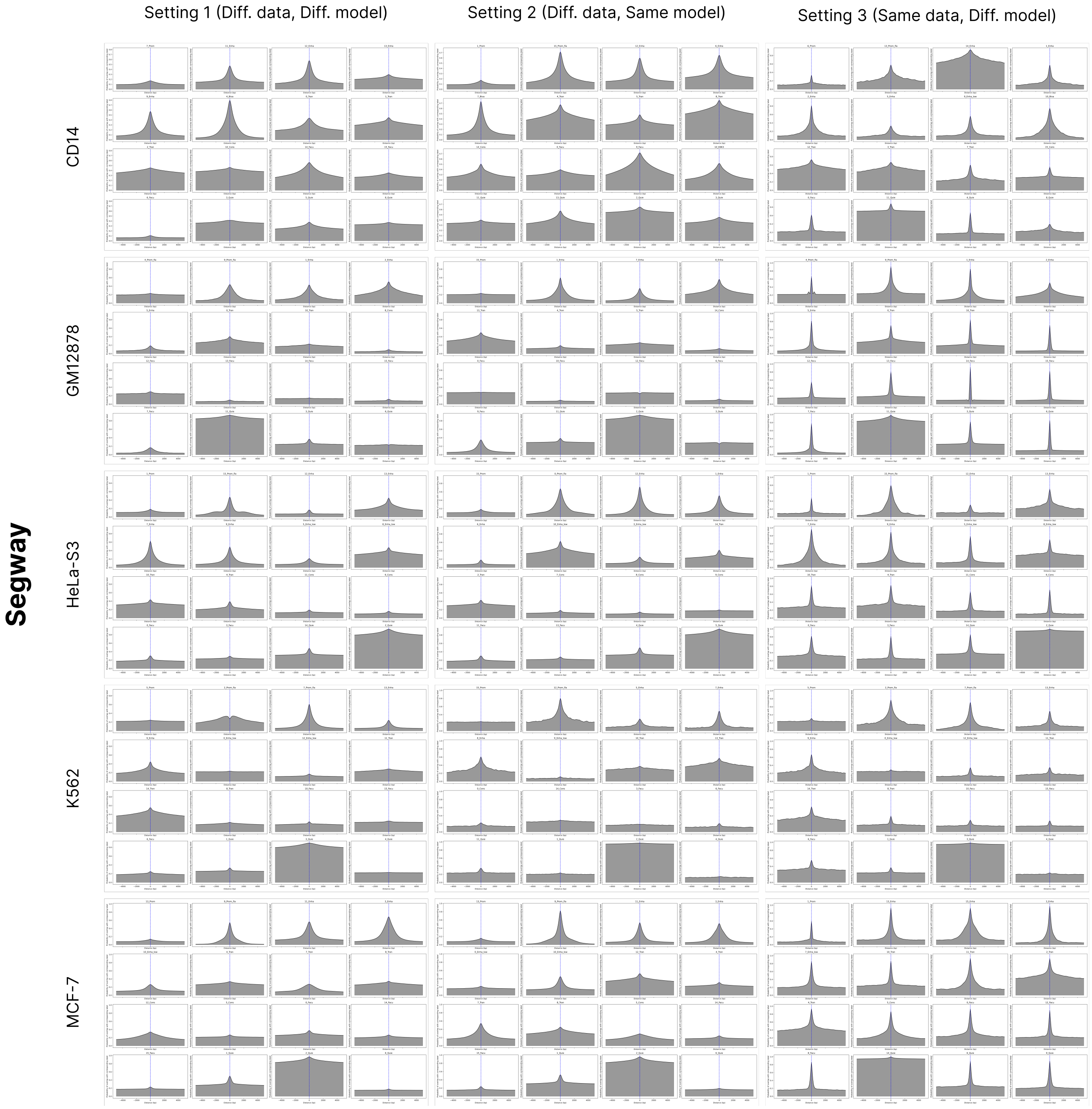

Figure 20: At each position in the genome, the probability of overlap with the corresponding state decreases as a function of distance from the position. Rows correspond to different cell types and columns correspond to settings of variability.

5 Posterior probability is associated with overlap

5.1 Calibration of posterior probability

5.1.1 ChromHMM

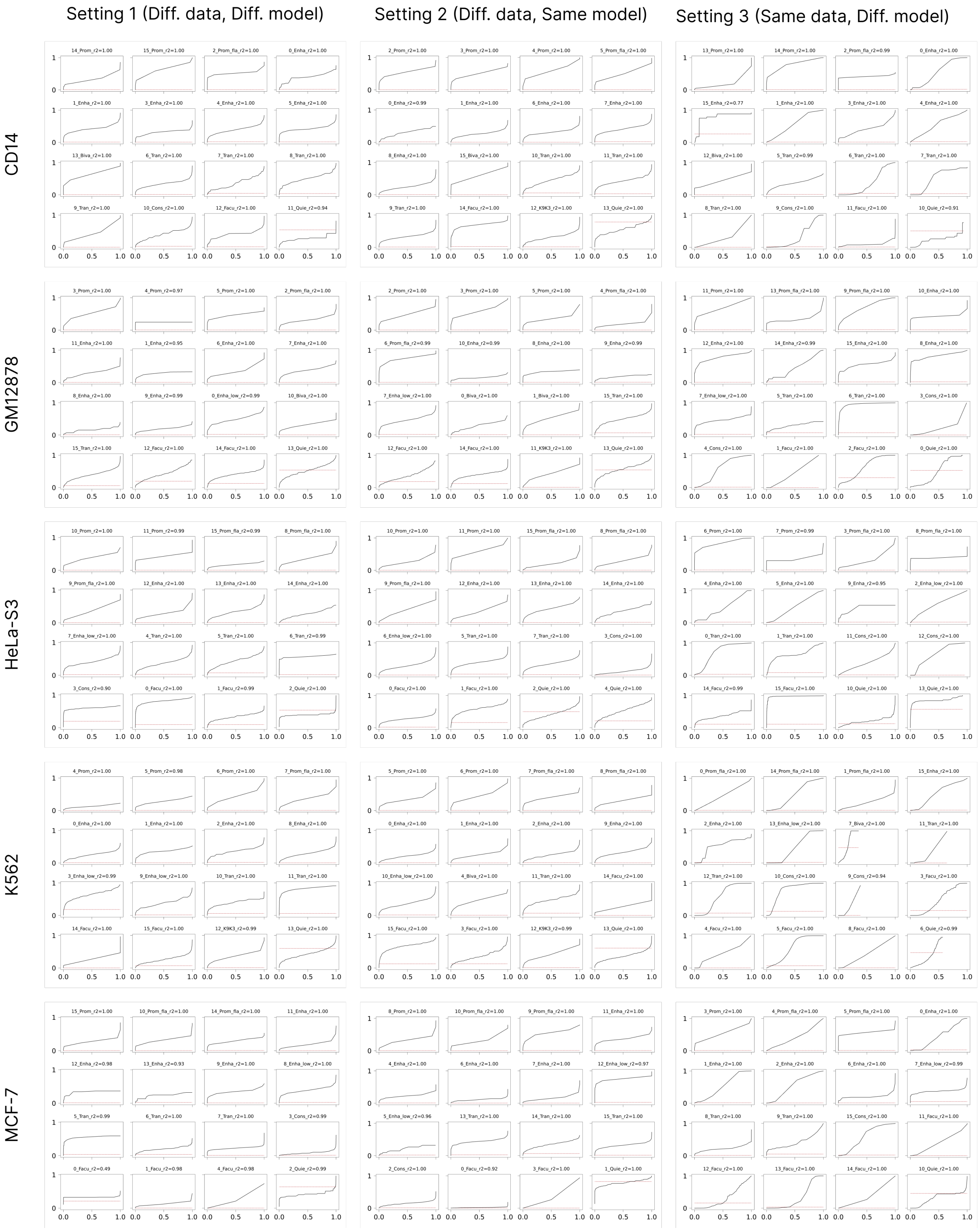

Figure 21: For each chromatin state in the base annotation, we measure the frequency of overlap with a corresponding state as a function of their posterior probability. We observe a clear association between the overlap and the posterior probability. Rows correspond to different cell types and columns correspond to settings of variability.

5.1.2 Segway

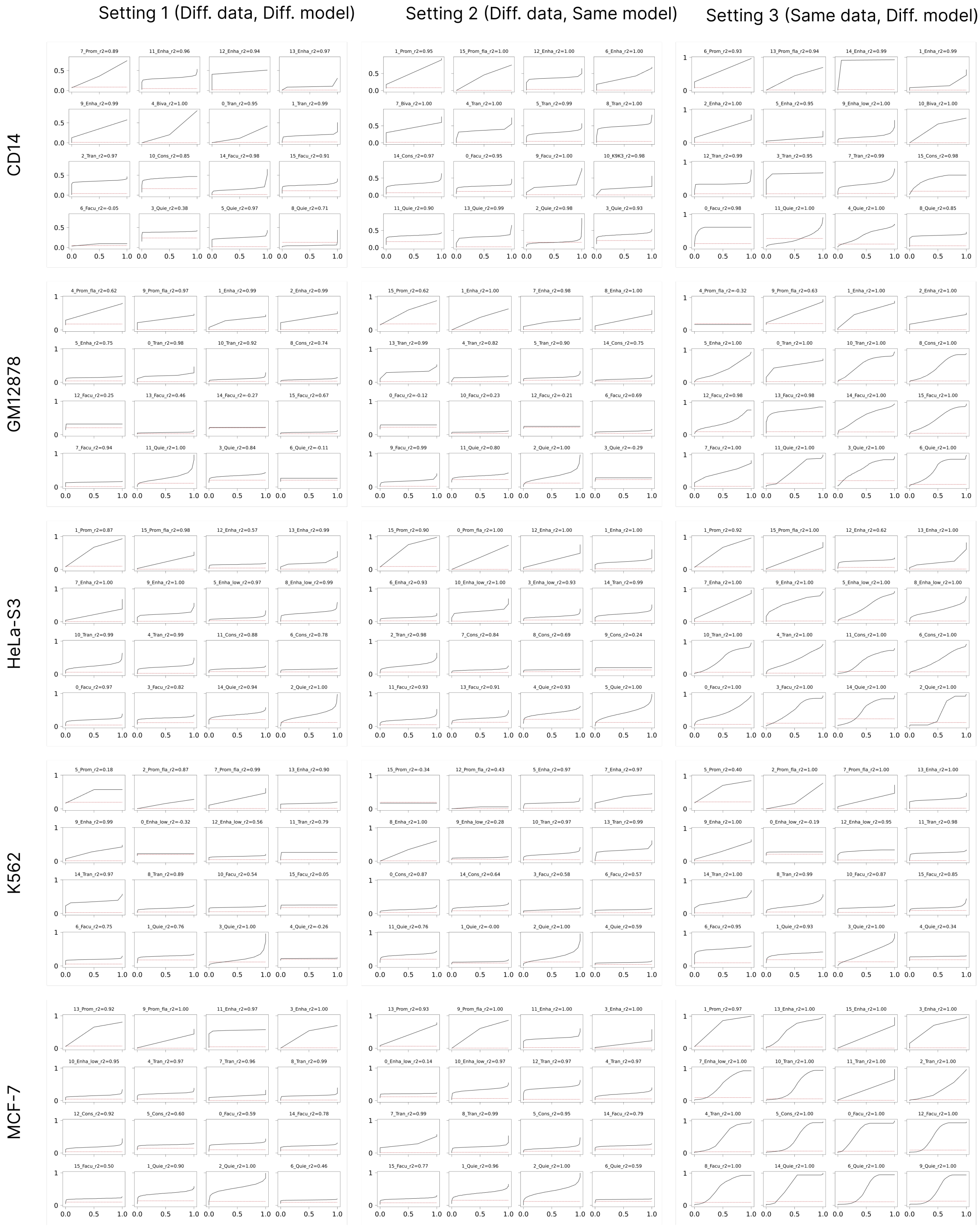

Figure 22: For each chromatin state in the base annotation, we measure the frequency of overlap with a corresponding state as a function of their posterior probability. We observe a clear association between the overlap and the posterior probability. Rows correspond to different cell types and columns correspond to settings of variability.

5.2 Transcription (gene body) as a function of posterior probability

5.2.1 ChromHMM

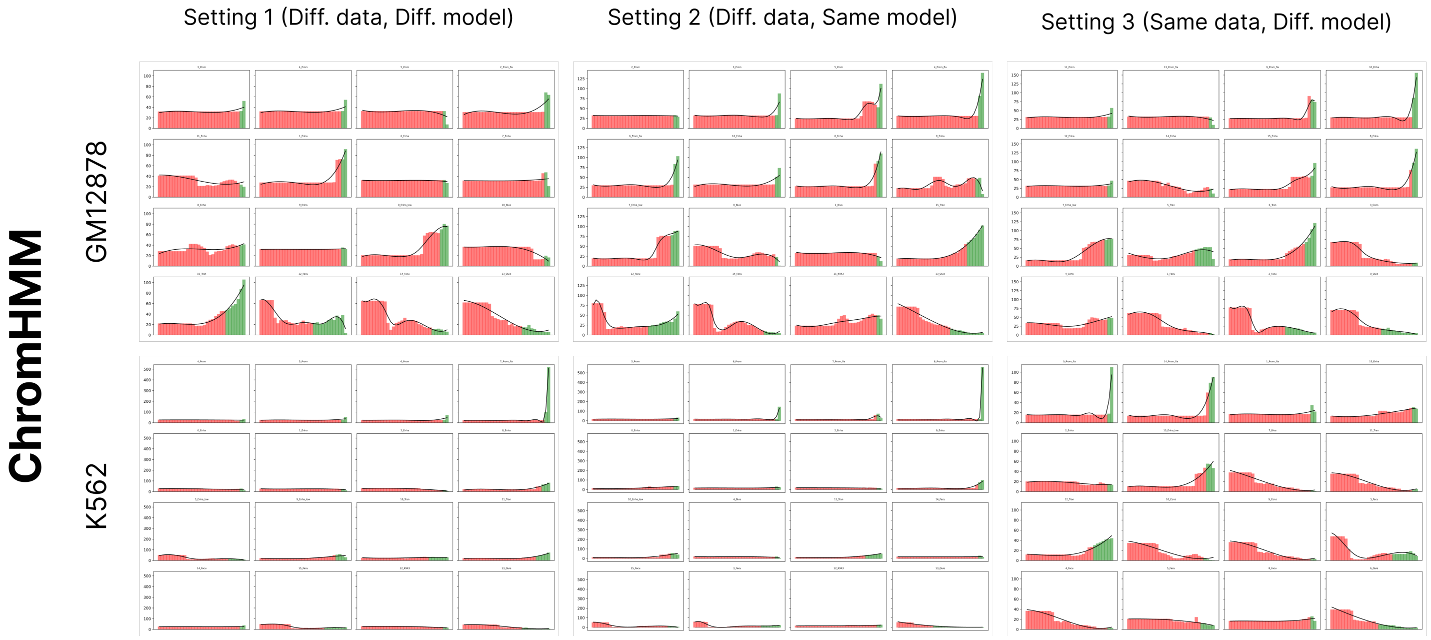

Figure 23: Mean expression level (TPM) in the gene-body as a function of posterior probability for ChromHMM runs in cell types GM12878 and K562. The horizontal axis represents rank of posterior probability, while the vertical axis shows TPM.

5.2.2 Segway

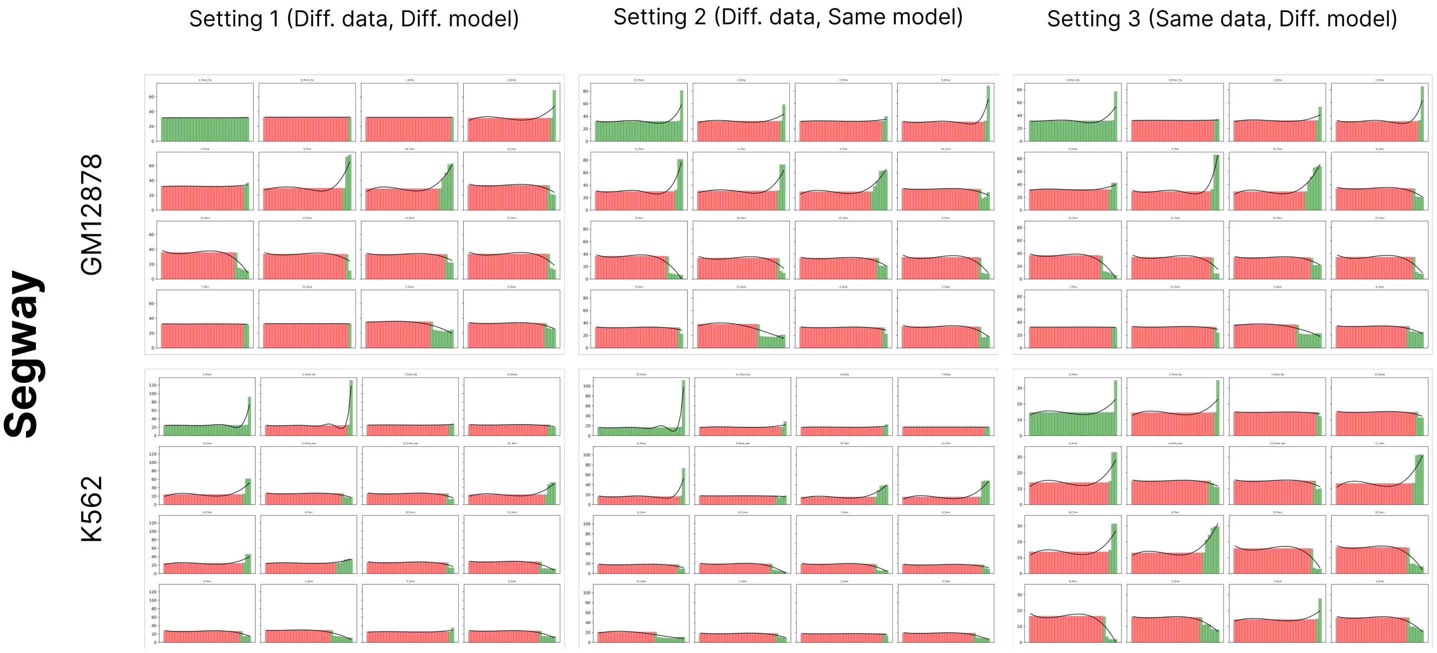

Figure 24: Mean expression level (TPM) in the gene-body as a function of posterior probability for Segway runs in cell types GM12878 and K562. The horizontal axis represents rank of posterior probability, while the vertical axis shows TPM.

5.3 Transcription (around TSS) as a function of posterior probability

5.3.1 ChromHMM

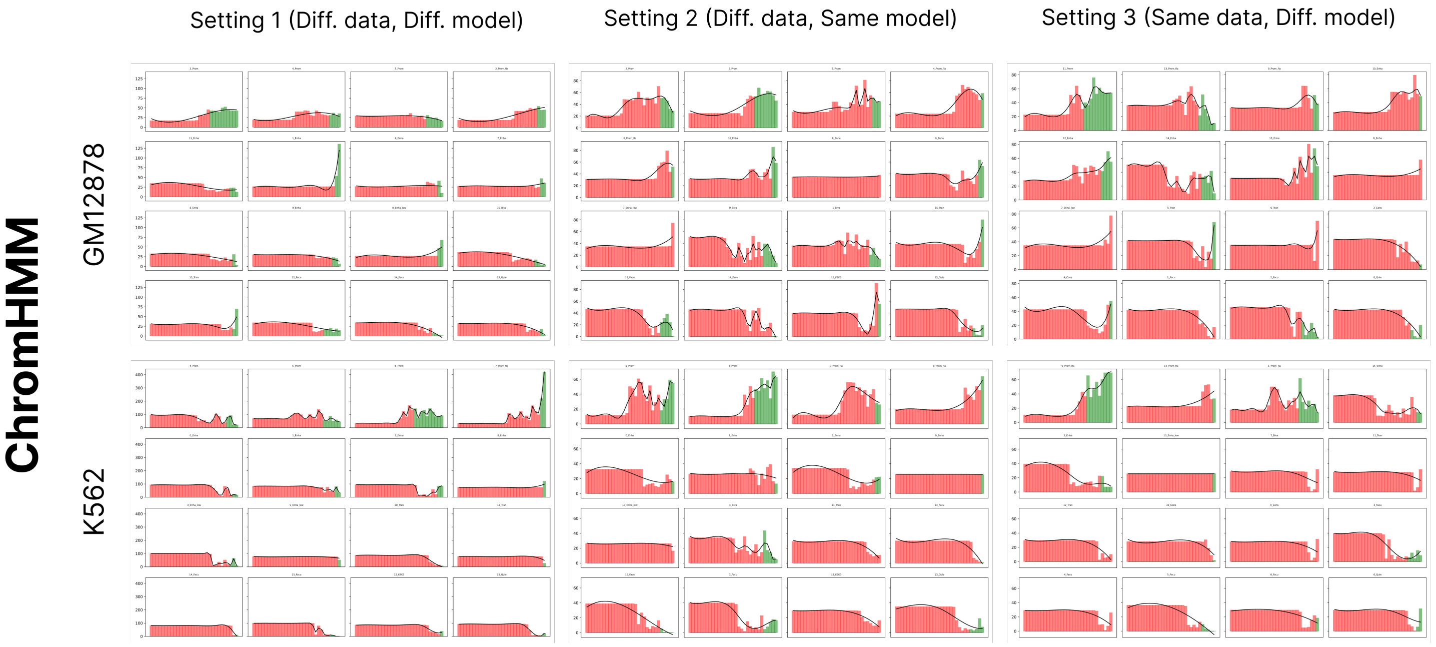

Figure 25: Mean expression level (TPM) around Transcription start sites as a function of posterior probability for ChromHMM runs in cell types GM12878 and K562. The horizontal axis represents rank of posterior probability, while the vertical axis shows TPM.

5.3.2 Segway

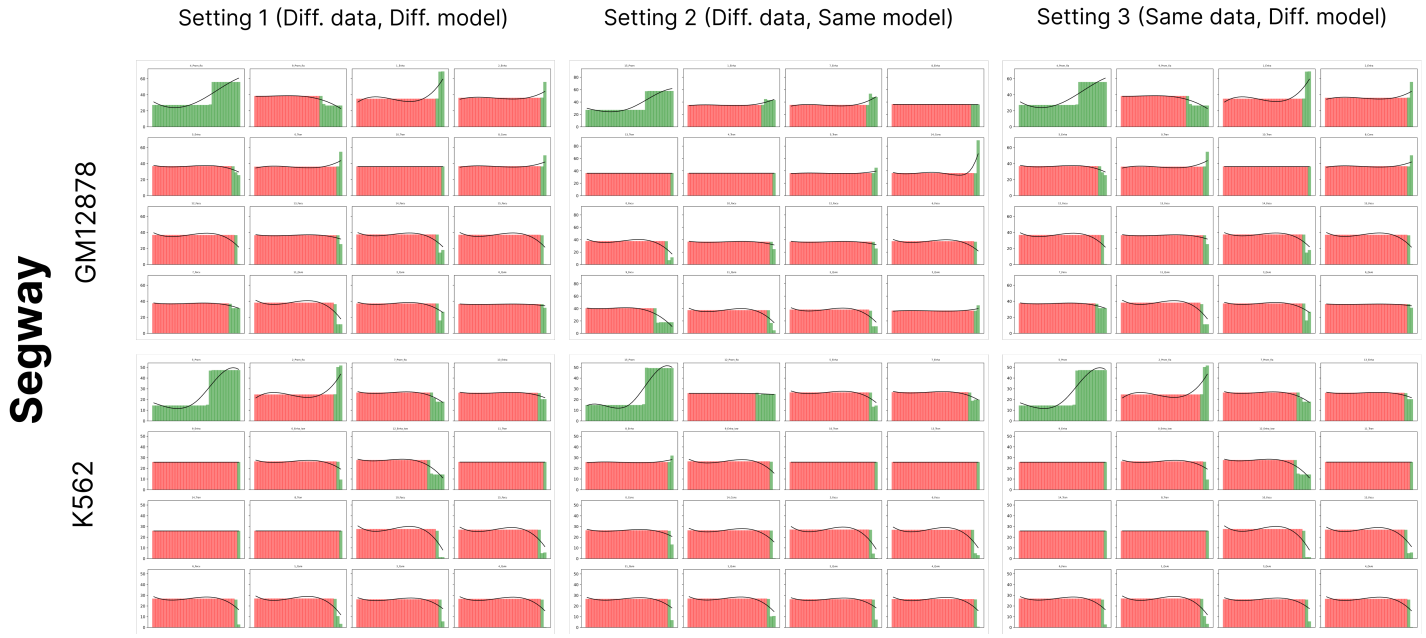

Figure 26: Mean expression level (TPM) around Transcription start sites as a function of posterior probability for Segway runs in cell types GM12878 and K562. The horizontal axis represents rank of posterior probability, while the vertical axis shows TPM.

5.4 TSS enrichment as a function of posterior probability

5.4.1 ChromHMM

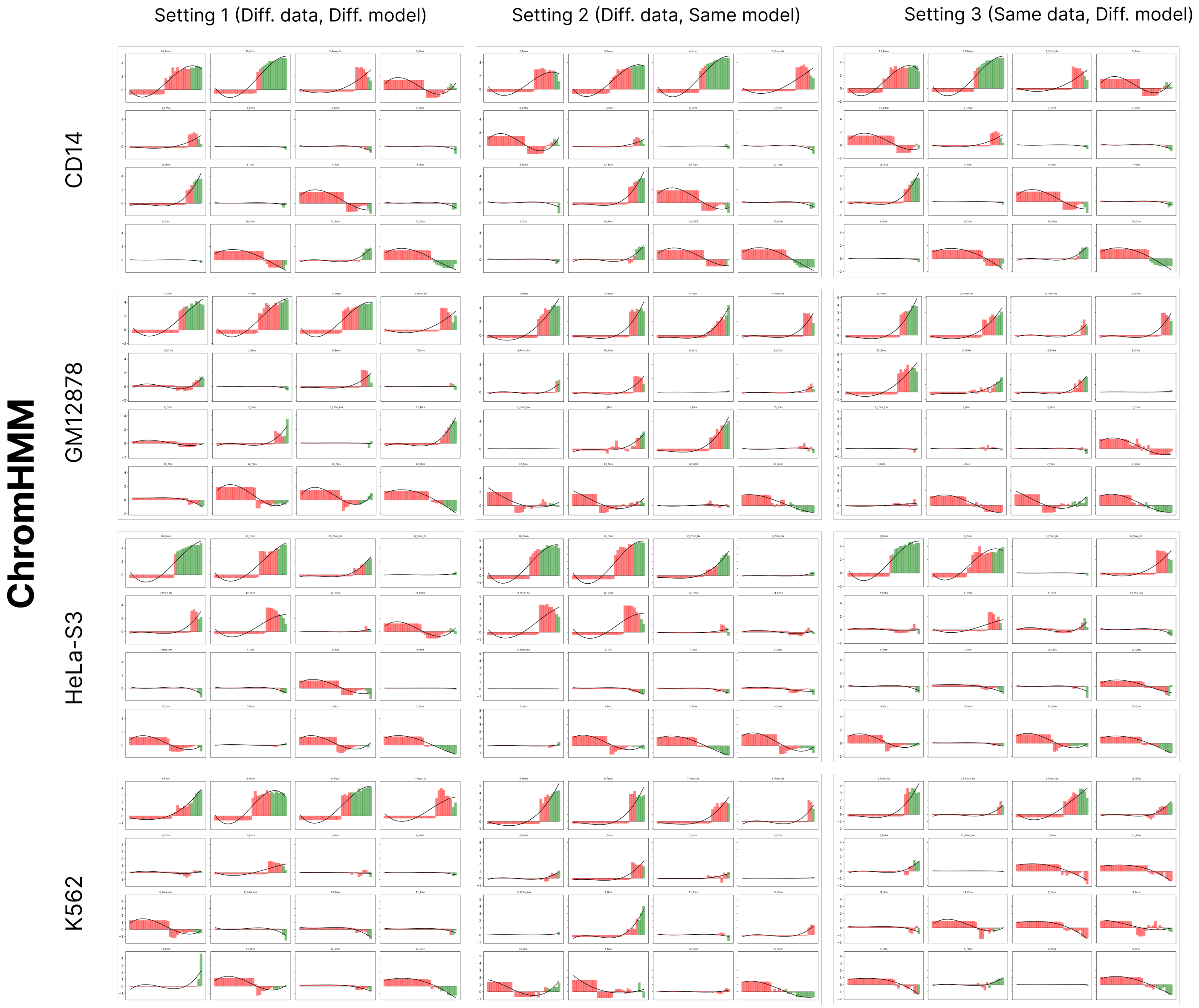

Figure 27: Enrichment of states around Transcription start sites as a function of posterior probability for ChromHMM runs in cell types GM12878 and K562. The horizontal axis represents rank of posterior probability, while the vertical axis shows TPM.

5.4.2 Segway

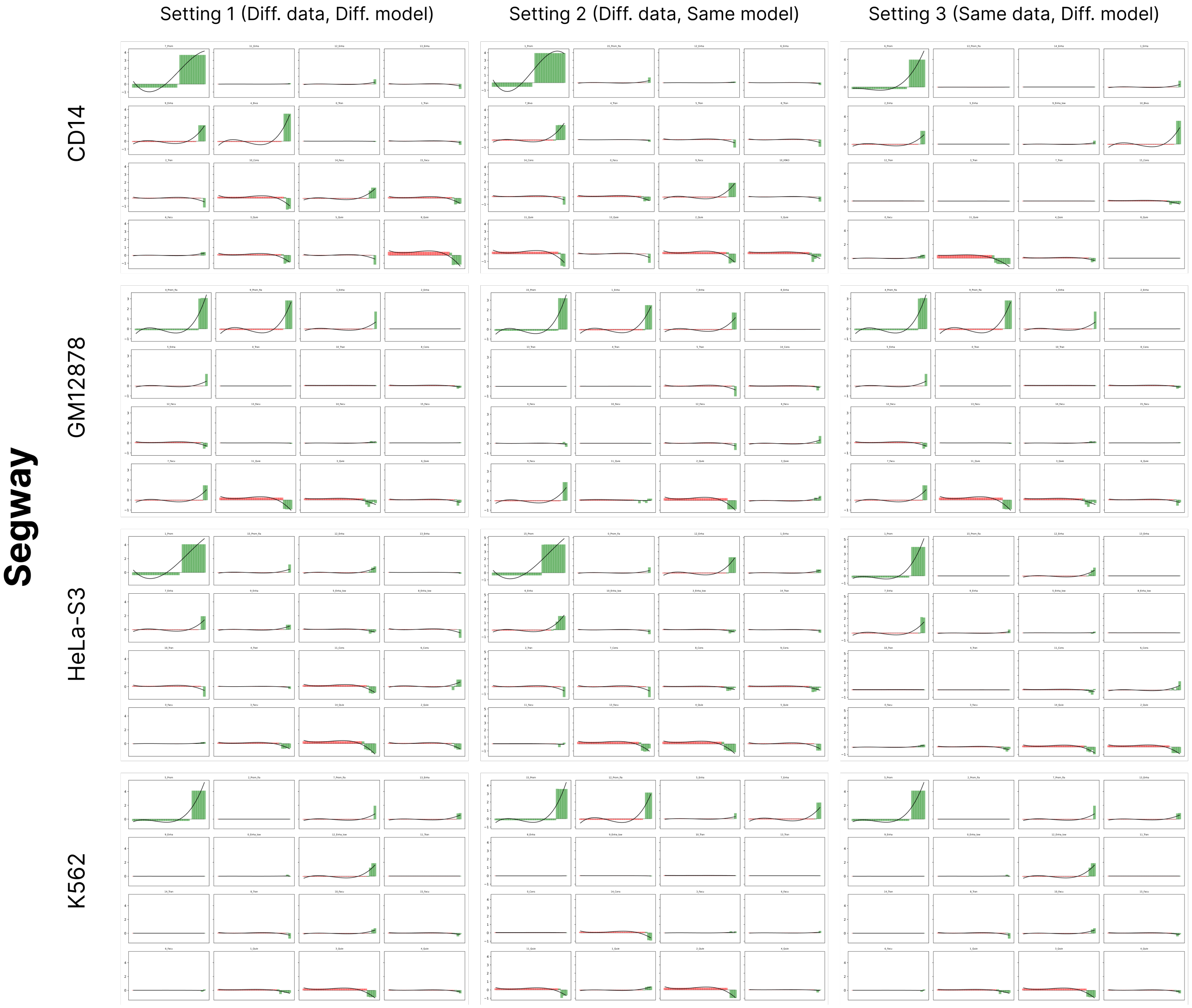

Figure 28: Enrichment of states around Transcription start sites as a function of posterior probability for Segway runs in cell types GM12878 and K562. The horizontal axis represents rank of posterior probability, while the vertical axis shows TPM.

6 Reproducibility analysis yields robust annotations

6.1 r-value

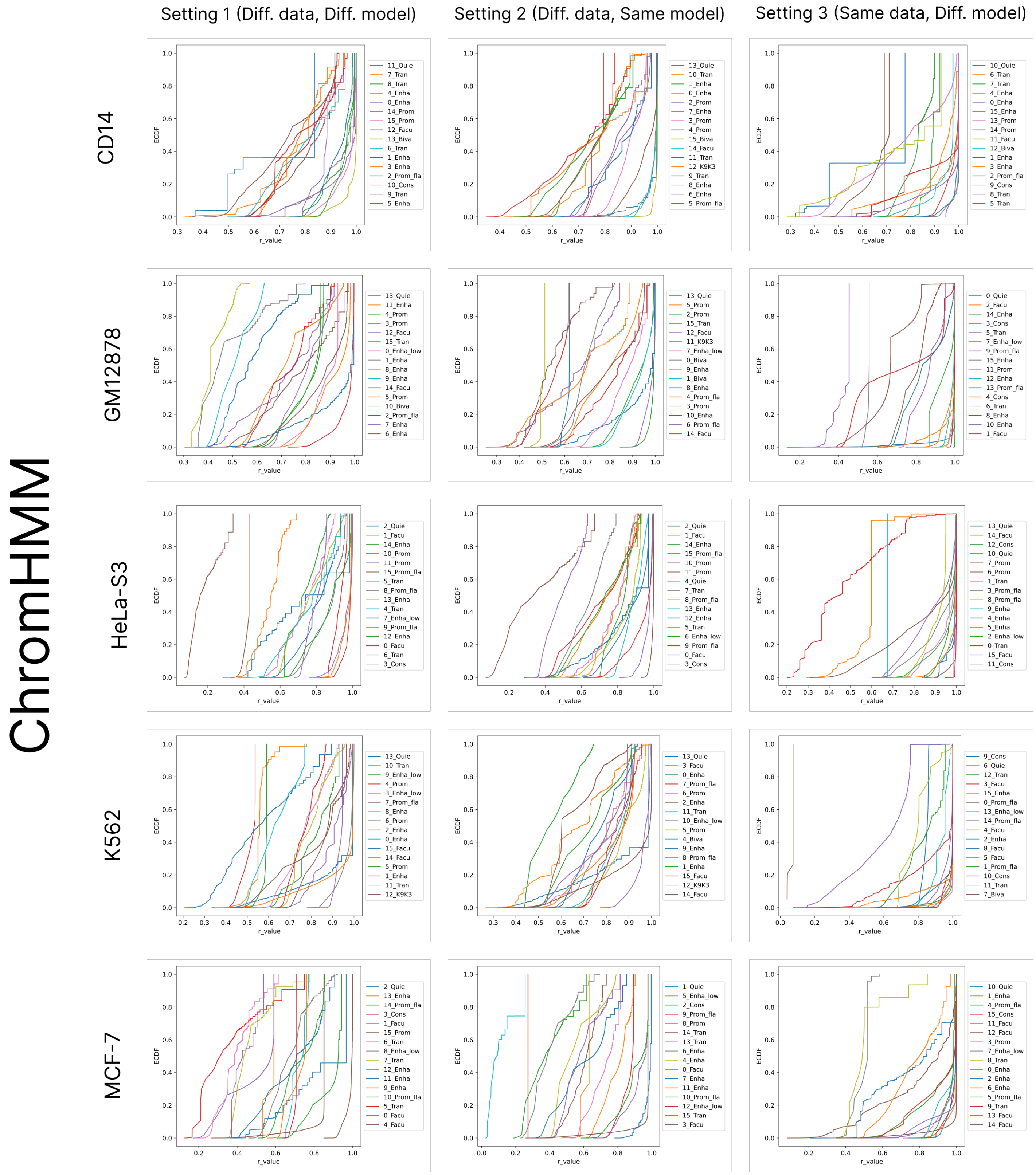

Figure 29: Empirical cumulative distribution function (ECDF) of r-value for all states in ChromHMM runs across five cell types (rows) and three settings of variability (columns).

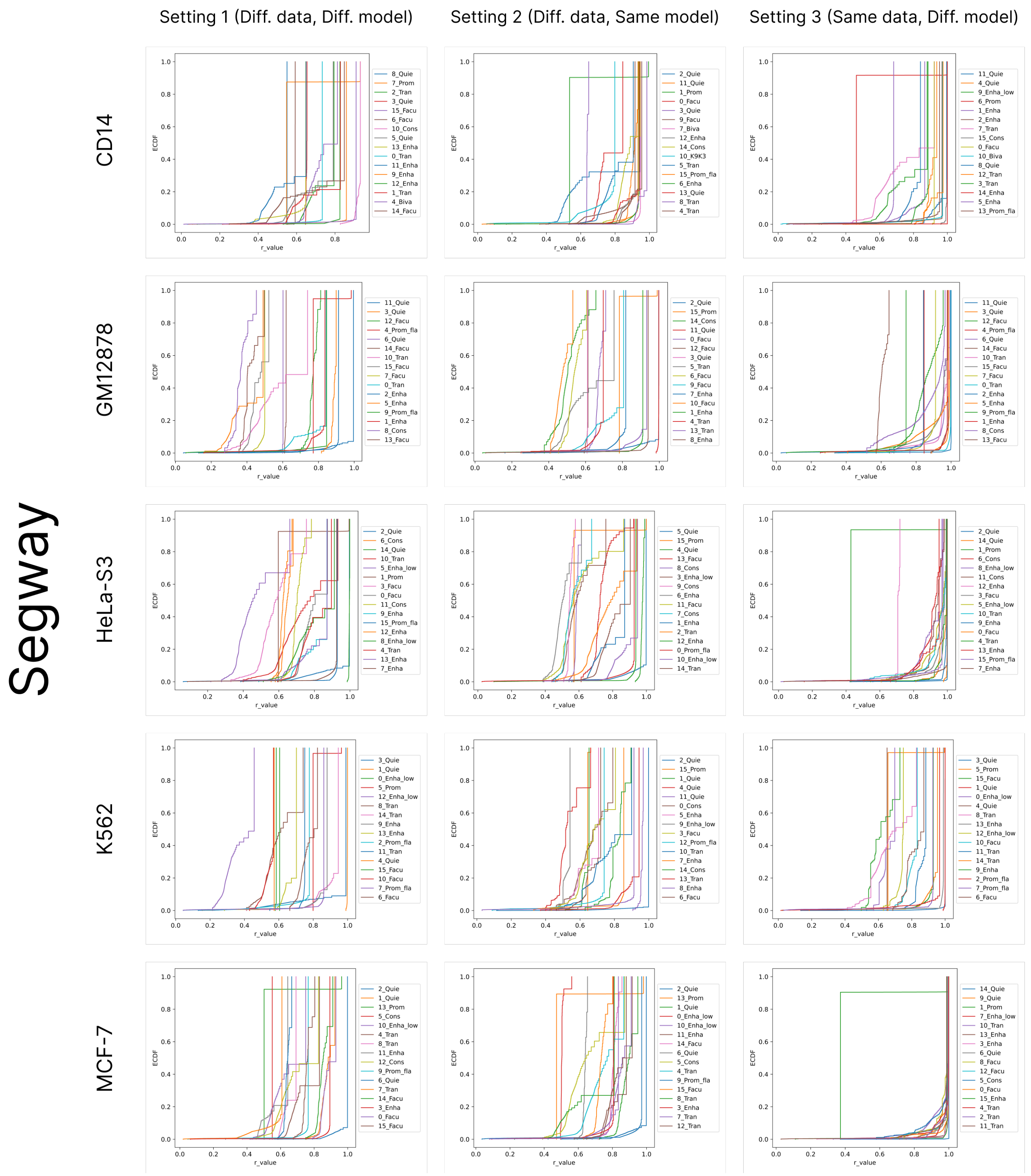

Figure 30: Empirical cumulative distribution function (ECDF) of r-value for all states in Segway runs across five cell types (rows) and three settings of variability (columns).

6.2 Fraction of confident genomic regions

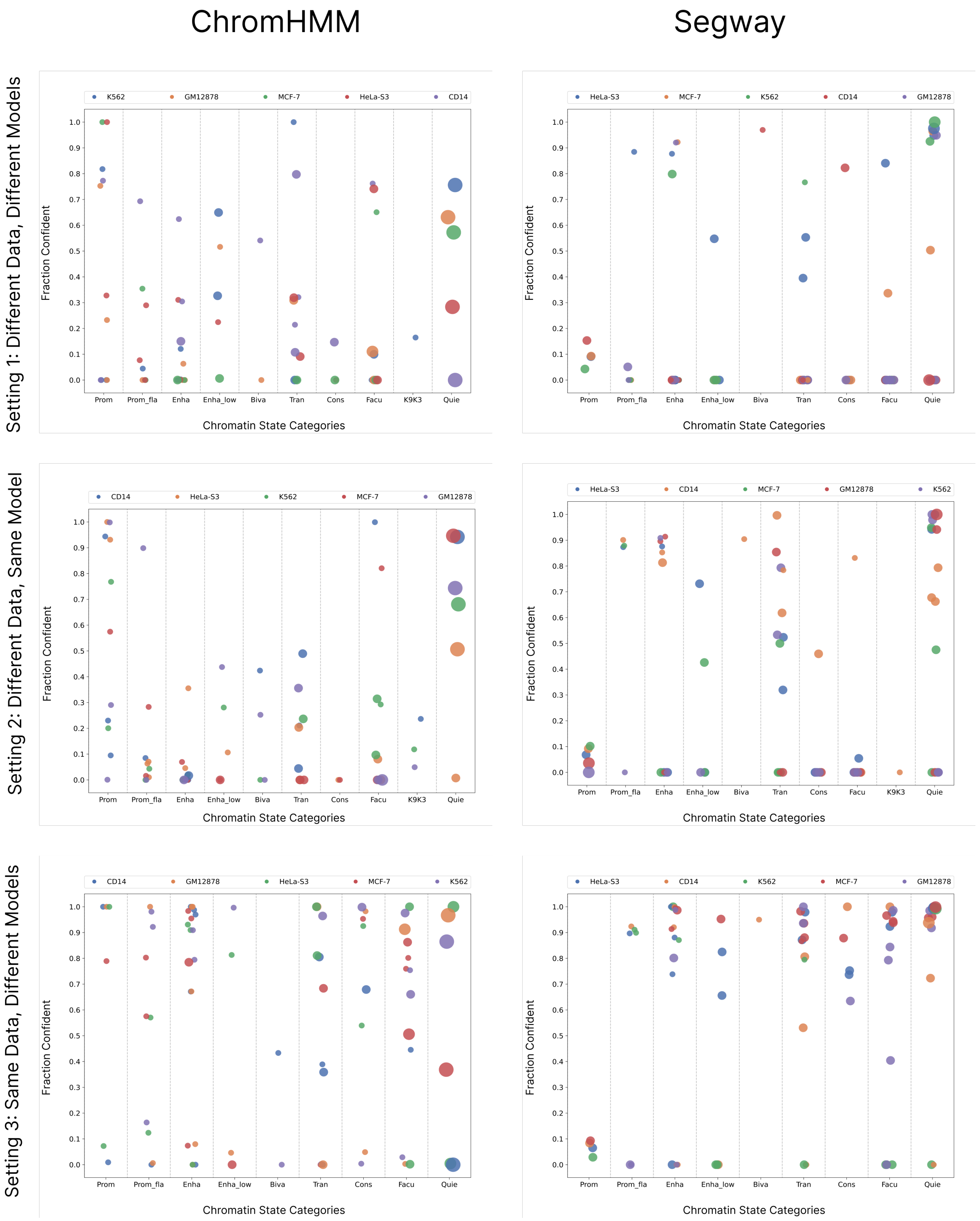

Figure 31: Fraction of confident genomic regions of various chromatin states categories identified in the ChromHMM (left column) and Segway (right column) annotation according to three settings of variability (rows) for five cell types. Each dot represents a chromatin state, with color denoting cell type and size proportional to genome coverage.

6.3 Expression as a function of r-value

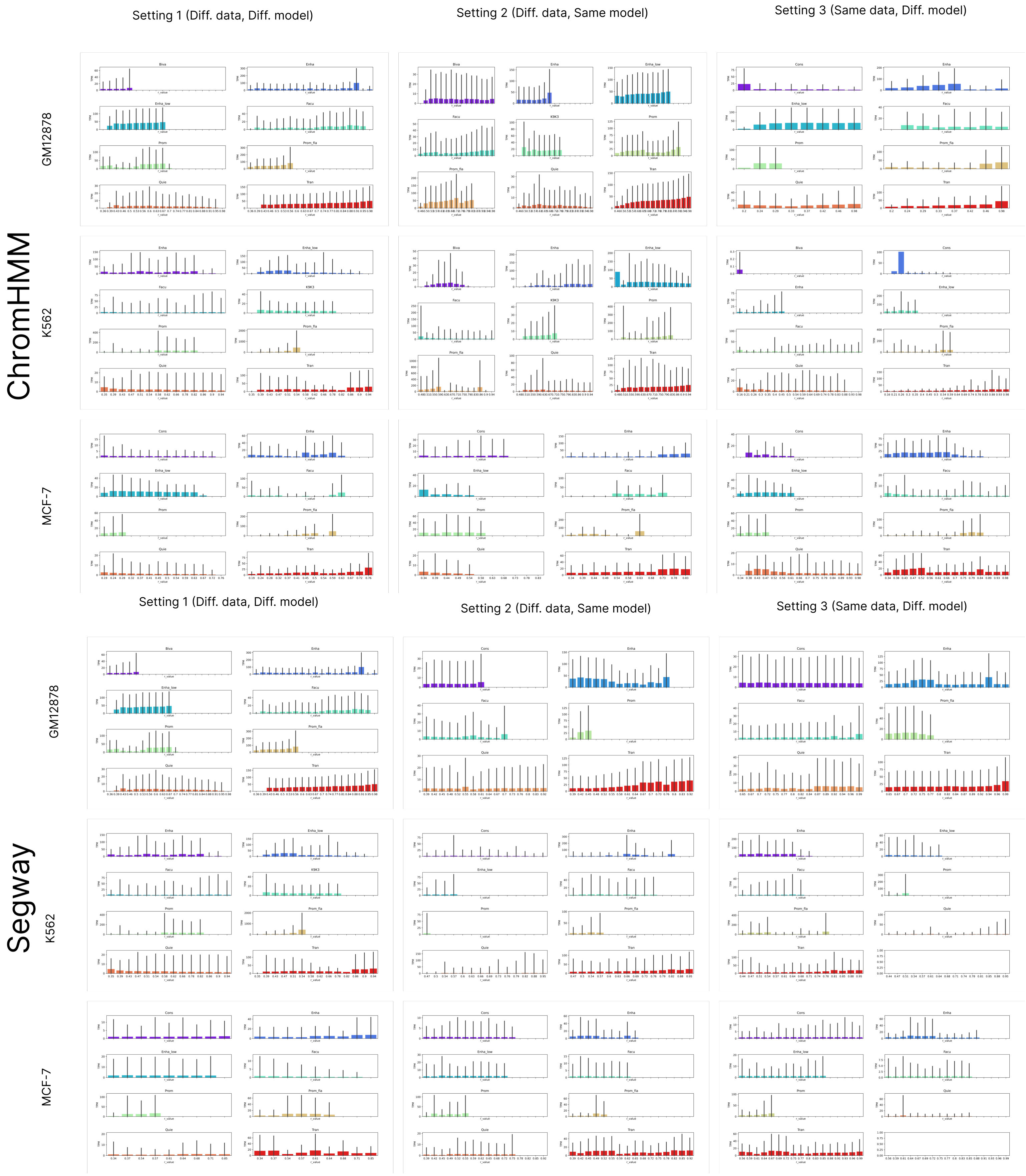

Figure 32: Bar plot representation of expression level (TPM) as a function of r-value chromatin state categories. Each bar signifies the mean TPM for each r-value bin, with error bars indicating the standard deviation.

6.4 Correlation of expression and r-value

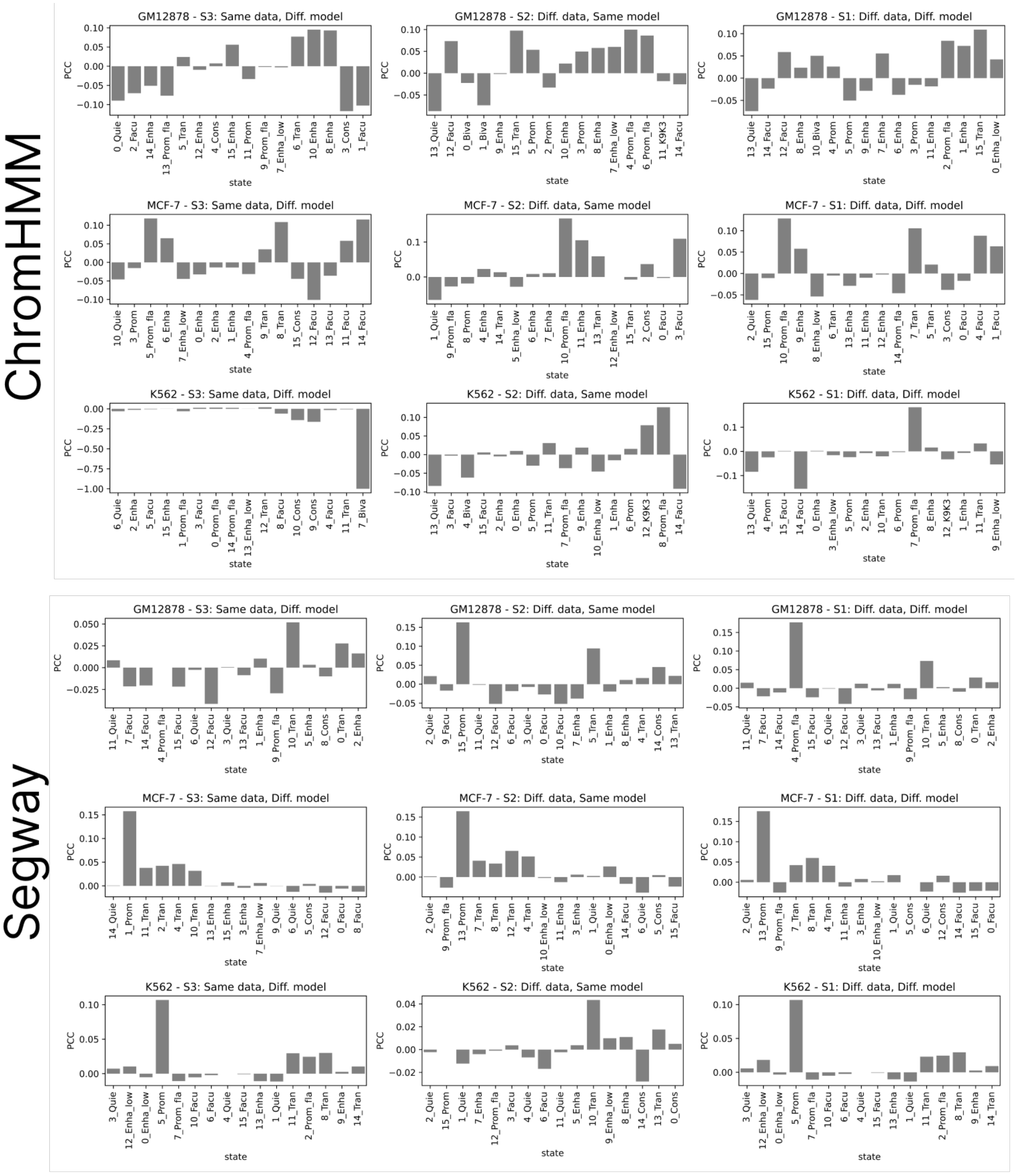

Figure 33: Figure illustrating Pearson correlation coefficient (PCC) of r-value and expression level (TPM) for different chromatin states.

6.5 Expression comparison of confident and non-confident transcribed states

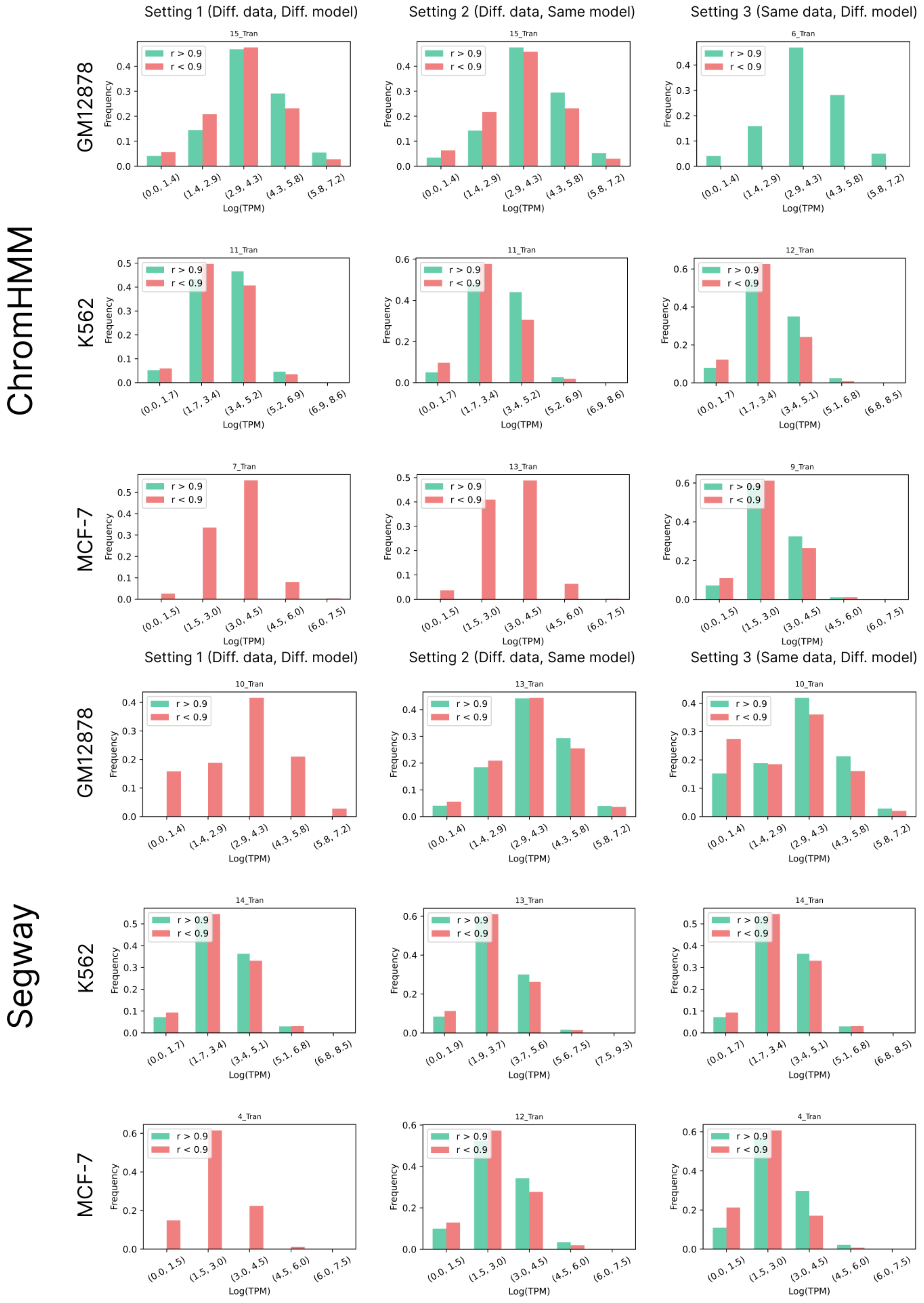

Figure 34: Histogram comparing the frequencies of log(TPM) values for confident ($r \geq 0.9$) and non-confident ($r < 0.9$) annotations of transcribed chromatin states. The x-axis represents ranges of log(TPM) with each pair of bars corresponding to confident (green) and non-confident (red) annotations respectively.

6.6 Reproducibility over segments

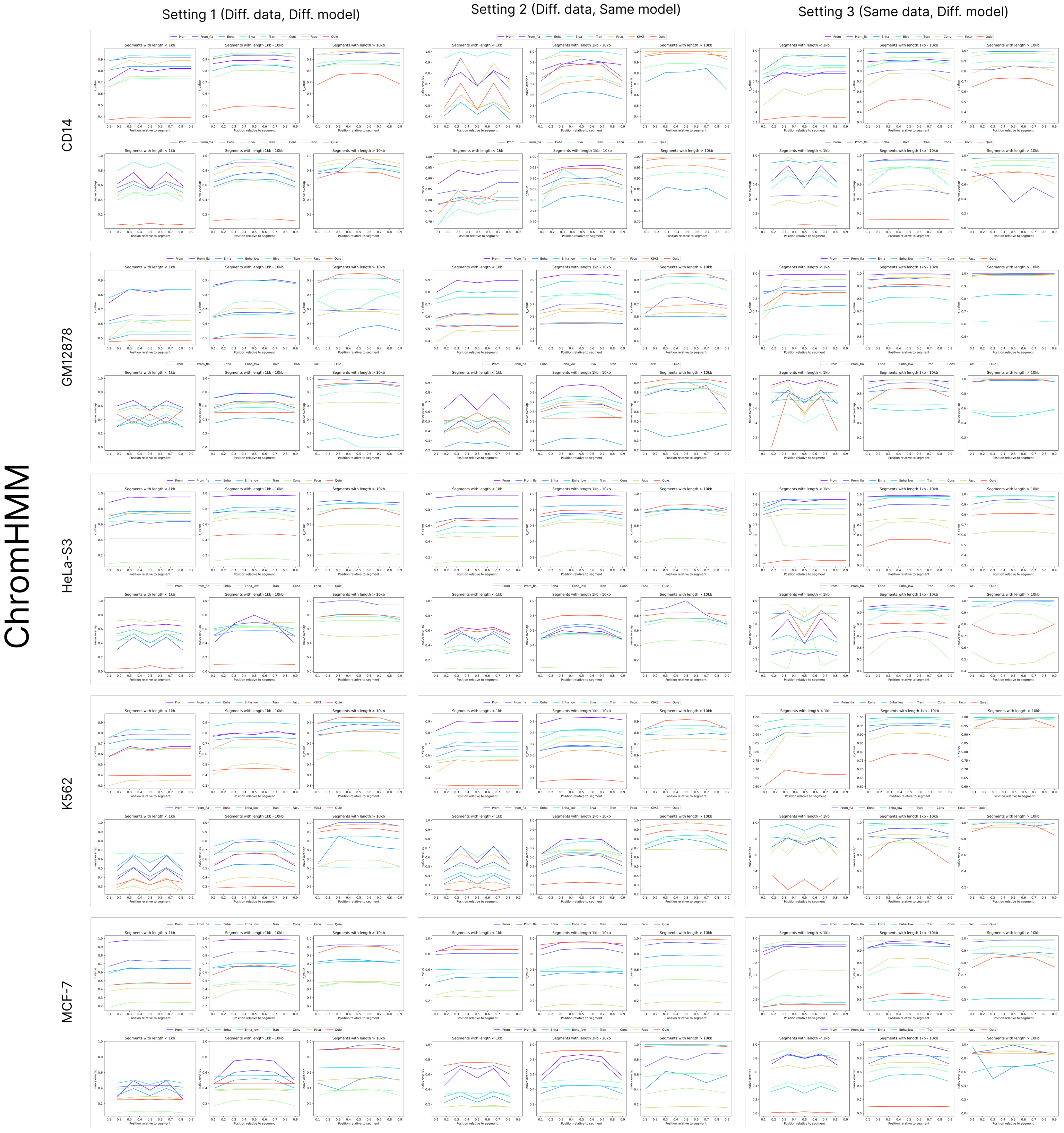

Figure 35: Figure illustrating reproducibility of ChromHMM annotations across segment lengths. For each pair of cell type and setting, top and bottom sub-figures show r-value and naive overlap distribution, respectively. Sub-figures from left to right represent segments < 1 kb, $1 - 10$kb, and ≥ 10kb. Colors denote different chromatin state categories.

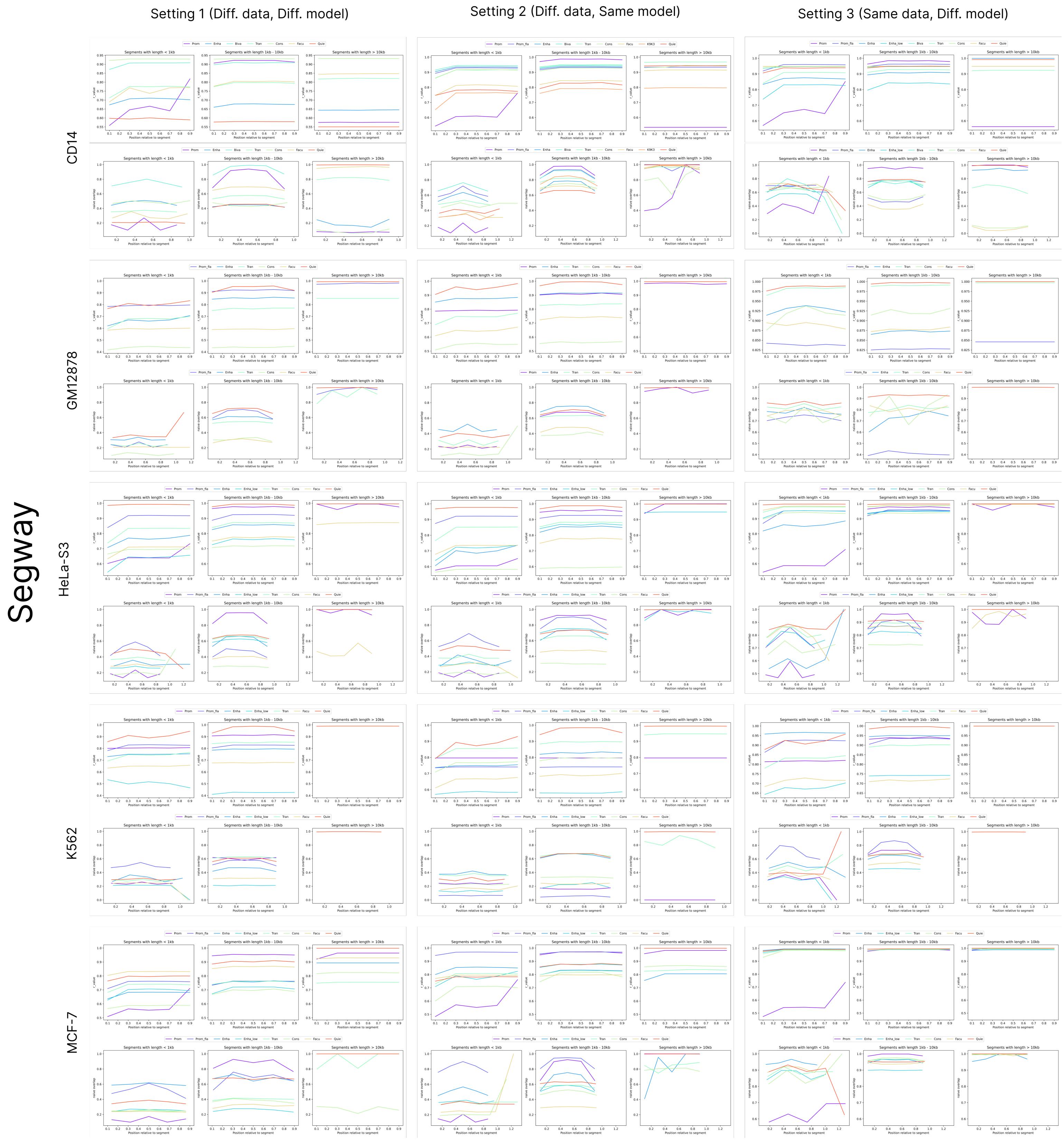

Figure 36: Figure illustrating reproducibility of Segway annotations across segment lengths. For each pair of cell type and setting, top and bottom sub-figures show r-value and naive overlap distribution, respectively. Sub-figures from left to right represent segments < 1 kb, $1-10$kb, and ≥ 10kb. Colors denote different chromatin state categories.

6.7 Distribution of r-value in open chromatin regions

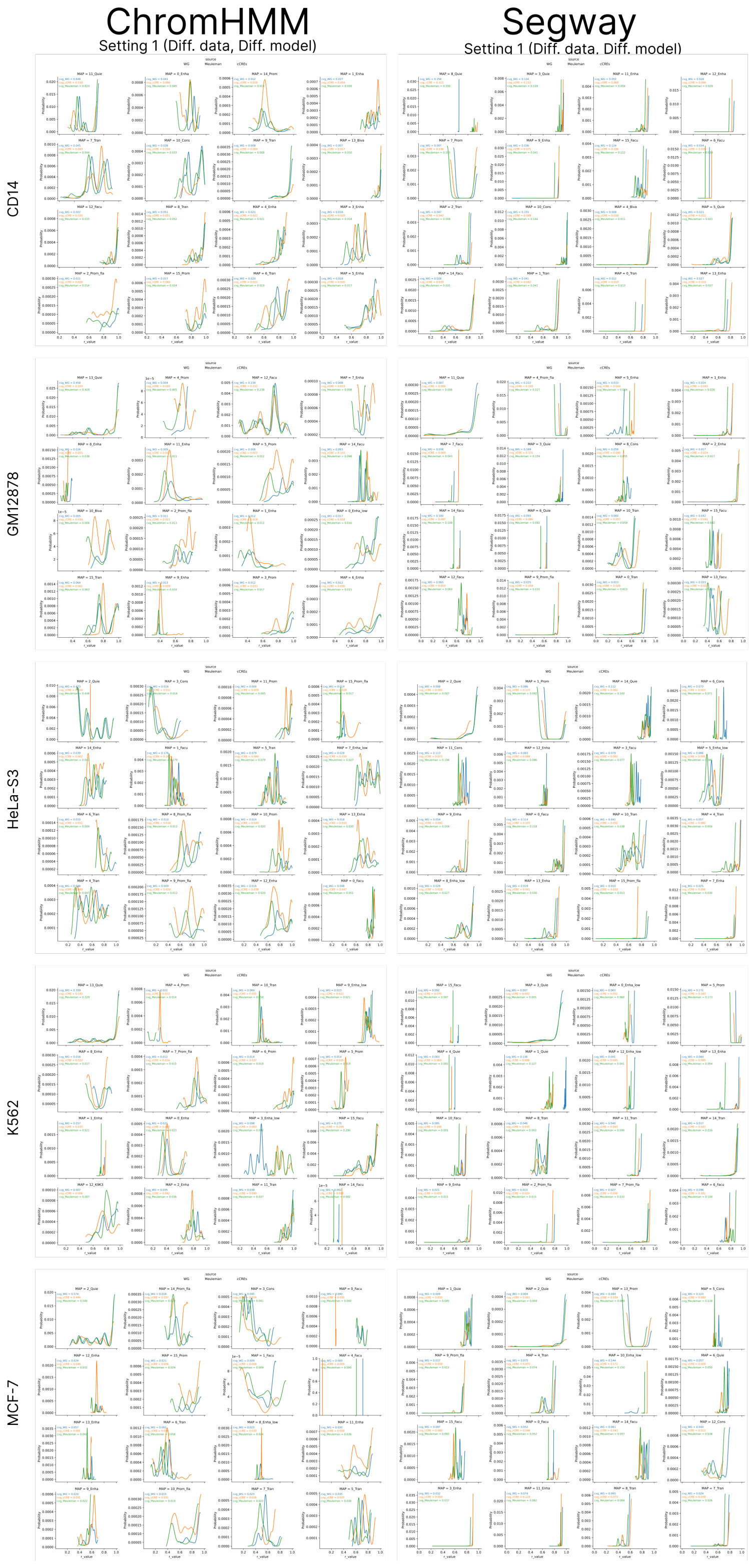

Figure 37: Figure comparing the distribution of r-values for different chromatin states (each sub-panel) in three scenarios: Whole Genome (WG - blue), SCREEN Candidate cis-regulatory Elements (cCRE - orange) [Moore et al., 2020], and Mueleman open chromatin regions [Meuleman et al., 2020] (green). The coverage of each chromatin state is indicated in the corresponding color. The area under each line represents the normalized frequency by corresponding genome coverage.

7 Comparison of Biologically Replicated ChIP-Seq Assays

7.1 CD14-positive-monocyte

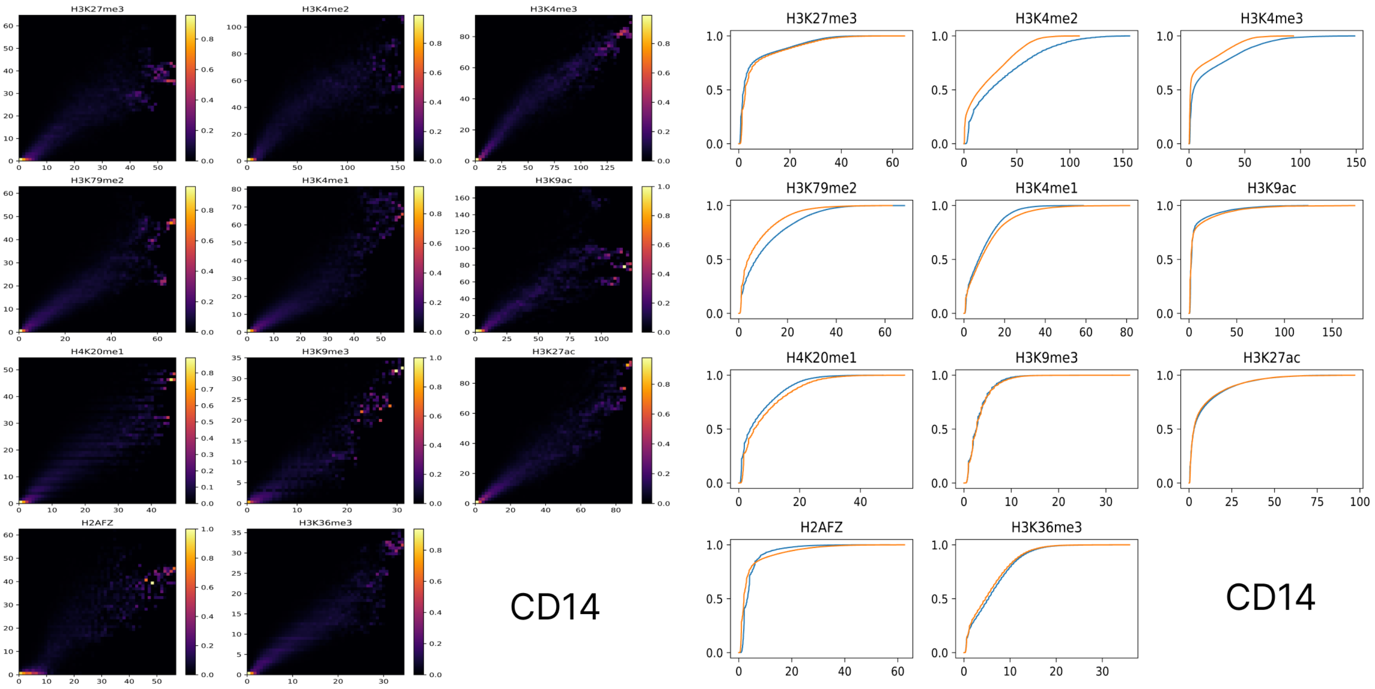

Figure 38: Heatmap of track values across two biological replicates for different histone modification tracks (left). On right, empirical cumulative distribution function (ECDF) of track values across two biological replicates for different histone modification tracks.

7.2 GM12878

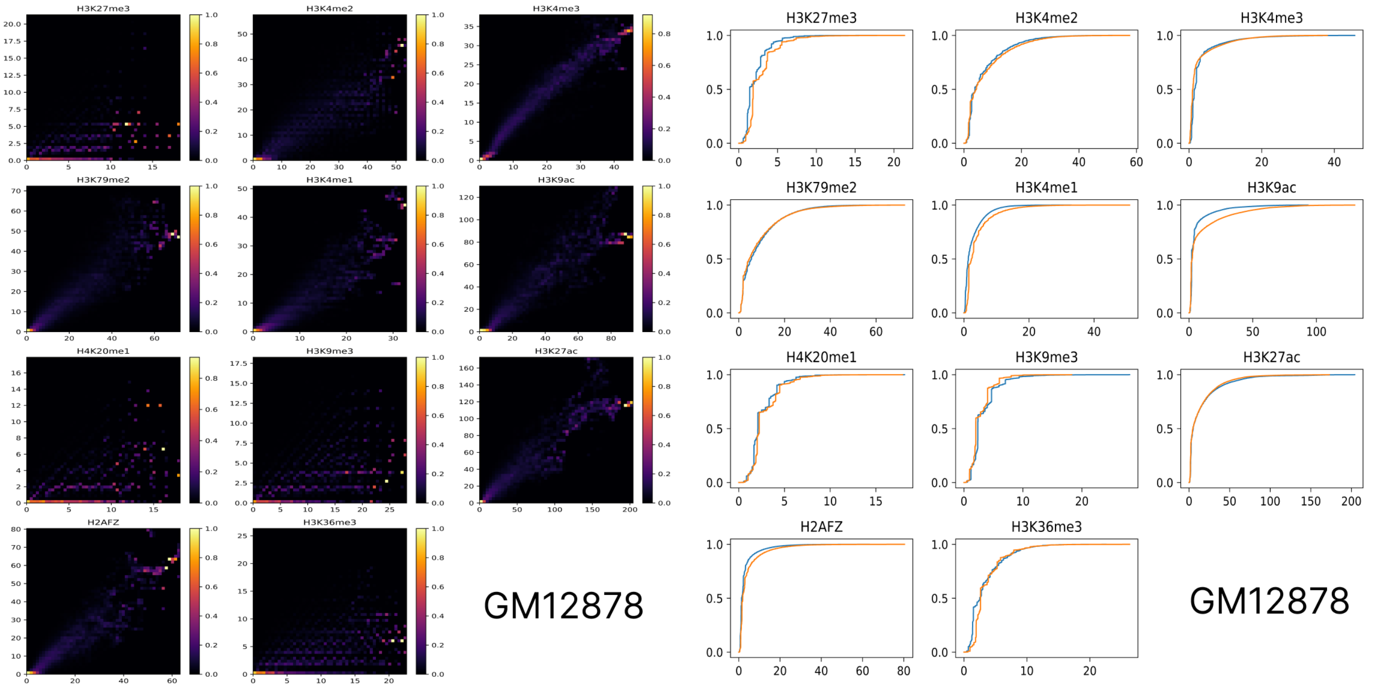

Figure 39: Heatmap of track values across two biological replicates for different histone modification tracks (left). On right, empirical cumulative distribution function (ECDF) of track values across two biological replicates for different histone modification tracks.

7.3 HeLa-S3

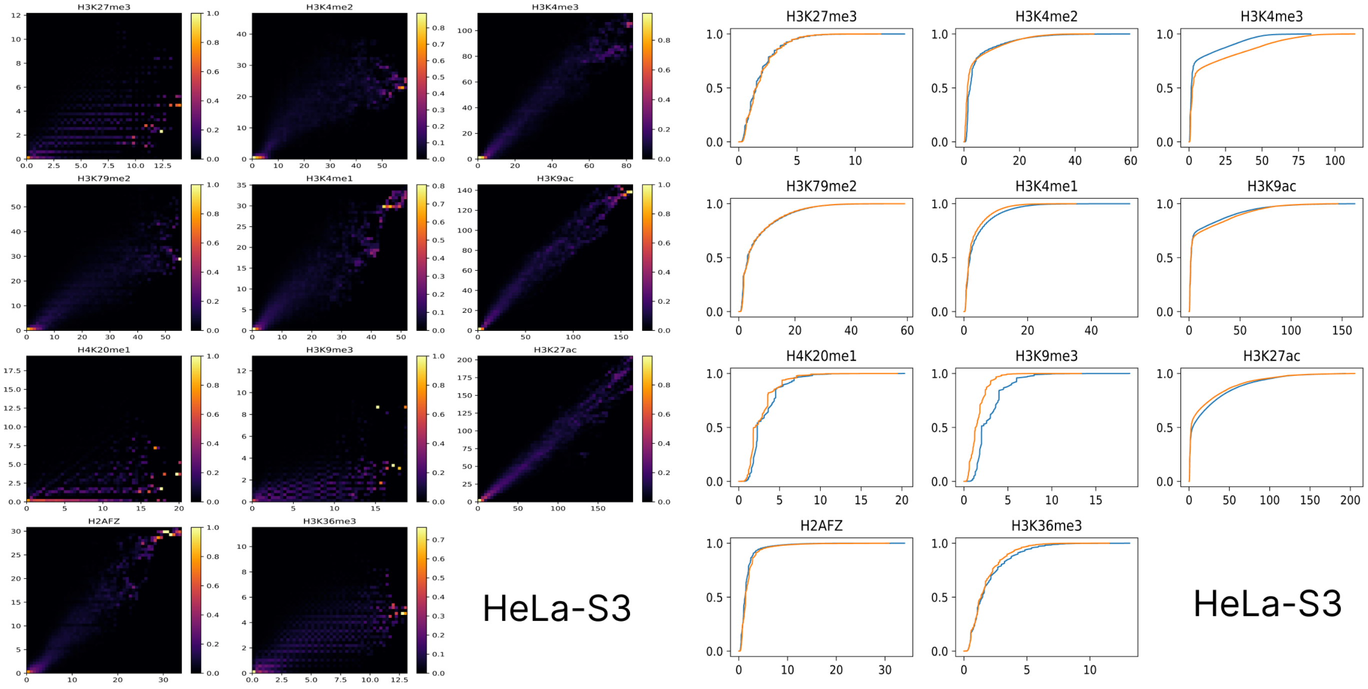

Figure 40: Heatmap of track values across two biological replicates for different histone modification tracks (left). On right, empirical cumulative distribution function (ECDF) of track values across two biological replicates for different histone modification tracks.

7.4 K562

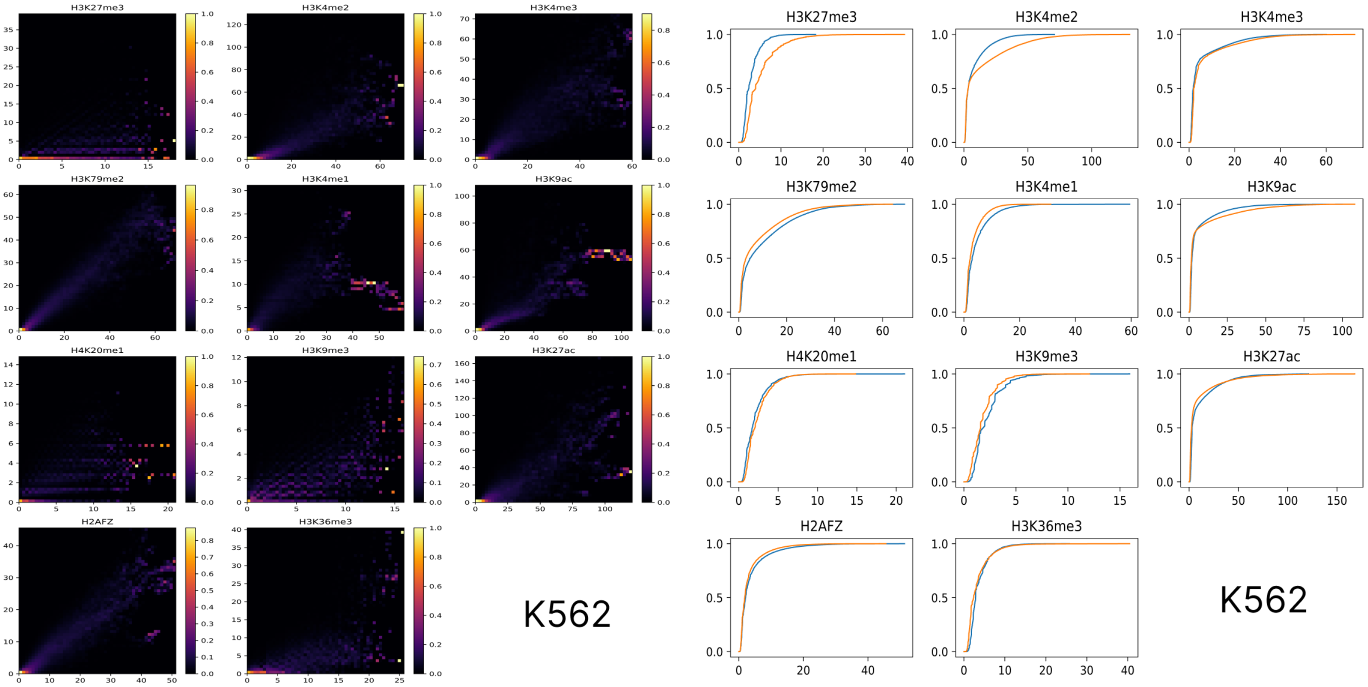

Figure 41: Heatmap of track values across two biological replicates for different histone modification tracks (left). On right, empirical cumulative distribution function (ECDF) of track values across two biological replicates for different histone modification tracks.

7.5 MCF-7

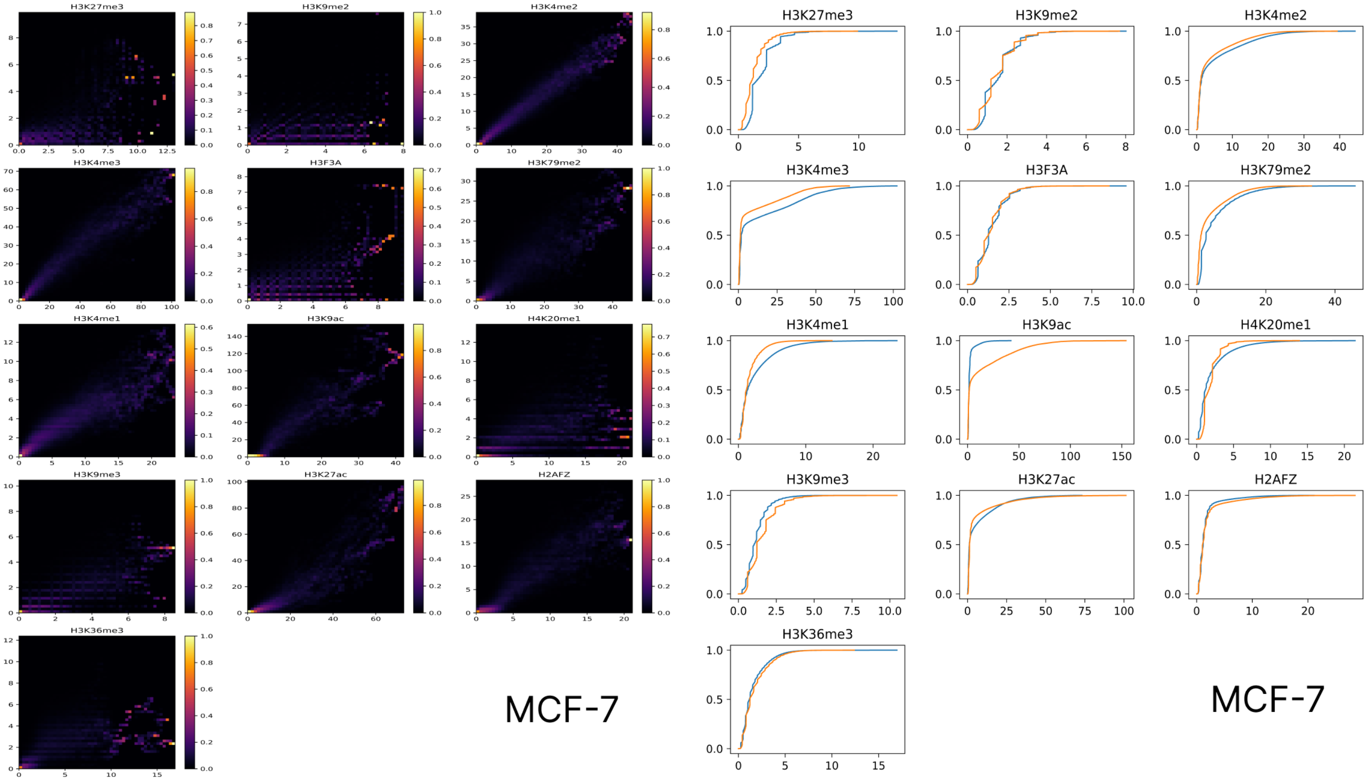

Figure 42: Heatmap of track values across two biological replicates for different histone modification tracks (left). On right, empirical cumulative distribution function (ECDF) of track values across two biological replicates for different histone modification tracks.

8 Data Summary

Table 2: This table contains the summary of data retrieved from ENCODE consortium [Consortium et al., 2012] and used to train both ChromHMM and Segway. For each experiment, two biosamples, each corresponding to an isogenic replicate, has been used. For each biosample, two files were retrieved, the aligned reads BAM file and the fold-change over control signal file respectively.

Cell type	Experiment	Biosample	Files
MCF-7	H3K27me3	ENCBS789UPK (rep. 1)	ENCFF413QYQ, ENCFF222KPM
MCF-7	H3K27me3	ENCBS967MVZ (rep. 2)	ENCFF744JQU, ENCFF070PLQ
MCF-7	H3K9me2	ENCBS789UPK (rep. 1)	ENCFF249TCH, ENCFF727FOP
MCF-7	H3K9me2	ENCBS967MVZ (rep. 2)	ENCFF418SFP, ENCFF727JSD
MCF-7	H3K4me2	ENCBS789UPK (rep. 1)	ENCFF134YPW, ENCFF095LXY
MCF-7	H3K4me2	ENCBS967MVZ (rep. 2)	ENCFF680ERD, ENCFF930AIB
MCF-7	H3K4me3	ENCBS789UPK (rep. 1)	ENCFF101TRI, ENCFF559CLH
MCF-7	H3K4me3	ENCBS967MVZ (rep. 2)	ENCFF374GQR, ENCFF763FZF
MCF-7	H3F3A	ENCBS789UPK (rep. 1)	ENCFF626ORM, ENCFF513VYY
MCF-7	H3F3A	ENCBS967MVZ (rep. 2)	ENCFF183HME, ENCFF820MMV
MCF-7	H3K79me2	ENCBS789UPK (rep. 1)	ENCFF312WJY, ENCFF921ILS
MCF-7	H3K79me2	ENCBS967MVZ (rep. 2)	ENCFF654NDM, ENCFF288DKP
MCF-7	H3K4me1	ENCBS789UPK (rep. 1)	ENCFF592EVS, ENCFF885FSN
MCF-7	H3K4me1	ENCBS967MVZ (rep. 2)	ENCFF748ISL, ENCFF943MKW
MCF-7	H3K9ac	ENCBS789UPK (rep. 1)	ENCFF571MVG, ENCFF967PEQ
MCF-7	H3K9ac	ENCBS967MVZ (rep. 2)	ENCFF403SOH, ENCFF699NOJ
MCF-7	H4K20me1	ENCBS789UPK (rep. 1)	ENCFF491EQT, ENCFF665VEZ
MCF-7	H4K20me1	ENCBS967MVZ (rep. 2)	ENCFF741HOA, ENCFF382BVG
MCF-7	H3K9me3	ENCBS789UPK (rep. 1)	ENCFF909MGB, ENCFF501WFD
MCF-7	H3K9me3	ENCBS967MVZ (rep. 2)	ENCFF923NDP, ENCFF587KXV
MCF-7	H3K27ac	ENCBS789UPK (rep. 1)	ENCFF785NUK, ENCFF395YPR
MCF-7	H3K27ac	ENCBS967MVZ (rep. 2)	ENCFF391BOB, ENCFF047PAZ
MCF-7	H2AFZ	ENCBS789UPK (rep. 1)	ENCFF597OEX, ENCFF269EZS
MCF-7	H2AFZ	ENCBS967MVZ (rep. 2)	ENCFF687JRM, ENCFF391YLE
MCF-7	H3K36me3	ENCBS789UPK (rep. 1)	ENCFF747AWB, ENCFF039TSW
MCF-7	H3K36me3	ENCBS967MVZ (rep. 2)	ENCFF551PNK, ENCFF781COV
GM12878	H3K27me3	ENCBS715VCP (rep. 1)	ENCFF633BHN, ENCFF119CAV
GM12878	H3K27me3	ENCBS830CIQ (rep. 2)	ENCFF565DCK, ENCFF736CNQ
GM12878	H3K4me2	ENCBS715VCP (rep. 1)	ENCFF008LEY, ENCFF028DRR
GM12878	H3K4me2	ENCBS830CIQ (rep. 2)	ENCFF081ODV, ENCFF922IYX
GM12878	H3K4me3	ENCBS715VCP (rep. 1)	ENCFF843BWY, ENCFF346GYG
GM12878	H3K4me3	ENCBS830CIQ (rep. 2)	ENCFF126HII, ENCFF874RRW
GM12878	H3K79me2	ENCBS715VCP (rep. 1)	ENCFF465NXQ, ENCFF567RPY
GM12878	H3K79me2	ENCBS830CIQ (rep. 2)	ENCFF967CHL, ENCFF595CSY
GM12878	H3K4me1	ENCBS715VCP (rep. 1)	ENCFF047NLO, ENCFF785YET
GM12878	H3K4me1	ENCBS830CIQ (rep. 2)	ENCFF385FLM, ENCFF721OOX
GM12878	H3K9ac	ENCBS715VCP (rep. 1)	ENCFF729HQN, ENCFF609XKG
GM12878	H3K9ac	ENCBS830CIQ (rep. 2)	ENCFF405REQ, ENCFF404TNY
GM12878	H4K20me1	ENCBS715VCP (rep. 1)	ENCFF132EDT, ENCFF150LOV
GM12878	H4K20me1	ENCBS830CIQ (rep. 2)	ENCFF334NVA, ENCFF069ZOX
GM12878	H3K9me3	ENCBS715VCP (rep. 1)	ENCFF306ENK, ENCFF698SKV
GM12878	H3K9me3	ENCBS830CIQ (rep. 2)	ENCFF889UPU, ENCFF856REE
GM12878	H3K27ac	ENCBS715VCP (rep. 1)	ENCFF269GKF, ENCFF458CRP
GM12878	H3K27ac	ENCBS830CIQ (rep. 2)	ENCFF201OHW, ENCFF716VWO
GM12878	H2AFZ	ENCBS715VCP (rep. 1)	ENCFF703AIM, ENCFF477ZQM
GM12878	H2AFZ	ENCBS830CIQ (rep. 2)	ENCFF256UUQ, ENCFF553GSY
GM12878	H3K36me3	ENCBS715VCP (rep. 1)	ENCFF353YPB, ENCFF269OIU
GM12878	H3K36me3	ENCBS830CIQ (rep. 2)	ENCFF677MAG, ENCFF831NQV
K562	H3K27me3	ENCBS639AAA (rep. 1)	ENCFF392ZKG, ENCFF585RVP
K562	H3K27me3	ENCBS674MPN (rep. 2)	ENCFF905CZD, ENCFF679YZC

K562	H3K4me2	ENCBS639AAA (rep. 1)	ENCFF446FUS, ENCFF229IJV
K562	H3K4me2	ENCBS674MPN (rep. 2)	ENCFF583GSH, ENCFF386XDJ
K562	H3K4me3	ENCBS639AAA (rep. 1)	ENCFF564SVK, ENCFF545JLB
K562	H3K4me3	ENCBS674MPN (rep. 2)	ENCFF685PPQ, ENCFF617VRS
K562	H3K79me2	ENCBS639AAA (rep. 1)	ENCFF465UWC, ENCFF952GNK
K562	H3K79me2	ENCBS674MPN (rep. 2)	ENCFF863HNL, ENCFF080CBF
K562	H3K4me1	ENCBS639AAA (rep. 1)	ENCFF352HXD, ENCFF490RQD
K562	H3K4me1	ENCBS674MPN (rep. 2)	ENCFF415GHS, ENCFF149FZG
K562	H3K9ac	ENCBS639AAA (rep. 1)	ENCFF149MXA, ENCFF236VCK
K562	H3K9ac	ENCBS674MPN (rep. 2)	ENCFF505NMT, ENCFF650QTM
K562	H4K20me1	ENCBS639AAA (rep. 1)	ENCFF255QRL, ENCFF091XLR
K562	H4K20me1	ENCBS674MPN (rep. 2)	ENCFF923YUN, ENCFF006AYV
K562	H3K9me3	ENCBS639AAA (rep. 1)	ENCFF104THG, ENCFF103IHG
K562	H3K9me3	ENCBS674MPN (rep. 2)	ENCFF155UQU, ENCFF600IBI
K562	H3K27ac	ENCBS639AAA (rep. 1)	ENCFF121RHF, ENCFF977KGH
K562	H3K27ac	ENCBS674MPN (rep. 2)	ENCFF907MNY, ENCFF745TSK
K562	H2AFZ	ENCBS639AAA (rep. 1)	ENCFF874SMO, ENCFF242WQK
K562	H2AFZ	ENCBS674MPN (rep. 2)	ENCFF007YZT, ENCFF964BCA
K562	H3K36me3	ENCBS639AAA (rep. 1)	ENCFF880HKV, ENCFF782UNG
K562	H3K36me3	ENCBS674MPN (rep. 2)	ENCFF272JVI, ENCFF860PQP
CD14+ monocyte	H3K27me3	ENCBS188BKX (rep. 1)	ENCFF630ANH, ENCFF840DRQ
CD14+ monocyte	H3K27me3	ENCBS865RXK (rep. 2)	ENCFF785GAD, ENCFF586PRJ
CD14+ monocyte	H3K4me2	ENCBS188BKX (rep. 1)	ENCFF591CTO, ENCFF572NHK
CD14+ monocyte	H3K4me2	ENCBS865RXK (rep. 2)	ENCFF384IVB, ENCFF031HMX
CD14+ monocyte	H3K4me3	ENCBS188BKX (rep. 1)	ENCFF640GCK, ENCFF367ZHA
CD14+ monocyte	H3K4me3	ENCBS865RXK (rep. 2)	ENCFF144PKE, ENCFF439PJT
CD14+ monocyte	H3K79me2	ENCBS188BKX (rep. 1)	ENCFF463WUU, ENCFF464AAD
CD14+ monocyte	H3K79me2	ENCBS865RXK (rep. 2)	ENCFF159LCH, ENCFF451RBS
CD14+ monocyte	H3K4me1	ENCBS188BKX (rep. 1)	ENCFF573MNS, ENCFF281FSL
CD14+ monocyte	H3K4me1	ENCBS865RXK (rep. 2)	ENCFF141KHX, ENCFF669AVC
CD14+ monocyte	H3K9ac	ENCBS188BKX (rep. 1)	ENCFF775XVG, ENCFF835QUH
CD14+ monocyte	H3K9ac	ENCBS865RXK (rep. 2)	ENCFF326KCT, ENCFF627PKL
CD14+ monocyte	H4K20me1	ENCBS865RXK (rep. 2)	ENCFF238ZAC, ENCFF938RSM
CD14+ monocyte	H4K20me1	ENCBS188BKX (rep. 1)	ENCFF899MAF, ENCFF657KKL
CD14+ monocyte	H3K9me3	ENCBS188BKX (rep. 1)	ENCFF537BKE, ENCFF264DFY
CD14+ monocyte	H3K9me3	ENCBS865RXK (rep. 2)	ENCFF168UCU, ENCFF726FHO
CD14+ monocyte	H3K27ac	ENCBS188BKX (rep. 1)	ENCFF014BCD, ENCFF078DZN
CD14+ monocyte	H3K27ac	ENCBS865RXK (rep. 2)	ENCFF872QFO, ENCFF948MET
CD14+ monocyte	H2AFZ	ENCBS188BKX (rep. 1)	ENCFF891JRY, ENCFF834IMH
CD14+ monocyte	H2AFZ	ENCBS865RXK (rep. 2)	ENCFF992JKA, ENCFF175WGJ
CD14+ monocyte	H3K36me3	ENCBS188BKX (rep. 1)	ENCFF629ODR, ENCFF494ICL
CD14+ monocyte	H3K36me3	ENCBS865RXK (rep. 2)	ENCFF150SGV, ENCFF554VRE
HeLa-S3	H3K27me3	ENCBS075PNA (rep. 1)	ENCFF299WXR, ENCFF971CNR
HeLa-S3	H3K27me3	ENCBS655ARO (rep. 2)	ENCFF620CCL, ENCFF837NDL
HeLa-S3	H3K4me2	ENCBS075PNA (rep. 1)	ENCFF622IIB, ENCFF375VEB
HeLa-S3	H3K4me2	ENCBS655ARO (rep. 2)	ENCFF706FSX, ENCFF146XSE
HeLa-S3	H3K4me3	ENCBS075PNA (rep. 1)	ENCFF300IWJ, ENCFF532TVQ
HeLa-S3	H3K4me3	ENCBS655ARO (rep. 2)	ENCFF766LEF, ENCFF933NWW
HeLa-S3	H3K79me2	ENCBS075PNA (rep. 1)	ENCFF616FAT, ENCFF976XPV
HeLa-S3	H3K79me2	ENCBS655ARO (rep. 2)	ENCFF656YCM, ENCFF418AXA
HeLa-S3	H3K4me1	ENCBS075PNA (rep. 1)	ENCFF826OLG, ENCFF554WAD
HeLa-S3	H3K4me1	ENCBS655ARO (rep. 2)	ENCFF712AAP, ENCFF257YVT
HeLa-S3	H3K9ac	ENCBS075PNA (rep. 1)	ENCFF113FCH, ENCFF753SWH
HeLa-S3	H3K9ac	ENCBS655ARO (rep. 2)	ENCFF596XSW, ENCFF995MCG
HeLa-S3	H4K20me1	ENCBS075PNA (rep. 1)	ENCFF278FTJ, ENCFF776IYG
HeLa-S3	H4K20me1	ENCBS655ARO (rep. 2)	ENCFF310NWI, ENCFF002OOC
HeLa-S3	H3K9me3	ENCBS075PNA (rep. 1)	ENCFF183EDU, ENCFF746UJB
HeLa-S3	H3K9me3	ENCBS655ARO (rep. 2)	ENCFF520XFF, ENCFF629AAP
HeLa-S3	H3K27ac	ENCBS075PNA (rep. 1)	ENCFF609ZAE, ENCFF010QGR
HeLa-S3	H3K27ac	ENCBS655ARO (rep. 2)	ENCFF711QAI, ENCFF855XCJ

HeLa-S3	H2AFZ	ENCBS075PNA (rep. 1)	ENCFF650RBB, ENCFF423XMI
HeLa-S3	H2AFZ	ENCBS655ARO (rep. 2)	ENCFF822OCY, ENCFF104TOM
HeLa-S3	H3K36me3	ENCBS075PNA (rep. 1)	ENCFF317FFS, ENCFF305UYI
HeLa-S3	H3K36me3	ENCBS655ARO (rep. 2)	ENCFF008RRT, ENCFF870FDA

References

[Chan et al., 2018] Chan, R. C., Libbrecht, M. W., Roberts, E. G., Bilmes, J. A., Noble, W. S., and Hoffman, M. M. (2018). Segway 2.0: Gaussian mixture models and minibatch training. *Bioinformatics*, 34(4):669–671.

[Consortium et al., 2012] Consortium, E. P. et al. (2012). An integrated encyclopedia of dna elements in the human genome. *Nature*, 489(7414):57.

[Ernst and Kellis, 2012] Ernst, J. and Kellis, M. (2012). Chromhmm: automating chromatin-state discovery and characterization. *Nature methods*, 9(3):215–216.

[Hoffman et al., 2010] Hoffman, M. M., Buske, O. J., and Noble, W. S. (2010). The genomedata format for storing large-scale functional genomics data. *Bioinformatics*, 26(11):1458–1459.

[Hoffman et al., 2012] Hoffman, M. M., Buske, O. J., Wang, J., Weng, Z., Bilmes, J. A., and Noble, W. S. (2012). Unsupervised pattern discovery in human chromatin structure through genomic segmentation. *Nature methods*, 9(5):473–476.

[Hoffman et al., 2013] Hoffman, M. M., Ernst, J., Wilder, S. P., Kundaje, A., Harris, R. S., Libbrecht, M., Giardine, B., Ellenbogen, P. M., Bilmes, J. A., Birney, E., et al. (2013). Integrative annotation of chromatin elements from encode data. *Nucleic acids research*, 41(2):827–841.

[Libbrecht et al., 2019] Libbrecht, M. W., Rodriguez, O. L., Weng, Z., Bilmes, J. A., Hoffman, M. M., and Noble, W. S. (2019). A unified encyclopedia of human functional dna elements through fully automated annotation of 164 human cell types. *Genome biology*, 20(1):1–14.

[Meuleman et al., 2020] Meuleman, W., Muratov, A., Rynes, E., Halow, J., Lee, K., Bates, D., Diegel, M., Dunn, D., Neri, F., Teodosiadis, A., et al. (2020). Index and biological spectrum of human dnase i hypersensitive sites. *Nature*, 584(7820):244–251.

[Moore et al., 2020] Moore, J. E., Purcaro, M. J., Pratt, H. E., Epstein, C. B., Shoresh, N., Adrian, J., Kawli, T., Davis, C. A., Dobin, A., et al. (2020). Expanded encyclopaedias of dna elements in the human and mouse genomes. *Nature*, 583(7818):699–710.
